# Clinical study of tuberculosis in the head and neck region—11 years’ experience and a review of the literature

**DOI:** 10.1038/s41426-017-0008-7

**Published:** 2018-01-10

**Authors:** Pai Pang, Weiyi Duan, Shuchun Liu, Shuang Bai, Yanan Ma, Ruiwu Li, Fayu Liu, Changfu Sun

**Affiliations:** 1 Department of Oromaxillofacial-Head and Neck Surgery, Department of Oral and Maxillofacial Surgery, School of Stomatology, China Medical University, No. 117, Nanjing North Street, Shenyang, Liaoning 110002 China; 2 The Library of Liaoning University of Traditional Chinese Medicine, No. 79, Chongshan Road East, Shenyang, Liaoning 110847 China; 3 Department of Biostatistics and Epidemiology, School of Public Health, China Medical University, No. 77 Puhe Road, Shenyang, Liaoning 110122 China

## Abstract

Tuberculosis (TB) is an infectious disease and major health concern. Head and neck tuberculosis (HNTB) is relatively rare, but can arise in many regions, including the lymph nodes, larynx, oral cavity and pharynx. We retrospectively reviewed the clinical records of 60 patients diagnosed with HNTB in our department between March 2005 and January 2016. A review and summary of previous HNTB articles published in PubMed since 1885 was also performed. The subjects consisted of 17 males and 43 females, and the average age of patients was 45 ± 14.67 years. The major clinical presentation was a lump or swelling, followed by an oral ulcer and skin fistula. The most common site of tuberculosis was in the cervical lymph node. Three patients also suffered from a malignant tumor in the head and neck region. A total of 980 papers involving 5881 patients were included in our literature review. The included subjects ranged in age from 15 months to 100 years with a male-to-female ratio of 1.5:1. The larynx (38.92%), cervical lymph nodes (38.28%) and oral cavity (9.92%) were the three most common development sites. 465 patients were positive according to a HIV test, and 40 patients had comorbidities with different types of tumors. Head and neck tuberculosis should always be considered during a differential diagnosis for lesions in the head and neck region. Early diagnosis and treatment can greatly enhance the therapeutic effect and patients’ quality of life.

## Introduction

Tuberculosis (TB) remains one of the world’s deadliest diseases and has surpassed AIDS as the leading cause of death due to infectious disease^1^. The incidence of TB decreased in the 1980s because of the development of the BCG vaccine, anti-TB chemotherapy and improvements in public health care. However, since 1985, the number of TB patients has gradually increased due to the increase of the global population and increasing amount of HIV-positive and multiple drug-resistant TB patients^2^. According to a World Health Organization (WHO) report, TB accounted for 10.4 million new cases and 1.8 million deaths worldwide in 2015^1^.

In addition to lung lesions, one-fifth of TB cases present as extrapulmonary lesions, among which the most common sites include the lymph nodes, peritoneum, ileocecal, hepatosplenic, genitourinary, central nervous system, and musculoskeletal regions^3–5^. Diagnosis of TB in these organs is often overlooked because of its atypical clinical manifestations. However, extrapulmonary tuberculosis (EPTB) still contributes to a substantial TB incidence, and under-diagnosis of EPTB is also likely to cause life-long sequelae and fatal complications^6,7^.

Head and neck tuberculosis (HNTB) accounts for 10% of TB patients^8^. HNTB can affect most organs in the head and neck region, such as the lymph nodes, larynx, middle ear, oral cavity and pharynx^9^. In general, entrance of *M. tuberculosis* into these regions is covered with epithelium mucosa; therefore, immunosuppression or a break in this natural barrier caused by trauma, inflammation, poor oral hygiene or preexisting lesions, such as leukoplakia, periapical granuloma, cysts and abscesses, could induce the occurrence of tuberculosis^10,11^. Because the early manifestation of HNTB is often similar to neoplasms or inflammation and because the systemic symptoms of tuberculosis may not be obvious, clinical consideration of HNTB usually occurs only after an ineffective anti-inflammatory treatment, biopsy, or even surgical resection^2,12–16^. Despite the large number of existing reports, the patient risk of medical injury and occupational exposure caused by a delayed diagnosis of HNTB does not seem to have decreased over the past decade^17,18^.

To better understand the clinical features and provide diagnostic information for HNTB, this study summarized HNTB patients hospitalized in our department over the past 11 years, generally reviewed previous HNTB articles published since 1885 and reported on a case.

## Materials and methods

This study consists of three parts. First, we retrospectively analyzed data from HNTB patients in our hospital over the past 11 years. Then, we reviewed the historical literature. Finally, we shared a typical case with 10 months of follow-up data.

### Patients and data collection

This study included 60 patients with HNTB who were diagnosed at the Stomatology Hospital of China Medical University between January 2005 and January 2016. The medical records were retrospectively reviewed, and the patients’ sex, age, developmental site, clinical presentation, duration from first visit to diagnosis, TB history, comorbidities (HIV and tumor) and imaging features of the lesion and chest were extracted. All patients were diagnosed by histopathological examination. This study was approved by the ethics committee of China Medical University, and written consent was obtained from the patient presented in this paper.

### Search strategy and selection criteria

To review cases with HNTB, we searched PubMed for papers that contained the search terms “(head and neck OR pharyngeal OR laryngeal OR oral) AND (tuberculosis OR tuberculous)” in the title and abstract. A manual search of the reference lists of the related articles was performed to identify missing papers. Articles of all languages reporting HNTB cases were included. Data, such as the first author, publication year, country, department, number of patient, sex and age distribution and location of the lesion, were extracted for further evaluation. The latest search date was the 5th of January 2017. Included papers that provided at least the patient number and location of the lesion were classified as “paper with data”. Otherwise, they were classified as “paper without enough data”.

### Data analysis

The data were recorded in a Microsoft Excel spreadsheet, and graphs were created using GraphPad Prism 5 (GraphPad Software, Inc., La Jolla, CA, USA). Statistical analysis was performed using SPSS 21.0 (SPSS, Inc., Chicago, IL, USA).

## Results

### Our series

**Fig. 1:**
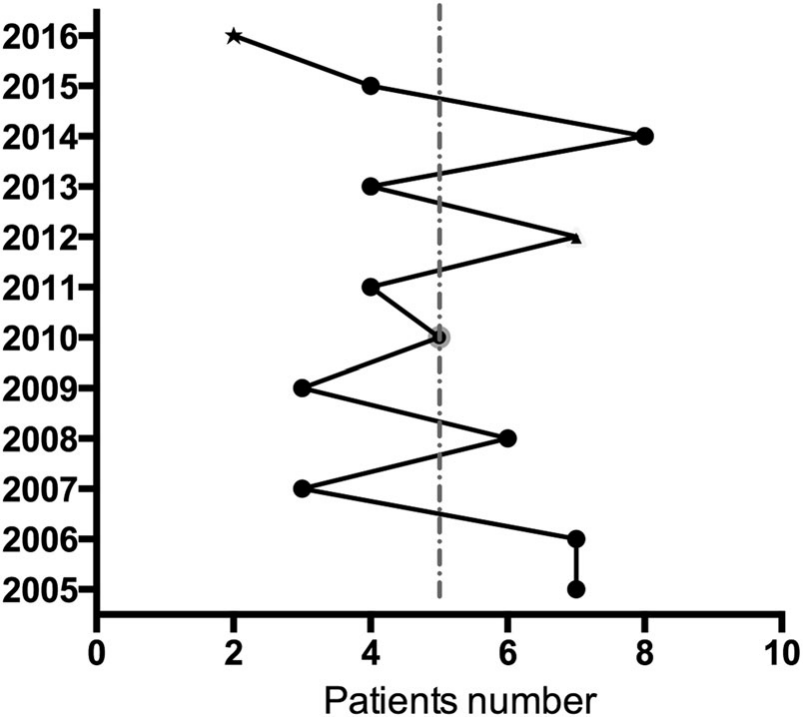
Number of patients by year—our series

The 60 subjects ranged in age from 5 to 76 years old (average 44.6 ± 14.8 years). Patients included 17 men and 43 women, with a male-to-female ratio of 1:2.53. Men ranged in age from 21 to 76 years (average 42.8 ± 17.4 years), and women ranged in age from 5 to 74 years (average 45.42 ± 13.74 years). From 2005 to 2016, the number of patients diagnosed every year ranged from 2 to 8, with an average of five patients per year (Fig. 1).

The duration from onset of symptoms to hospitalization ranged from 8 days to 47 years, with a median time of four months, and 65% of patients visited our hospital within six months. Eleven patients had a history of tuberculosis, and only one patient recalled symptoms of fever and weight loss. One patient had a clear contact history with family members diagnosed with TB. Nineteen patients (31.67%) showed old lesions in a chest X-ray, and two patients’ tuberculin skin tests (TST) were positive. All patients were finally diagnosed by a histopathological examination between 2 and 18 days (average 10.1 ± 3.74 days).

The major clinical manifestation was a lump or swelling in the head and neck region (56 patients), and the size of the lump ranged from 0.5 × 1 cm to 5 × 6 cm by palpation. Forty-five, eight and one patient received ultrasonography, CT and MRI for confirmation, respectively. Another three patients presented with an ulceration in the oral cavity, and one patient presented with a skin fistula of the chin. The most common infected site was the cervical lymph node (71.67%), followed by the salivary gland (35%) and oral cavity (5%). 22 (36.67%) patients had superior cervical lymphatic node involvement. Three patients presented with enlarged lymph nodes that coexisted with the malignancy (one with adenocarcinoma of the parotid gland, one with squamous cell carcinoma of the tongue and one with gingival carcinoma). 56 patients were tested for HIV infection, and all of them were negative. 30 patients were suspected to have multiple lesions. Once the diagnosis was made, patients were transferred to the infectious disease center for systematic anti-tuberculous treatment.

### Review of the literature

**Fig. 2:**
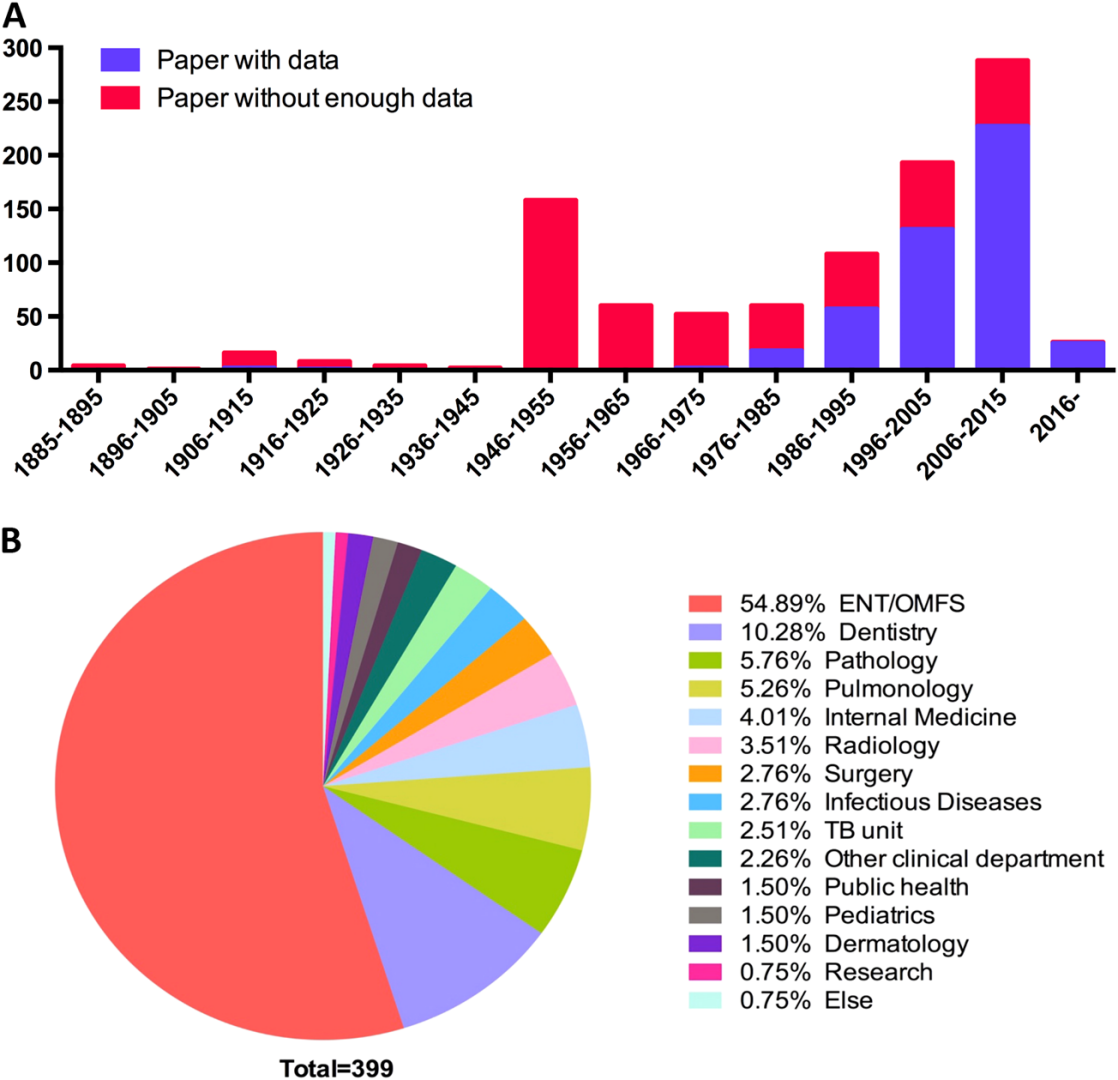
**Publication timeline and reporting department from the literature review**. **a** Number of publications by year. **b** Distribution of the reporting department. ENT ear-nose-throat; OMFS oral and maxillofacial surgery

We first retrieved 3290 papers published between 1885 and 2017 from PubMed and manual literature searches. After further evaluation, 980 papers (463 with data and 517 without enough data) with 5881 patients were included. Publication reached a peak at 1946–1955, then decreased and increased again each year since 1975 (Fig. 2a). Cases of HNTB were mainly reported by the ear-nose-throat (ENT)/oral and maxillofacial surgery (OMFS) (54.89%) department, followed by the dentistry (10.28%) and pathology (5.76%) departments (Fig. 2b).

**Fig. 3:**
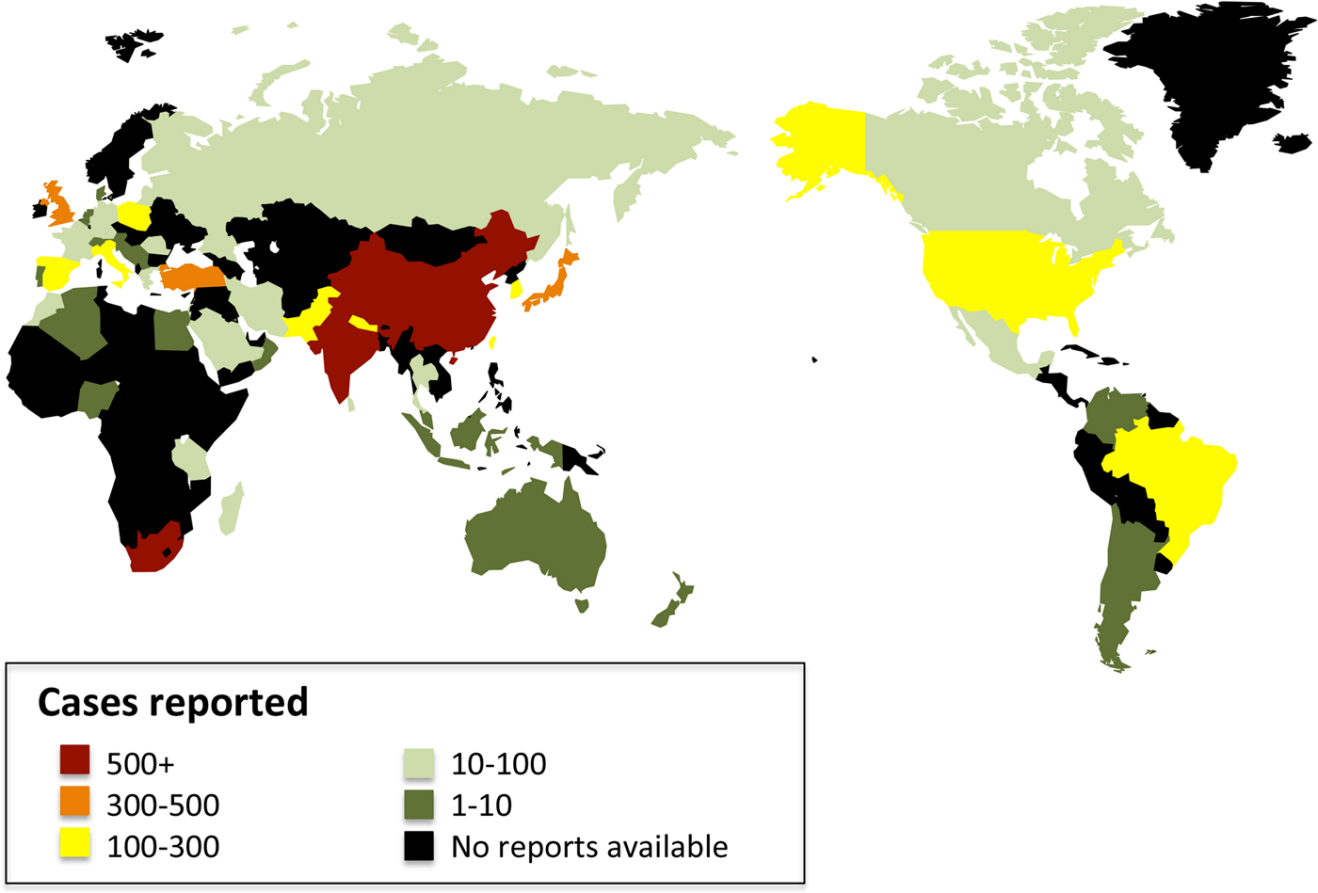
Geographical distribution of HNTB patients from the literature review

**Fig. 4:**
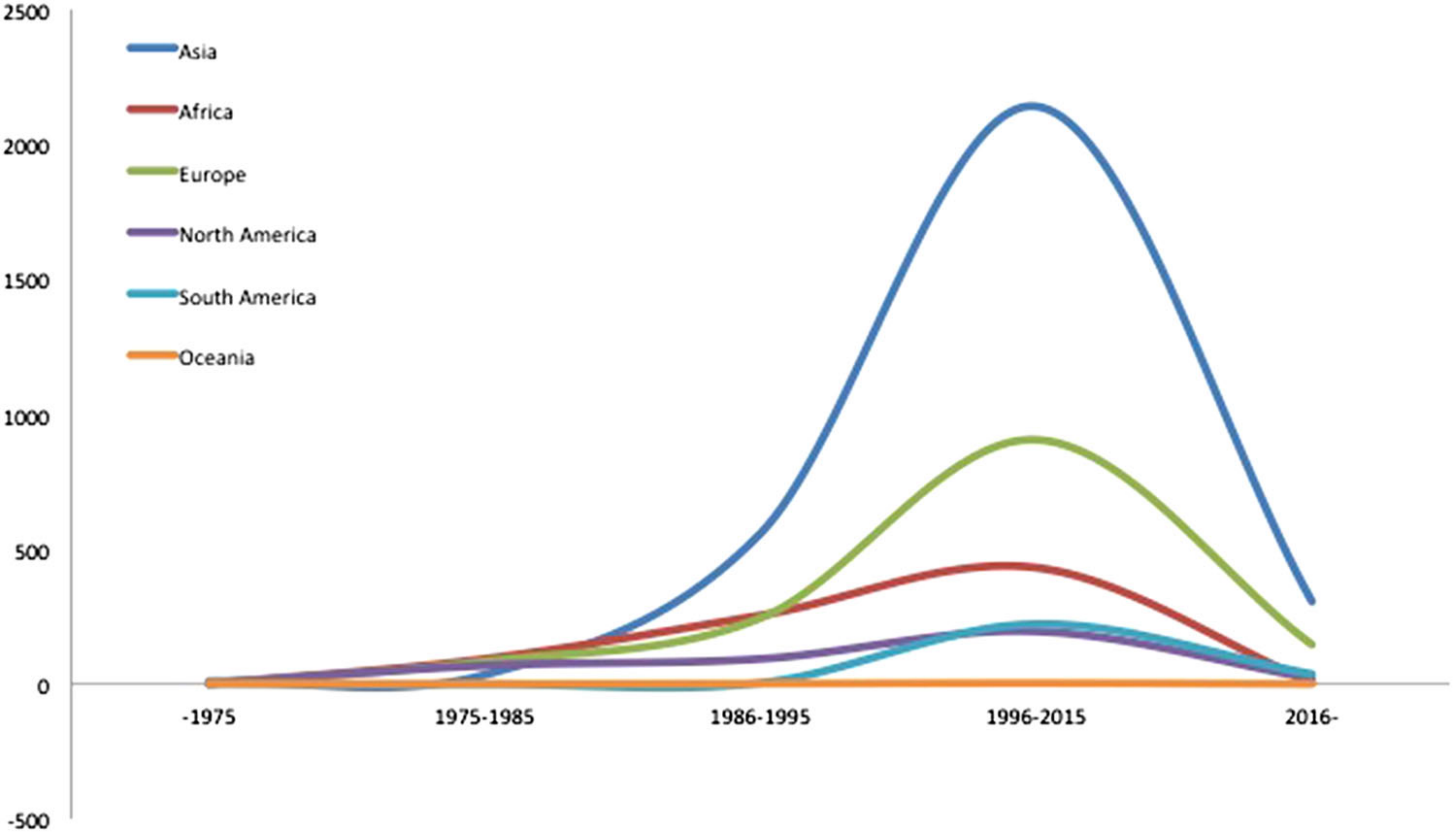
Annual prevalence of HNTB by continent from the literature review

**Fig. 5:**
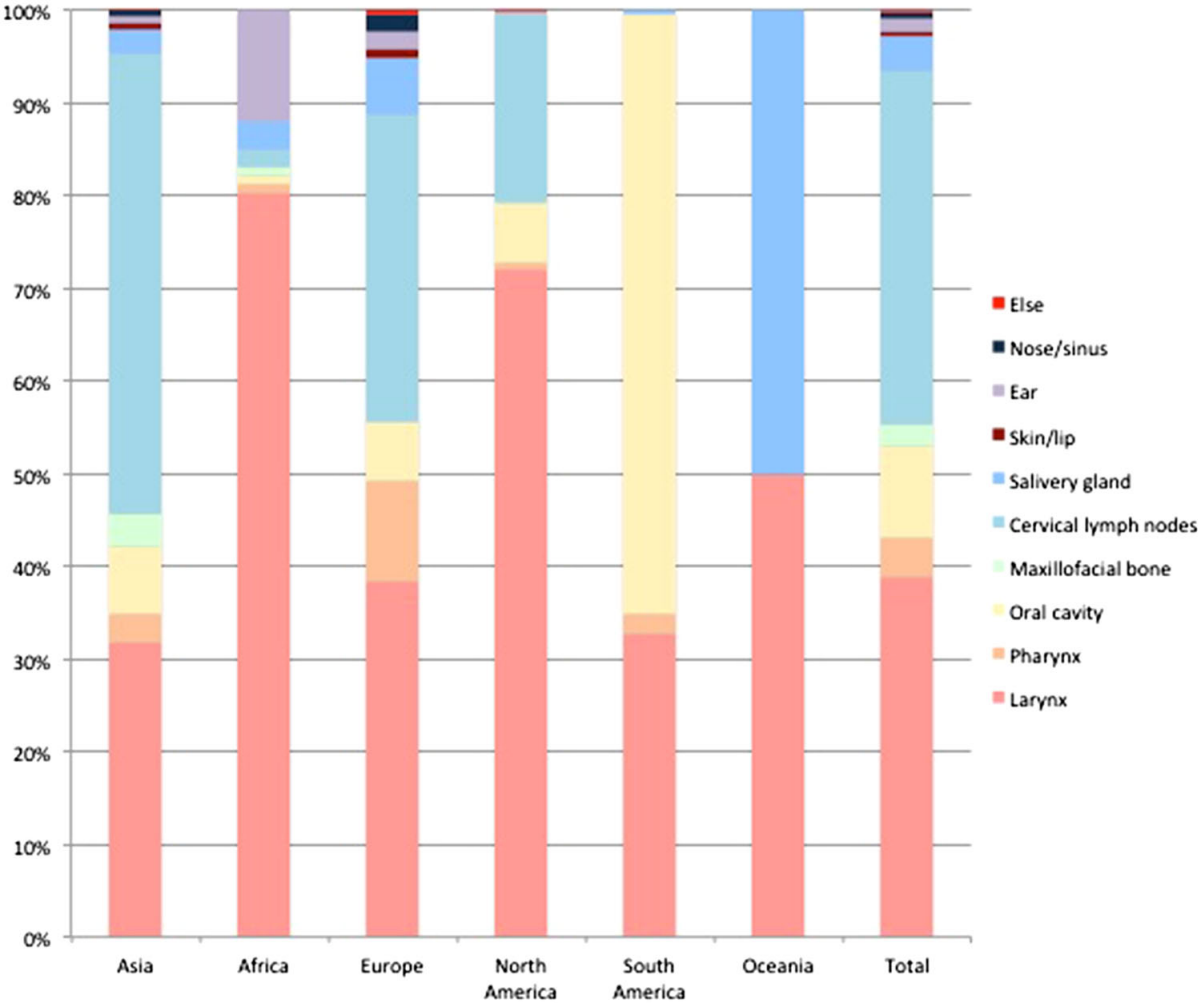
Organ distribution of HNTB by continent from the literature review

From the papers with data, the age of the included subjects ranged from 15 months to 100 years. The male-to-female ratio was 1.5:1. The geographical distribution of the reported patients showed that Asia reported the most cases over the past 40 years, followed by Europe and Africa, and the countries that reported the most cases were mainly located in Asia and Africa (Fig. 3). In general, the reported patient number gradually increased until 2015 (Fig. 4). The TB-infected sites varied in the different continents, and the larynx (38.92%), cervical lymph nodes (38.28%) and oral cavity (9.92%) were the three most common sites (Fig. 5). 465 patients were positive according to an HIV test. 40 patients had comorbidities with different types of tumors (Table 1).

**Table 1:** Reports of patient with concurrent HNTB and tumor

First author	Publication year	Country	Patients reported	Location of TB lesions	Patients coexist tumor	Comorbidity
Malignancy						
Manolidis S	1993	Canada	20	Head and neck region	1	Nasopharyngeal carcinoma
Grobholz R	1997	Germany	1	Cervical lymph nodes	1	Thyroid carcinoma
Plaza Mayor G	1998	Spain	2	Larynx	2	Laryngeal carcinoma
Weiler Z	2000	Israel	21	Cervical lymph nodes	1	Gastric carcinoma
Landa LE	2002	Venezuela	1	Salivary gland	1	Buccal space carcinoma
Miura K	2003	Japan	1	Larynx	1	Laryngeal angiosarcoma
Bartnik W	2004	Poland	8	Larynx/pharynx	1	Epiglottic cancer
Nishiike S	2006	Japan	1	Larynx	1	Laryngeal carcinoma
Satoh S	2007	Japan	1	Cervical lymph nodes	1	Thyroid gland carcinoma
Prasad KC	2007	India	165	Head and neck region	3	Laryngeal malignancy
Wang WC	2009	China	20	Head and neck region	2	Metastatic cervical lymph node carcinoma
Dokuzlar U	2009	Turkey	8	Cervical lymph nodes	8	Metastatic cervical lymph node carcinoma
Al-Zahid S	2010	UK	1	Cervical lymph nodes	1	Laryngeal carcinoma
Bagga P	2012	India	1	Oral cavity	1	Tongue cancer
Wang SY	2013	China	47	Head and neck region	1	Laryngeal carcinoma
Bruzgielewicz A	2014	Poland	73	Head and neck region	6	5 Laryngeal cancer;1 oropharyngeal cancer
Caroppo D	2015	Italy	1	Cervical lymph nodes	1	Metastatic cervical lymph node carcinoma
Lucena MM	2015	Brazil	24	Larynx	1	Skin cancer
Pajor AM	2016	Poland	71	Head and neck region	1	Laryngeal carcinoma
Benign tumor						
Suoglu Y	1998	Turkey	6	Parotid gland	1	Warthin tumour
Watanabe M	2001	Japan	2	Salivary gland	2	Warthin tumour
Al Bisher	2010	Saudi Arabia	1	Parotid gland	1	Pleomorphic adenoma
Wu KC	2012	China	1	Parotid gland	1	Warthin tumor
Ulusan	2013	Turkey	1	Parotid gland	1	Warthin tumor

### Case report

**Fig. 6:**
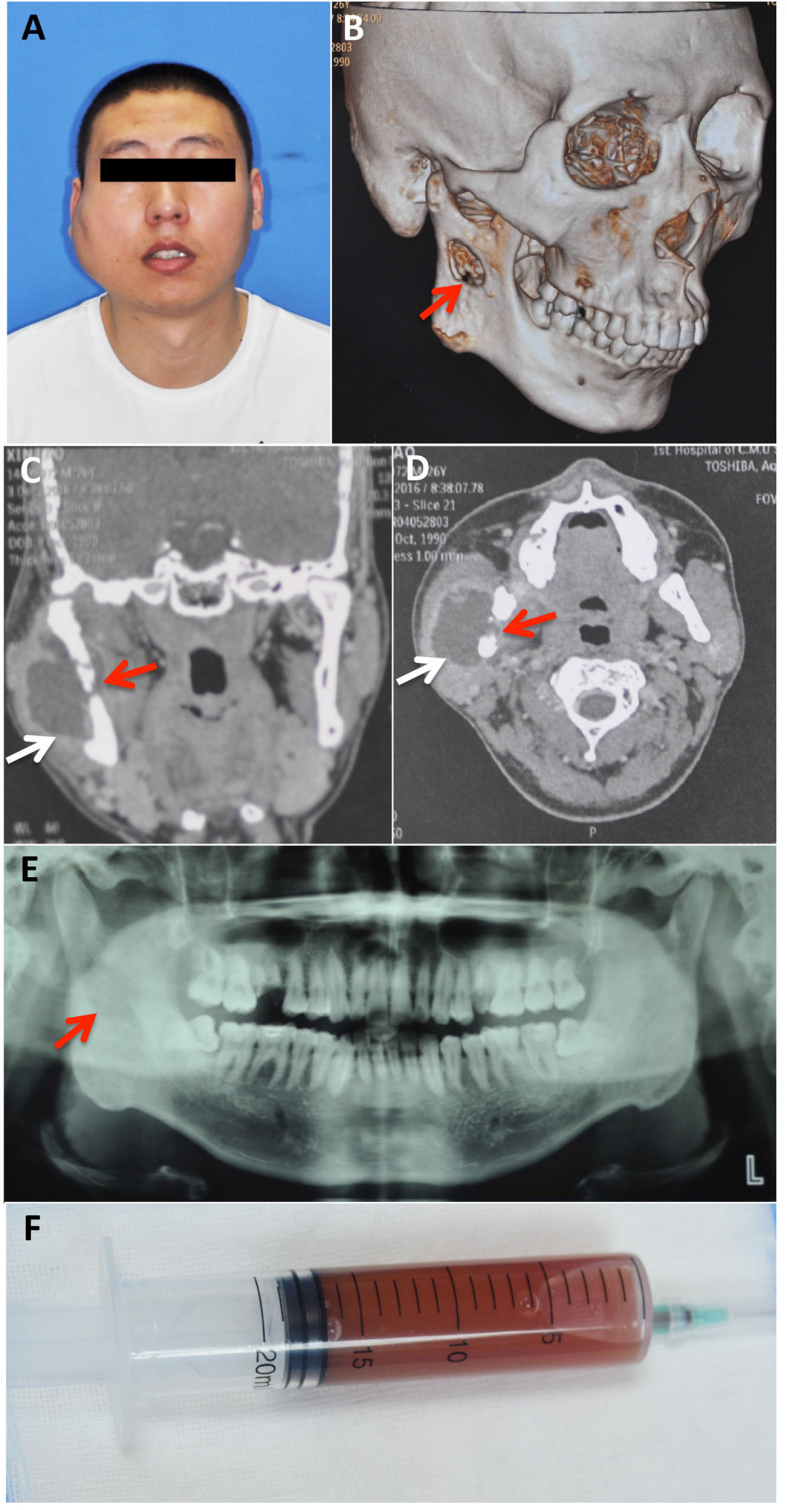
**Clinical material of a HNTB patient—the case report.** **a** Frontal photograph. **b**–**d** Computed tomography results. **e** Panoramic tomography results. **f** Liquid aspirate from the lesion site. White arrows: HNTB lesion; Red arrow: mandibular involvement

**Fig. 7:**
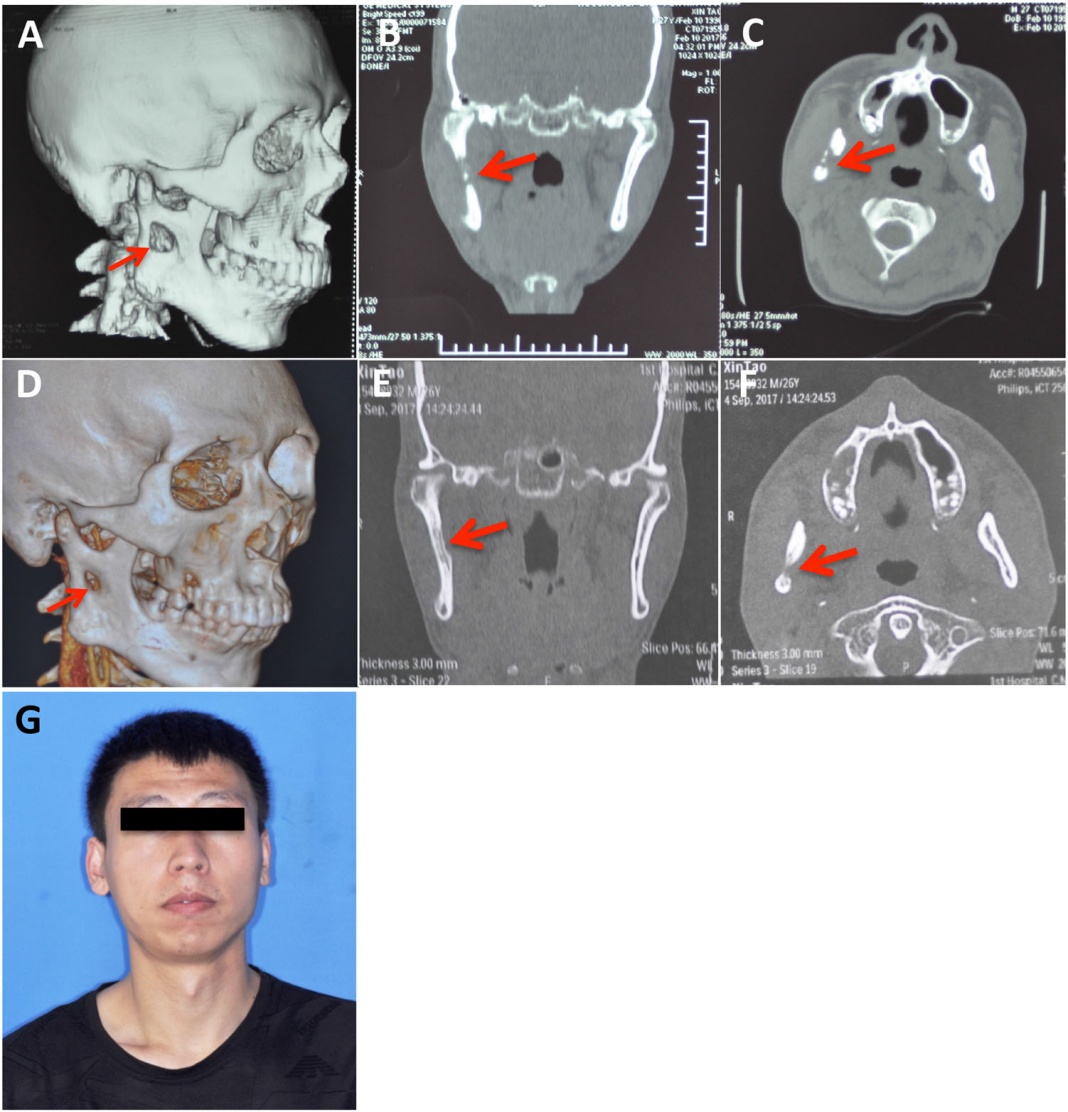
**Follow-up material of the HNTB patient—the case report**. **a**–**c** Computed tomography results after two months of treatment. **d**–**f** Computed tomography results after ten months of treatment. **g** Frontal photograph after ten months of treatment. Red arrow: mandibular involvement

A 26-year-old man presented to the oral and maxillofacial surgery department with swelling on the right side of his face. He had a four-year history of pulmonary tuberculosis and was diagnosed with tuberculous pleuritis because of pleural effusion three years ago. The patient received anti-TB treatment and had not had any symptoms for the past two years. Two months previously, he began experiencing swelling of the right side of his face and numbness of his right lower lip without fever, night sweats or weight loss. He received anti-inflammatory treatment with cephalosporin and levofloxacin at the community clinic for more than one week and the symptoms did not alleviate. A physical examination showed a fixed mass in the right parotid region without tenderness (Fig. 6a) and an axillary mass of approximately 2 × 3 cm. Computed tomography and pantomography showed a soft tissue lesion involving the ramus of the mandible (Fig. 6b–e). A puncture of the lesion resulted in the aspiration of 17 ml of yellow turbid liquid with a little blood (Fig. 6f). A mycobacterium culture with the liquid had a negative result. Considering his TB history, the patient was transferred to a chest hospital and received a tentative treatment with isoniazid (INH), rifampicin (RIF), pyrazinamide (PZA), ethambutol (EMB), capreomycin and liver-protecting medicine. After two months of treatment, the patient showed a healing trend in the mandible (Fig. 7a–c). After ten months of treatment, his facial appearance returned to normal and obvious bone healing was found (Fig. 7d–g).

## Discussion

Although an increasing number of cases have been reported in the past few decades, HNTB remains a “diagnosis dilemma” for many doctors. HNTB patients may present with no specific pathognomonic sign, have less severe systemic symptoms and have negative results for some tubercular tests. In the review of 5881 HNTB patients, we observed that 73% of the identified papers reported less than three cases, 88.12% of the identified papers reported less than 30 cases, and many recent reports still considered their cases to be “rare entity” or “unusual cause” of clinical symptoms. This suggests that the lack of coherent studies and systematic summaries has contributed to the stagnation of early diagnosis of HNTB.

In usual cases, HNTB presents as a non-healing ulceration or mass that mimics a tumor or malignant metastasis^14,19^, whereas specific symptoms may occur according to the pathogenic sites. Tuberculosis in the oral cavity and pharynx could be accompanied with halitosis, dysphonia and sore throat^12,16^. Laryngeal TB may cause cough, odynophagia, hoarseness of the voice, and even dyspnea^12,20^. Aural TB could be concomitant with otalgia, tinnitus, hearing loss, and facial nerve palsy^12,21^. Nasal TB mainly causes nasal obstruction, epistaxis, and purulent or bloody discharge, and when the paranasal sinus is involved, there may be bone destruction and optical symptoms, such as diplopia and exophthalmos^8,12^. HNTB should be differentially diagnosed with other infectious diseases, such as syphilis, leprosy, actinomycosis, coccidioidomycosis, blastomycosis and leishmaniosis, and non-infectious diseases, including carcinoma, lymphoma, angiofibroma, sarcoidosis, periarteritis nodosa, Castle man’s disease, amyloidosis and Wegener’s granulomatosis^8,13,16^. Simultaneously, HNTB may reflect general body conditions, such as a comorbidity with malignancy (5% in our cases and 0.60% in review cases) and HIV infection (0% in our cases and 7.91% in review cases); therefore, concurrent systemic diseases should also be identified when a diagnosis of HNTB is determined.

In our cases, the most common site of HNTB was the cervical lymph nodes, which coincide with many recent reports^12,21,22^. However, a total review of the historical literature showed a similar ratio of laryngeal and cervical TB. This discordance may be for two major reasons. First, most serial HNTB cases were reported by otorhinolaryngologists or oral and maxillofacial surgeons. However, approximately 48% of laryngeal TB patients had concurrent pulmonary TB^22^ and were separately reported by the departments of internal medicine or infectious disease. Second, pulmonary TB with laryngeal TB has had a higher prevalence and reporting chance in the past. In fact, more than 62.9% of laryngeal TB patients were reported before the year 2007. In the study by Pajor, which included patients from 1978 to 2013, the larynx was also found to be the most common site of HNTB^18^. Therefore, studies only including patients in the most recent ten years may reach a different conclusion.

Unlike pulmonary TB, only 20% of HNTB patients may show typical symptoms of cough, fever or night sweats^12^. For patients with primary HNTB lesions, the signs may be too minor to notice. In our case series, only one patient recalled experiencing fever and weight loss. For these patients, a clinical examination may more likely be negative. Meanwhile, ulceration lesions accompanied by swelling of the lymph nodes often gives the clinical impression of a malignancy^23^. These factors may cause a diagnostic dilemma. For diagnosing HNTB, an imaging examination, such as ultrasonography and computed tomography, can provide a general idea of the location, size and number of TB lesions. Other examinations, such as the TST and interferon-gamma release assays (IGRAs), can help make a diagnosis; however, false negative results have been reported^12,21^. According to previous reports, the TST positive rate is only 53%^12^ and is much lower in patients with local primary TB lesions. Compared to TST, IGRA has been demonstrated by evidence-based research to be more specific and have higher positive and negative predictive values^24,25^. Additionally, IGRA is more accurate than TST and has a better sensitivity in immunosuppressed patients^26^. However, neither test can distinguish latent TB from active TB, and the replacement of TST by IGRA is not recommended in middle-to-high incidence regions^27^. Ziehl-Neelsen staining for acid-fast bacilli is also a common method in the clinic; however, only 27–60% of TB patients give positive results because of the selective scarcity of bacilli within tissue^10^. Moreover, a positive result does not support a 100% diagnosis of tuberculosis^28^. Culturing *M. tuberculosis* remains the gold standard, but it requires a period of 8–10 weeks and the sensitivity varies depending on the host and sample preparation^3^. Polymerase chain reaction can provide a sensitivity of 45–75% and specificity of 97.3–100% in aspirated samples of cervical adenopathies^22^. The Gene Xpert MTB/RIF can provide a diagnosis of tuberculosis in less than 2 h with a sensitivity of 89% in smear-positive patients and 67% in smear-negative patients^27^. Because HNTB patients often turn to surgical departments, the first diagnosis is often made through a histopathological examination of a surgical specimen^21^. For patients who have similar clinical symptoms but ambiguous test results, a good response to tentative chemotherapy against TB can also lead to a final diagnosis. Meanwhile, next-generation nucleic acid amplification tests (NAATs), microscopy and blood tests have been considered to meet the needs for a sensitive, low-cost and high-throughput screening method of tuberculosis^29^.

Once diagnosed, traditional anti-tuberculous treatment, including INH, RIF, PZA, and EMB, can have favorable curative effects. Quadritherapy with INH, RIF, EMB and PZA for 2 months, followed by INH and RIF for 4–7 months, is generally accepted^22^. Waldron et al.^30^ used a standard regimen of INH, RIF, and PZA for no less than six months and added streptomycin during the first 2–3 months for nasopharyngeal tuberculosis. Most patients reported a satisfactory recovery; however, once unfavorable sequelae occur in the head and neck region, surgery becomes necessary to relieve life-threatening complications^13,31^.

In conclusion, this study summarized 60 patients with head and neck tuberculosis in our 11-year experience, reviewed and analyzed historical literature for HNTB and reported on a case. Head and neck tuberculosis should always be considered during differential diagnosis for lesions in the head and neck region. Early diagnosis and treatment can largely enhance patients’ quality of life, reduce potential disease transmission and protect health care providers.

## Electronic supplementary material

Supplementary material

## Electronic supplementary material

**Supplementary Information** accompanies this paper at 10.1038/s41426-017-0008-7.) contains supplementary material.
