# Supplementary material for: Clinical study of tuberculosis in the head and neck region—11 years’ experience and a review of the literature
Source: Emerg Microbes Infect. 2018 Jan 10;7:4. doi: 10.1038/s41426-017-0008-7 (PMC5837174; doi:10.1038/s41426-017-0008-7)
Supplement: Supplementary file 1 — Supplementary material [file 41426_2017_8_MOESM1_ESM.docx]

**Supplementary material S1: References with cases of the literature review not cited in the text**

1. Pilcher JE. III. Tracheotomy in Laryngeal Stenosis of Tuberculous Origin. *Ann Surg* 1885; **1:** 144-151.
2. Shepherd FJ. Excision of the Tongue Followed by Death from Acute Miliary Tuberculosis. *Annals of surgery* 1888; **8:** 368-371.
3. A Discussion on the Treatment of Laryngeal Disease in Tuberculosis. *Br Med J* 1890; **2:** 611-622.
4. Kidd P. On a Peculiar Obstructive Form of Laryngeal Tuberculosis which Simulates Bilateral Abductor Paralysis. *Br Med J* 1890; **1:** 715-717.
5. Glasgow WC. Some Experiments in the Treatment of Pulmonary and Laryngeal Tuberculosis with the Concentrated Electric Arc Light. *Trans Am Climatol Assoc* 1903; **19:** 110-121.
6. Semon F. Remarks on the therapeutic value of complete vocal rest during the sanatorium treatment of laryngeal tuberculosis. *Br Med J* 1906; **2:** 1623-1627.
7. Welty CF. Indications and Contra-Indications for Intra-Laryngeal Operation in Tuberculosis of Larynx. *Cal State J Med* 1906; **4:** 88-90.
8. Bardswell N, Adams B. Remarks on complete vocal rest during the sanatorium tratment of laryngeal tuberculosis. *Br Med J* 1907; **1:** 1350-1353.
9. Felkin HG. A contribution to the value of complete vocal rest as an aid to recovery from laryngeal tuberculosis and allied conditions during sanatorium treatment. *Br Med J* 1907; **1:** 1421-1423.
10. Donelan J. Laryngeal Tuberculosis in a Man aged 43. *Proc R Soc Med* 1909; **2:** 141-142.
11. Semon F. Remarks on a Case of Pneumococcus Invasion of the Throat upon which Laryngeal and Pulmonary Tuberculosis Supervened. *Br Med J* 1909; **1:** 1525-1528.
12. Brough DD. The Importance of Registration and Control of Cases of Laryngeal and Pulmonary Tuberculosis. *Am J Public Hygiene* 1910; **20:** 166-180.
13. Grant JD. Tuberculosis of the Larynx, with Extreme Odynphagia, relieved by Injection of Alcohol into the Left Superior Laryngeal Nerve. *Proc R Soc Med* 1910; **3:** 152-154.
14. Horne WJ. Laryngeal Tuberculosis in a Boy, aged 10. *Proc R Soc Med* 1911; **4:** 116.
15. Horsford C. Laryngeal Tuberculosis-Cure. *Proc R Soc Med* 1911; **4:** 43.
16. Donelan J. Laryngeal Tuberculosis. *Proc R Soc Med* 1912; **5:** 18-19.
17. Faulder TJ, Harmer WD. Pharyngeal Tuberculosis; Tuberculous Lesions of other Parts; Treatment by Injections of Tuberculin. *Proc R Soc Med* 1912; **5:** 55-57.
18. Davis ED. A Post-mortem Specimen of Laryngeal Tuberculosis. *Proc R Soc Med* 1913; **6:** 110.
19. Davis ED. An Unusual Case of Laryngeal Tuberculosis. *Proc R Soc Med* 1913; **6:** 130.
20. Davis ED. Advanced Laryngeal Tuberculosis treated by Tracheotomy and Curetting. *Proc R Soc Med* 1914; **7:** 200.
21. Thomson S. Three years' sanatorium experience of laryngeal tuberculosis. *Br Med J* 1914; **1:** 801-803.
22. Otani M. The significance of the epithelial cells and saprophytes in sputum. *J Exp Med* 1917; **25:** 333-339.
23. Grant JD. Case of Fibro-papilloma of Larynx simulating Laryngeal Tuberculosis in a Middle-aged Soldier; Pendulous Epiglottis; Removal of Growth by means of Snare and Forceps. *Proc R Soc Med* 1918; **11:** 64-65.
24. Moore I. Left Recurrent Laryngeal Nerve Paralysis in a Patient suffering from Tuberculosis. *Proc R Soc Med* 1918; **11:** 167-170.
25. Colledge L. Case of Laryngeal Tuberculosis. *Proc R Soc Med* 1920; **13:** 169-170.
26. Thomson S. Case of Subglottic Thickening, at first diagnosed as Primary Laryngeal Tuberculosis. *Proc R Soc Med* 1921; **14:** 64-65.
27. Franklin P. Two Cases of Pulmonary Tuberculosis with Laryngeal Symptoms. *Proc R Soc Med* 1923; **16:** 25-26.
28. Parfitt CD. Treatment of Laryngeal Tuberculosis in Sanatoria. *Trans Am Climatol Clin Assoc* 1923; **39:** 170-181.
29. Vlasto M. Case of Laryngeal Tuberculosis in a Child aged 4(1/2) years. *Proc R Soc Med* 1924; **17:** 53-54.
30. Parfitt CD. The Treatment of Laryngeal Tuberculosis in Sanatoria. *Can Med Assoc J* 1926; **16:** 1206-1210.
31. Howarth W. Laryngeal Lesion associated with Apparent Miliary Tuberculosis of the Lung. *Proc R Soc Med* 1927; **20:** 1088-1089.
32. Rubinstein C. The Treatment of Laryngeal Tuberculosis. *Cal West Med* 1930; **33:** 825-826.
33. Lowry E. Tracheotomy eleven years ago for Healed Laryngeal Tuberculosis, with Ankylosis of Cords: Further report on case previously shown. *Proc R Soc Med* 1934; **27:** 382.
34. The Problem of Early Laryngeal Tuberculosis: (Section of Laryngology). *Proc R Soc Med* 1937; **30:** 221-236.
35. Nikolskaia EN. Use of iono-galvanization with calcium chloride in laryngeal tuberculosis. *Probl Tuberk* 1945**:** 66.
36. Auerbach O. Laryngeal tuberculosis. *Arch Otolaryngol* 1946; **44:** 191-201.
37. Humphries MK, Jr. Laryngeal tuberculosis. *Dis Chest* 1946; **12:** 129-146.
38. Laryngeal tuberculosis. *Manit Med Rev* 1947; **27:** 233-235.
39. Edwards TA. Tracheotomy for tuberculous laryngeal stenosis, with case report. *Tubercle* 1948; **29:** 274-276.
40. Looper EA, Lyon IB. Laryngeal tuberculosis; observations based on an experience of 28 years with laryngeal tuberculosis. *Ann Otol Rhinol Laryngol* 1948; **57:** 754-768.
41. Mc GF, Broome LR. The treatment of laryngeal tuberculosis with streptomycin. *Va Med Mon (1918)* 1948; **75:** 129.
42. Papper EM, Rovenstine EA. Nerve block therapy for pain of laryngeal tuberculosis. *N Y State J Med* 1948; **48:** 622.
43. Withers BT. Streptomycin in treatment of laryngeal tuberculosis. *Ann Otol Rhinol Laryngol* 1948; **57:** 769-783.
44. Bachi S. [Laryngeal tuberculosis in childhood: results of streptomycin therapy.] *Minerva Pediatr* 1949; **1:** 371. Italian.
45. Cojazzi G. [Oral tuberculosis treated with locally infiltrated streptomycin.] *Riv Ital Stomatol* 1949; **4:** 1177-1188. Italian.
46. Gandullo Solsona L. [Our experience with laryngeal tuberculosis; before and after streptomycin.] *Med Cir Guerra* 1949; **11:** 511-514. Spanish.
47. Johansen H, Kiaer W. Contact ulcers and laryngeal tuberculosis. *Arch Otolaryngol* 1949; **50:** 264-283.
48. Looper EA. Laryngeal tuberculosis. *Arch Otolaryngol* 1949; **49:** 117.
49. Meda P, Giorgi P. [Immediate and long-term results of streptomycin therapy in pharyngo-laryngeal tuberculosis.] *Osp Maggiore* 1949; **37:** 419-426. Italian.
50. Von Fraenkel PH. Oro-laryngeal tuberculosis; report of a case. *U S Nav Med Bull* 1949; **49:** 536-541.
51. Arnaud G, Alcalays V. Tuberculous laryngeal stenosis and streptomycin. *Ann Otolaryngol* 1950; **67:** 161-165.
52. Bystrzanowska T. Therapeutic results in laryngeal tuberculosis. *Gruzlica* 1950; **18:** 236-246.
53. Cornwall VC. Laryngeal tuberculosis associated with pulmonary tuberculosis; its incidence, prognosis and treatment. *Med Press* 1950; **223:** 361-364.
54. Cristiani M, Pifferi G. Para-aminosalicylic acid in the treatment of laryngeal tuberculosis. *Minerva Med* 1950; **41:** 742-747.
55. De Giorgi L, Scalise A. Problem of laryngeal tuberculosis and pregnancy after the advent of streptomycin therapy. *Arch Ostet Ginecol* 1950; **55:** 168-178.
56. Dupont P. Laryngeal tuberculosis. *Acta Otorhinolaryngol Belg* 1950; **4:** 551-592.
57. German T, Nako A. Observations on the streptomycin treatment of laryngeal tuberculosis. Some aspects on the streptomycin-vestibular damages. *Acta Otolaryngol* 1950; **38:** 97-107.
58. Ginader R, Rickmann L. Chemotherapy of laryngeal tuberculosis. *Tuberkulosearzt* 1950; **4:** 340-345.
59. Huebschmann P. Laryngeal tuberculosis and TB1. *Z Laryngol Rhinol Otol* 1950; **29:** 285-290.
60. Klionskii EE, Il'Ina VA. Tissue therapy in pulmonary and laryngeal tuberculosis. *Probl Tuberk* 1950; **3:** 57-59.
61. Obtulowicz T. Laryngeal tuberculosis with special reference to streptomycin therapy. *Gruzlica* 1950; **18:** 102-117.
62. Oppenheim H, Livingston CS, Nixon JW, Miller CD. Streptomycin therapy in oral tuberculosis. *AMA Arch Otolaryngol* 1950; **52:** 910-929.
63. Osman DA, Calderin VO, Byrd DL. A case of oral tuberculosis associated with cheesy conglomerate tuberculosis of the prostate terminating in miliary dissemination. *Oral Surg Oral Med Oral Pathol* 1950; **3:** 1390-1399.
64. Piaget F, Rocher G, Robert A. Statistical data on the frequency and evolution of laryngeal tuberculosis before the use of streptomycin. *J Med Lyon* 1950; **31:** 829-832.
65. Rosenblat MS. Streptomycin therapy of tuberculosis of pharynx, larynx and oral cavity. *Vestn Otorinolaringol* 1950; **12:** 27-33.
66. Schurmann F, Radenpach KL. Tuberculosis and thiosemicarbazone Tb I/698; therapeutic results in pulmonary and secondary laryngeal tuberculosis. *Schweiz Z Tuberk* 1950; **7:** 99-114.
67. Valdez HJ. Tyrothricin in laryngeal tuberculosis. *Dia Med* 1950; **22:** 2539-2540.
68. Withalm A. Experiences with thiosemicarbazone therapy of laryngeal tuberculosis. *Wien Med Wochenschr* 1950; **100:** 778-779.
69. Bajkay T. Streptomycin therapy of laryngeal tuberculosis. *Pract Otorhinolaryngol (Basel)* 1951; **13:** 76-84.
70. Bajkay T. Thiosemicarbazone in the treatment of laryngeal tuberculosis. *Orv Hetil* 1951; **92:** 512-515.
71. Chebotarev AI. Tissue therapy as a method of control of dysphagia in laryngeal tuberculosis. *Vestn Otorinolaringol* 1951; **13:** 65-66.
72. Chvojka J. Experiences with streptomycin therapy of pharyngo-laryngeal tuberculosis. *Cas Lek Cesk* 1951; **90:** 1372-1377.
73. Cody CC 3rd. Streptomycin therapy in laryngeal tuberculosis. *AMA Arch Otolaryngol* 1951; **53:** 1-26.
74. Fremel F. Laryngeal tuberculosis. *Wien Med Wochenschr* 1951; **101:** 936-937.
75. Fridkin MM. Treatment of laryngeal tuberculosis by prolonged novocain block. *Probl Tuberk* 1951; **1:** 39-40.
76. Gammarrota V. Anatomical aspects of tuberculosis of the epiglottis treated with streptomycin. *Clin Nuova Rass Prog Med Int* 1951; **13:** 369-382.
77. Gammarrota V. Case of tuberculous laryngitis treated with streptomycin. *Clin Nuova Rass Prog Med Int* 1951; **13:** 409-418.
78. Hammelburg E. A case of tuberculous otitis media following BCG oral administration. *Ned Tijdschr Geneeskd* 1951; **95:** 3498-3502.
79. Herold K. Local therapy of laryngeal tuberculosis. *Tuberkulosearzt* 1951; **5:** 405-406.
80. Hofer G, Kroath F. Bayer's TB I/698 in the treatment of laryngeal tuberculosis. *Monatsschr Ohrenheilkd Laryngorhinol* 1951; **85:** 62-63.
81. Hogg JC. Tuberculosis of the upper respiratory tract. *Practitioner* 1951; **167:** 596-606.
82. Lederer L, Gulich S. Experiences with conteben in the treatment of laryngeal tuberculosis. *Med Klin* 1951; **46:** 10-14.
83. Levinsky L. Streptomycin therapy of tuberculosis of the oral cavity. *Cas Lek Cesk* 1951; **90:** 961-968.
84. Mescolini G. Cerebrospinal fluid in tuberculous meningitis treated with streptomycin; primary and secondary clinical forms with other localizations (pulmonary, laryngeal and osteoarticular). *Lotta Tuberc* 1951; **21:** 559-575.
85. Oppenheim H, Livingston CS, Nixon JW, Miller CD. Streptomycin therapy in oral tuberculosis. *Oral Surg Oral Med Oral Pathol* 1951; **4:** 1389-1405.
86. Podgaetskii GB, Sheinman NS. Immediate results in the treatment of upper respiratory tuberculosis with paraaminosalicylic acid. *Vestn Otorinolaringol* 1951; **13:** 86-87.
87. Raspopov AP. Early acute, and minimal chronic forms of laryngeal tuberculosis. *Vestn Otorinolaringol* 1951; **13:** 35-39.
88. Rocheta J, Batoreo A, De Oliveira Y, De Almeida N. Thiosemicarbazones in the treatment of laryngeal, bronchial and pulmonary tuberculosis. *Gaz Med Port* 1951; **4:** 865-876.
89. Shengold MA, Sheingold H. Oral tuberculosis. *Oral Surg Oral Med Oral Pathol* 1951; **4:** 239-250.
90. Simpo K, Tsukada H. Pathological anatomical studies on the antituberculous treatment with streptomycin. III. On the influences of the streptomycin treatment upon intestinal tuberculosis and larynx tuberculosis. *Kekkaku* 1951; **26:** 236-241; transl 358-239.
91. Tapia Acuna R. Considerations on diagnosis and treatment of laryngeal tuberculosis. *Rev Med Cienc Afines* 1951; **9:** 483-486.
92. Vinokurova BL. Local application of streptomycin in oral and laryngeal tuberculosis. *Probl Tuberk* 1951; **1:** 67-68.
93. Wolf HE. Treatment of laryngeal tuberculosis with conteben, with Tb 6, streptomycin and PAS. *Z Laryngol Rhinol Otol* 1951; **30:** 241-242.
94. Zanzucchi G. Clinical experiences with para-aminosalicylic acid in laryngeal tuberculosis. *Gazz Med Ital* 1951; **110:** 262-266.
95. Zielinski BZ. Streptomycin and laryngeal surgery. *Rev Laryngol Otol Rhinol (Bord)* 1951; **72:** 149-160.
96. Current treatment of tuberculosis of the larynx. *Eye Ear Nose Throat Mon* 1952; **31:** 208-209.
97. Ben Abda M. Cancer and laryngeal tuberculosis. *Rev Laryngol Otol Rhinol (Bord)* 1952; **73:** 127-180.
98. Bertelli JA. Antibiotics in the treatment of extrapulmonary tuberculosis; present state of the treatment of aural, nasal, and laryngeal tuberculosis. *Prensa Med Argent* 1952; **39:** 3082-3086.
99. Bettington RH. Laryngeal tuberculosis. *Med J Aust* 1952; **1:** 8-10.
100. Cristiani M. Association of laryngeal cancer, pulmonary syphilis, and lymph node tuberculosis in the same patient. *Otorinolaringol Ital* 1952; **20:** 569-575.
101. De Michelis B. A case of oral tuberculosis treated with streptomycin and para-aminosalicylic acid. *Minerva Stomatol* 1952; **1:** 139-140.
102. Degos R, Delort J, Garlopeau F. Extensive subacute ulcerous tuberculosis of the oral cavity; very rapid cicatrization by isonicotinic acid hydrazide. *Bull Soc Fr Dermatol Syphiligr* 1952; **59:** 447-448.
103. Fel'Dman SP. Symptoms of early stages of laryngeal tuberculosis. *Vestn Otorinolaringol* 1952; **14:** 58-62.
104. Fenton RA. Laryngeal tuberculosis. *Trans Am Laryngol Rhinol Otol Soc* 1952; **1:** 16-20.
105. Ferrari A. Biagio Gastaldi, pioneer of phthisiology, neglected by medical history, and on his new method for the treatment of pulmonary and laryngeal tuberculosis. *Riv Stor Sci Mediche Nat* 1952; **43:** 360-361.
106. Gardenghi G. Therapy of laryngeal tuberculosis with isonicotinic acid hydrazide. *Boll Mal Orecch Gola Naso* 1952; **70:** 384-391.
107. Gilbert JG, Aronoff JS. Treatment of tuberculosis of larynx; comparative study of streptomycin, paraaminosalicylic acid, and tibione. *AMA Arch Otolaryngol* 1952; **56:** 435-447.
108. Guerguerian G, Dahan E. A case of laryngeal tuberculosis with out pulmonary lesions. *Rev Med Moyen Orient* 1952; **9:** 248-249.
109. Johansen J. A case of oral tuberculosis. *Nor Tannlaegeforen Tid* 1952; **62:** 459-462.
110. Karengina MS. Treatment of laryngeal-pulmonary tuberculosis with special reference to nursing. *Med Sestra* 1952**:** 20-23.
111. Lumio JS. Streptomycin therapy of laryngeal tuberculosis. *Duodecim* 1952; **68:** 991-999.
112. Mandi L. Therapy of respiratory tuberculosis in the adults. *Orv Hetil* 1952; **93:** 382-386.
113. Mehmet A. Tuberculosis of the larynx and its treatment. *J Med Liban* 1952; **5:** 576-579.
114. Misiewicz J, Osinska K, Wroczynska K. Treatment of laryngeal and pulmonary tuberculosis with small doses of streptomycin. *Gruzlica* 1952; **20:** 233-238.
115. Motta R, Gammarrota V. Clinical and anatomical considerations on the treatment of tuberculosis of the epiglottis with streptomycin. *Ann Ist Carlo Forlanini* 1952; **13:** 311-325.
116. Motta R, Gammarrota V. Clinical and anatomical cure in the treatment of laryngeal tuberculosis with streptomycin. *Ann Ist Carlo Forlanini* 1952; **13:** 303-310.
117. Motta R, Novak G. Laryngeal polpyi and tuberculosis. *Clin Nuova Rass Prog Med Int* 1952; **15:** 67-80.
118. Nell HM. Therapy of laryngeal tuberculosis with special reference to tyrosolvin. *Tuberkulosearzt* 1952; **6:** 22-25.
119. Pagliarini F. Therapy of laryngeal tuberculosis with reference to streptomycin and chemotherapeutic agents. *Monatsschr Ohrenheilkd Laryngorhinol* 1952; **86:** 8-16.
120. Radner DB, Snider GL. Recurrent laryngeal nerve paralysis as a complication of pulmonary tuberculosis; a report of two cases. *Am Rev Tuberc* 1952; **65:** 93-99.
121. Roald S, Thomassen OK. Dependability of laryngeal cultures for detection of tubercle bacilli. *Nord Med* 1952; **48:** 1619-1620.
122. Rosen DM, Shapiro MJ, Stepanoff N. Isonicotinic acid hydrazine derivatives in the treatment of otolaryngological tuberculosis. *Q Bull Sea View Hosp* 1952; **13:** 197-202.
123. Rozenblat MS. Paralaryngeal method of administration of streptomycin in the treatment of dysphagia in laryngeal tuberculosis. *Probl Tuberk* 1952; **6:** 71-72.
124. Rozenblat MS. Result of the application of tibone in pulmonary and laryngeal tuberculosis. *Vestn Otorinolaringol* 1952; **14:** 79.
125. Sanguigno N. Rapid therapeutic action of isonicotinic acid hydrazide in grave oropharyngo-laryngeal tuberculosis. *Riforma Med* 1952; **66:** 505-507.
126. Sieluzycki C. Laryngeal tuberculosis. *Gruzlica* 1952; **20:** 415-423.
127. Titche LL. Laryngeal manifestations of intrathoracic conditions. *Trans Pac Coast Otoophthalmol Soc Annu Meet* 1952; **33:** 115-131; discussion, 131-115.
128. Vago A. Effect of isonicotinic acid hydrazide in tubercular laryngitis and bronchitis. *Minerva Med* 1952; **43:** 1112-1113.
129. Wallner LJ. Antimicrobial therapy of laryngeal tuberculosis. *Eye Ear Nose Throat Mon* 1952; **31:** 25-29.
130. Zal'Tsman SD. Streptomycin therapy and therapeutic complications in laryngo-pulmonary tuberculosis. *Vestn Otorinolaringol* 1952; **14:** 67-70.
131. Zanzucchi G, Lupacchini A. Several cases of laryngeal tuberculosis resistant to antibiotics, treated with galvanocautery. *G Ital Della Tuberc* 1952; **6:** 19-24.
132. Zlatopol'Skaia ES. Laryngeal tuberculosis in children simulating diphtherial croup. *Pediatriia* 1952; **1:** 72.
133. Abba GC, Perego C. Course of tuberculous meningitis treated with streptomycin and isonicotinic acid hydrazide by spinal, oral and parenteral administration. *Clin Pediatr (Bologna)* 1953; **35:** 551-554.
134. Arold C. Current status of therapy of tuberculosis of mucous membranes. *Z Laryngol Rhinol Otol* 1953; **32:** 141-146.
135. Bertelli JA. Pharyngolaryngeal tuberculosis. *Prensa Med Argent* 1953; **40:** 2597-2609.
136. Boj E. Treatment of laryngeal tuberculosis with small doses of streptomycin. *Gruzlica* 1953; **21:** 47-48.
137. Bour H, Batisse R. Rhinopharyngeal intervention in children in primary tuberculosis. *Ann Otolaryngol* 1953; **70:** 58-67.
138. Calvet J, Coll J, Malhiac H. The late laryngeal aspects and recurrences in tuberculous laryngitis treated with streptomycin. *Rev Tuberc* 1953; **17:** 873-875.
139. Calvet J, Ribet A, Coll J. Isonicotinic acid hydrazide therapy of pseudo-tumoral laryngeal tuberculosis. *Rev Tuberc* 1953; **17:** 365-366.
140. Castigliano SG, Shigeoka E. Tuberculosis of the oral cavity. *Ann Otol Rhinol Laryngol* 1953; **62:** 662-676.
141. Collins DH, Shucksmith HS. Tuberculosis of Parotid Adenolymphoma and of Lymph Glands Incorporating Salivary Ducts. *Journal of Pathology and Bacteriology* 1953; **66:** 399-&.
142. De Castro L. Inhalation of very fine conteben powder in laryngeal and pulmonary tuberculosis. *Rev Esp Tuberc* 1953; **22:** 499-504.
143. Gondel'Man-Binshtok IS. Treatment of residual speech disorders following clinical cure of laryngeal tuberculosis. *Vestn Otorinolaringol* 1953; **15:** 22-25.
144. Heuck F. Significance of the stratigraphic examination for the early diagnosis of laryngeal tuberculosis. *Beitr Klin Tuberk Spezif Tuberkuloseforsch* 1953; **110:** 321-328.
145. Kroath F. Symptom remission and recovery in laryngeal tuberculosis after conteben therapy. *Monatsschr Ohrenheilkd Laryngorhinol* 1953; **87:** 117-122.
146. Lehmann RR. Tuberculosis of the larynx and its treatment with streptomycin and hydrazine derivatives of isonicotinic acid. *Laryngoscope* 1953; **63:** 977-990.
147. Link R. Neoteben therapy of laryngeal tuberculosis. *Z Laryngol Rhinol Otol* 1953; **32:** 146-150.
148. Motta R, Antonelli G. TB I/698 in laryngopulmonary tuberculosis. *Clin Otorinolaringoiatr* 1953; **5:** 237-247.
149. Motta R, Cutillo E, Toussan U. Treatment of pharyngo-laryngo-pulmonary tuberculosis with isonicotinic acid hydrazide. *Clin Nuova Rass Prog Med Int* 1953; **16:** 309-312.
150. Ovedoff DL, Bensusan AD. ACTH and cortisone in the treatment of pulmonary laryngeal tuberculosis. *S Afr Med J* 1953; **27:** 613-615.
151. Podgaetskii GB. Latent tuberculosis of the pharyngeal and palatine tonsils in children and adolescents. *Probl Tuberk* 1953; **6:** 20-25.
152. Radenbach KL, Link R, Niedermowe W. Therapy of tuberculosis of upper respiratory tract, especially, laryngeal tuberculosis with isonicotinic acid hydrazide. *Beitr Klin Tuberk Spezif Tuberkuloseforsch* 1953; **109:** 449-466.
153. Schwarzbart AA. Tuberculosis of the larynx. *Harofe Haivri Heb Med J* 1953; **2:** 184-187.
154. Sieluzycki C. Laryngeal tuberculosis in sanatoria. *Gruzlica* 1953; **21:** 447-452.
155. Spesivtsev OG. Effects of streptomycin therapy of laryngeal tuberculosis. *Vestn Otorinolaringol* 1953; **15:** 78.
156. Vallesi RN. Incidence of tuberculosis in laryngeal cancer. *Boll Mal Orecch Gola Naso* 1953; **71:** 146-163.
157. Varas O, Munoz W. Pneumolaryngeal tuberculosis and pregnancy. *Bol Soc Chil Obstet Ginecol* 1953; **18:** 28-31.
158. Zanzucchi G. Remote results of streptomycin therapy of laryngeal tuberculosis. *G Ital Della Tuberc* 1953; **7:** 210-214.
159. Zitka E. Tuberculosis of the oral cavity and its surroundings. *Klin Med Osterr Z Wiss Prakt Med* 1953; **8:** 33-40.
160. Chumakov FI. [Clinical and morphological characteristics of laryngeal tuberculosis treated with streptomycin and PAS.] *Probl Tuberk* 1954; **4:** 29-35. Russian.
161. Corcos A, Perez A. [Isambert's disease (pharyngeal tuberculosis) cured rapidly by antibiotics.] *Tunis Med* 1954; **42:** 883-885. French.
162. D'Olev L. [Roentgenotherapy of laryngeal tuberculosis.] *Suvr Med (Sofiia)* 1954; **5:** 30-34. Bulgarian.
163. De Michelis B. [Antibiotics in the treatment of oral tuberculosis.] *Minerva Stomatol* 1954; **3:** 119-123. Italian.
164. Despons J. [Paralysis of the dilator muscles of the larynx after cure of tuberculous laryngitis by streptomycin.] *Rev Otoneuroophtalmol* 1954; **26:** 168-170. French.
165. Eder J. [Intravenous administration of novocain in control of cough in pulmonary and laryngeal tuberculosis.] *Pol Tyg Lek (Wars)* 1954; **9:** 1328-1330. Polish.
166. Gandon J. Laryngeal tuberculosis and rimifon. *Ann Otolaryngol* 1954; **71:** 96-98.
167. Glacometti GS. Tuberculous laryngitis and pulmonary tuberculosis: incidence and treatment. *Friuli Med* 1954; **9:** 107-112.
168. Jimenez Andrades D. [Laryngeal tuberculosis today.] *Med Cir Guerra* 1954; **16:** 173-182. Spanish.
169. Lapina AA. [Problem of recurrence of laryngeal tuberculosis treated with streptomycin.] *Probl Tuberk* 1954; **4:** 24-29. Russian.
170. Lell WA. Laryngeal tuberculosis: diagnosis, incidence, and present-day treatment. *AMA Arch Otolaryngol* 1954; **60:** 350-366.
171. Lepoivre M. [Primary infection in tuberculosis of the oral mucosa.] *Rev Prat* 1954; **4:** 2221-2228. French.
172. Montis F. [Laryngeal tuberculosis and hydrazide.] *Medicina (Madr)* 1954; **22:** 138-145. Spanish.
173. Natanzon AM. [Surgical intervention in laryngeal cancer in tuberculosis.] *Vestn Otorinolaringol* 1954; **16:** 50-54. Russian.
174. Nezlin SE. [Incidence of tuberculosis of the intestines and of the larynx (according to the autopsy findings)]. *Klin Med (Mosk)* 1954; **32:** 25-28. Russian.
175. Ormerod FC. Tuberculosis of the upper air passages and ears. *Br J Tuberc Dis Chest* 1954; **48:** 214-216.
176. Prietzel F. [Combined tuberculostatic therapy of laryngopulmonary tuberculosis.] *Wien Med Wochenschr* 1954; **104:** 1013-1020. German.
177. Suehs OW. Tuberculosis in review. III. Bronchoscopy in tuberculosis and recognition and management of tuberculous laryngitis. *Tex State J Med* 1954; **50:** 335.
178. Balogh K. [Oral tuberculosis.] *Tuberk Kerdesei* 1955; **8:** 9-13. Hungarian.
179. Belfort F. [The oto-rhino-laryngology tuberculosis. VI. Laryngology tuberculosis.] *Rev Bras Otorrinolaringol* 1955; **23:** 117-139; contd. Portuguese.
180. Bochenek A. [Tuberculosis of the laryngs and lungs.] *Pol Tyg Lek (Wars)* 1955; **10:** 297-300. Polish
181. Cerevellera G, Bellomo D. [Case of laryngeal tuberculosis and luetic neurolabyrinthitis.] *Boll Mal Orecch Gola Naso* 1955; **73:** 43-47. Italian.
182. Jimenez Encina C. [History of laryngeal tuberculosis.] *Bol Cult Inf Cons Gen Col Med Esp* 1955; **18:** 61-63. Spanish.
183. Klott M, Kuczborski S. [Therapy of tuberculous of oral cavity, pharynx and larynx with isonicotinic acid hydrazide.] *Gruzlica* 1955; **23:** 409-416. Polish.
184. Kmita S, Kolbow H. [Clinical observations on action of isonicotinic acid hydrazide in laryngeal tuberculosis.] *Otolaryngol Pol* 1955; **9:** 227-232. Polish.
185. Pelaez JF. [Osteogenesis imperfecta; a case of blue sclera with tuberculous lesions of the lung and larynx.] *Rev Cubana Pediatr* 1955; **27:** 369-376. Spanish.
186. Piasecki W. [Observations on the treatment of laryngeal tuberculosis with isonicotinic acid hydrazide.] *Gruzlica* 1955; **23:** 711-716. Polish.
187. Piechaud F, Abadie C. [Evolution of laryngo-pulmonary tuberculosis in our days.] *J Med Bord* 1955; **132:** 245-253. French.
188. Stachowsky L. [Current status of therapy of tuberculosis of the mucous membranes of the ENT area, a comparative evaluation of new drugs, streptomycin, PAS, TB1 and isonicotinic acid hydrazide.] *Gac Med Caracas* 1955; **63:** 326-328. French.
189. Steffl M. [Tuberculosis of the pharyngeal tonsil in adults.] *Vnitr Lek* 1955; **1:** 54-58. Czech.
190. Toussaint-Francx Y, Toussaint P. [Bronchial tuberculosis and the broncho-tracheo-laryngeal tract of infection; comparative examination of the autopsy resected pieces in autopsy & surgery.] *Acta Tuberc Belg* 1955; **46:** 261-284. French.
191. Tsukioka K. [A pathological study of laryngeal tuberculosis applying the fluorescence microscope (Yasaki). II.] *Kekkaku* 1955; **30:** 359-364; 401-352. Japanese.
192. Tsukioka K. [Pathological study of laryngeal tuberculosis applying the fluorescent microscope (Yasaki). I.] *Kekkaku* 1955; **30:** 291-297; English summary, 338. Japanese.
193. Wolf HE. [New gains in therapy of laryngeal and pulmonary tuberculosis by use of combionta, a Merck preparation.] *Z Laryngol Rhinol Otol* 1955; **34:** 812-817. German.
194. Arnold LM. Management of tuberculosis of the larynx. *J Mt Sinai Hosp N Y* 1956; **23:** 616-620.
195. Arold C. [Chemotherapy in tuberculosis of the upper respiratory tract.] *Ergeb Gesamten Tuberkuloseforsch* 1956; **13:** 437-455. German.
196. Brandao H. [Pulmonary tuberculosis and tuberculous laryngitis: 587 cases.] *Rev Assoc Med Bras* 1956; **2:** 254-261. Portuguese.
197. Ivashchenko EA. [Immediate results of larusan therapy in tuberculosis of the upper respiratory tract and oral cavity.] *Probl Tuberk* 1956; **34:** 18-19. Russian.
198. Konecny Z. [Tuberculous bacilli in the upper respiratory tract.] *Cesk Otolaryngol* 1956; **5:** 39-43. Czech.
199. Portmann M, Abadie C. [Laryngeal tuberculosis in the last thirty years.] *Bord Chir* 1956**:** 11-14. French.
200. Tanzi PL. [Present position of the laryngeal localization in the morbid cycles of tuberculosis.] *Arch Tisiol Mal Appar Respir* 1956; **11:** 58-76. Italian.
201. Torzecka W, Torzecki Z. [Laryngeal tuberculosis in autopsy material.] *Otolaryngol Pol* 1956; **10:** 71-75. Polish.
202. Bombardelli E. [Clinical aspects of a case of suspected primary tuberculosis of the oral cavity.] *Minerva Med* 1957; **48:** 1787-1792. Italian.
203. Chumakov FI. [Cure in tuberculosis and cancer of the larynx.] *Vestn Otorinolaringol* 1957; **19:** 102-104. Russian.
204. Chumakov FI. [Some peculiarities in the process of laryngeal tuberculosis in patients treated with streptomycin and chemical preparations.] *Probl Tuberk* 1957; **35:** 37-45. Russian.
205. Ewert EG, Meixner HJ. [Chemotherapy of laryngeal tuberculosis.] *Dtsch Gesundheitsw* 1957; **12:** 545-550. German.
206. Grimaud R, Vesselle P, Werner J. [Tuberculosis of the laryngeal sinus.] *Ann Otolaryngol* 1957; **74:** 343-345. French.
207. Piatti A, Pasini C. [Pseudo-neoplastic aspects of laryngeal tuberculosis.] *Arch Ital Otol Rinol Laringol* 1957; **68:** 531-543. Italian.
208. Wallner LJ. Further experience with antimicrobial therapy of laryngeal tuberculosis. *Eye Ear Nose Throat Mon* 1957; **36:** 397-400.
209. Wallner LJ. Tuberculosis of the larynx. *Am J Med Sci* 1957; **233:** 448-455.
210. Agroskin CI, Davydovich EP. [Tuberculosis of the upper respiratory tract in children.] *Pediatriia* 1958; **41:** 43-48. Russian.
211. Christensen J. [Tracheotomy in acute cases of laryngeal tuberculosis.] *Ugeskr Laeger* 1958; **120:** 317-319. Danish.
212. Chumakov FI. [Clinical and patho-morphological characteristics of laryngeal tuberculosis treated with streptomycin, PAS and phthivasid.] *Vestn Otorinolaringol* 1958; **20:** 77-82. Russian.
213. Herrmann A. [Modern diagnosis & therapy of laryngeal tuberculosis.] *Med Monatsschr* 1958; **12:** 385-390. German.
214. Mounier K, Piaget F, Gaillard J. [Several present-day aspects of pharyngo-laryngeal tuberculosis & the association of cancer & tuberculosis.] *J Med Lyon* 1958; **39:** 951-960. French.
215. Nozzoli F, Pazzagli G. [Nasal & pharyngeal miliary tuberculosis simulating lupus vulgaris; clinical observations.] *Boll Mal Orecch Gola Naso* 1958; **76:** 640-652. Italian.
216. Aliperta A, Senis F. [Current aspects of the reparative processes of tubercular lesions of the larynx.] *Arch Tisiol Mal Appar Respir* 1959; **14:** 662-682. Italian.
217. Cohen L. Oral tuberculosis. *Oral Surg Oral Med Oral Pathol* 1959; **12:** 430-437.
218. Gabka E, Harnisch H. [Tuberculous mucous membrane affections of the oral cavity.] *Z Haut Geschlechtskr* 1959; **27:** 180-184. German.
219. Hascouet M. [Laryngeal tuberculosis of papillomatous form cured by the association of cortancyl and antitubercular drugs.] *Ann Otolaryngol* 1959; **76:** 820-822. French.
220. Shaw HJ, Friedmann I. Bilateral Adenolymphoma of the Parotid Salivary Gland Associated with Tuberculosis. *British Journal of Surgery* 1959; **46:** 500-505.
221. Young RV. Tuberculous laryngo-tracheobronchitis: bronchoscopic evaluation. *Arch Med Panamenos* 1959; **8:** 35-50.
222. Arold C, Kaempfer R. [Follow-up of laryngeal tuberculosis treated with tuberculostatics.] *Tuberkulosearzt* 1960; **14:** 844-849. German.
223. Charlet R, Mereau J, Deltour C. [Ganglio-pharyngeal tuberculosis associated with multifocal cutaneous tuberculosis.] *Lille Med* 1960; **5:** 474-475. French.
224. Cook HP. Oral tuberculous ulceration. *Proc R Soc Med* 1960; **53:** 473-474.
225. Fernandez J. [Histopathology of the nerve endings in tuberculous laryngitis.] *Arq Neuropsiquiatr* 1960; **18:** 133-151. Portuguese.
226. Finzi A, Barbareschi G. [Pharyngeal tuberculosis. (Clinical and histopathological observations on 2 cases of tuberculosis of ulcerovegetans type.] *Arch Ital Otol Rinol Laringol* 1960; **71:** 654-668. Italian.
227. Rosemberg J, de CN, Aun JN, Passos Filho MC. Immunobiologic relation between tuberculosis and leprosy. X. Comparative study of the results of the lepromin test in subjects submitted to serial injections of Mitsuda's antigen and to oral BCG vaccination. *Int J Lepr* 1960; **28:** 271-283.
228. Tessier R, Berger M, Martre P, Chomy P, Boisseau MM. [Recrudescence of laryngitis in tuberculotics.] *J Med Bord* 1960; **137:** 278-284. French.
229. Zeman K. [Diagnosis and modern therapy of laryngeal tuberculosis. (Experiences with the diagnosis and therapy of 160 patients in the clinic of Academician Precechtel).] *Cas Lek Cesk* 1960; **99:** 1596-1603. Czech.
230. Gardner JA, Hanft RJ. A tuberculous granuloma of the oral mucosa and cervical lymph nodes. Report of a case. *Oral Surg Oral Med Oral Pathol* 1961; **14:** 406-413.
231. Kovacs K, Szalay G. [Laryngeal tuberculosis and pregnancy.] *Orv Hetil* 1961; **102:** 1172-1173. Hungarian.
232. Lowys P, Le Barre H, Roux J, Faivre A, Boye J. [2 cases of pharyngeal tuberculosis appearing in children during sanatorial treatment.] *Riv Chir Pediatr* 1961; **25:** 389-397. French.
233. Paul MB, Bowden AC. Two cases of pulmonary tuberculosis presenting with oral lesions. *Br J Dis Chest* 1961; **55:** 41-42.
234. Cappellano R. [Cancer and tuberculosis of the upper respiratory and digestive tracts. Association and differential diagnosis.] *Rev Bras Cir* 1962; **44:** 149-153. Portuguese.
235. Chomkoviczova T. [Cancer of the larynx and tuberculosis.] *Cesk Otolaryngol* 1962; **11:** 229-235. Czech.
236. Mathur JB, Chaube CK. Laryngeal involvement in pulmonary tuberculosis. *J Indian Med Assoc* 1962; **39:** 453-459.
237. Sasaki H. [Case of laryngeal involvement in spinal caries.] *Jibiinkoka* 1962; **34:** 765-768. Japanese.
238. Baruffi W, Fadel JC, Koga K, Montenegro W. [Considerations on the use of liquid hydrazide in aerosol form as the sole medication and as an adjuvant to 1st-line tuberculostatic agents by oral route, in the treatment of pulmonary and laryngeal tuverculosis. Experience with 31 cases.] *Rev Bras Tuberc Doencas Torac* 1963; **31:** 235-250. Portuguese.
239. Bell WJ, Brown PP. Oral out-patient chemotherapy for pulmonary tuberculosis in west Africans. *West Afr Med J* 1963; **12:** 267-274.
240. Diepeveen J, Welin F. ["Laryngeal tumor" and laryngeal tuberculosis.] *Ugeskr Laeger* 1963; **125:** 987-989. Danish.
241. Novruzov MA. [Primary tuberculosis of the tonsils.] *Probl Tuberk* 1963; **41:** 88-90. Russian.
242. Parolari P, Piatti A, Zerba L. [Laryngeal tuberculosis of pseudo-tumoral appearance.] *Arch Ital Otol Rinol Laringol* 1963; **74:** 881-898. Italian.
243. Akaike K, Otsuka K, Watanabe K, Nagano Y, Hoshino M. [On a rare case of tuberculosis in the sinus and nasopharynx.] *Jibiinkoka* 1964; **36:** 953-956. Japanese.
244. Brzezinska H, Golebiowska M. [A case of tuberculosis of the nose, oral cavity, larynx and pahrynx in a 3-year-old child.] *Pol Tyg Lek* 1964; **19:** 183-184. Polish.
245. Grande F. [Consideration on combined tuberculosis and cancer of the larynx. (Clinical contribution).] *Arch Tisiol Mal Appar Respir* 1964; **19:** 1013-1029. Italian.
246. Konecny Z, Fajkosova D. [Plastic surgery of the larynx in specific obliteration.] *Cesk Otolaryngol* 1964; **13:** 156-160. Czech.
247. Lagerloef B, Wersaell J. Tuberculous lesions of the oral mucosa. *Oral Surg Oral Med Oral Pathol* 1964; **17:** 735-738.
248. Lecoulant P, Texier L, Tessier R, Martre P, Maleville J, Bretelle J. [Buccal laryngeal and pulmonary tuberculosis]. *Toulouse Med* 1964; **65:** 73-77. French.
249. Taillens JP. [Tonsillar and lymph node tuberculosis. Frequent diagnosis, if the laboratory is judged by pathological and clinical criteria.] *Pract Otorhinolaryngol (Basel)* 1964; **26:** 126-144. French.
250. Ameli M, Dorigoni A. [Pharyngeal tuberculosis (clinical and histopathologic observations on a case of tubercular ulcero-vegetans pharyngitis).] *Arch Ital Laringol* 1965; **73:** 129-141. Italian.
251. Amsler R, Fresnau, Salquain C, Grimault Y. [Tuberculous laryngitis, an initial sign of pulmonary tuberculosis.] *Concours Med* 1965; **87:** 1957-1968. French.
252. Licata G. [Tuberculosis of the oral cavity with particular reference to a rare case of personal observation (palatal ulceration with perforation of the hard palate).] *Stomatologica (Genova)* 1965; **9:** Suppl:487-501. Italian.
253. Werelds RJ. [The possible role of the tooth in oral tuberculosis.] *Acta Stomatol Belg* 1965; **62:** 361-373. Dutch.
254. Debain JJ. [Some current aspects of laryngeal tuberculosis.] *Ann Otolaryngol Chir Cervicofac* 1966; **83:** 489-492. French.
255. Pellegrini G, Orsini P, Boyre F, Philip F. [Bucco-pharyngeal tuberculous primary infection (apropos of 2 cases).] *J Med Bord* 1966; **143:** 1907-1914. French.
256. Rzeszutko R, Wieczorek Z. [Evaluation of usefulness of hemagglutination reaction in the diagnosis of tuberculosis of the oral cavity and the surrounding lymphatic glands.] *Czas Stomatol* 1966; **19:** 879-884. Polish.
257. Sosnowski K. [Tuberculosis of the oral cavity.] *Wiad Lek* 1966; **19:** 961-963. Polish.
258. Zanotelli F, Oreglia P, Cavaliere R. [Contribution to the study of oral tuberculosis.] *Fracastoro* 1966; **59:** 242-252. Italian.
259. Bjorlin G. Oral tuberculosis. *Odontol Revy* 1967; **18:** 395-399.
260. Daddi G, Cornia G, Grassi C, Perna G, Scarpazza G. [1st observations on the activity of the oral Rifampicin in the treatment of chronic pulmonary tuberculosis.] *G Ital Mal Torace* 1967; **21:** 131-136. Italian.
261. Gaia F, Orlandoni A. [Current clinical aspects of laryngeal tuberculosis.] *Boll Mal Orecch Gola Naso* 1967; **84:** 460-470. Italian.
262. Gambetti G, Mulargia A. [In the pathogenesis of tuberculosis of the oral cavity. (Experimental report).] *Stomatologica (Genova)* 1967; **12:** 37-67. Italian.
263. Jirousek Z. [Observations on the tuberculous inflammations of lymph nodes in the orofacial region.] *Cesk Stomatol* 1967; **67:** 366-369. Czech.
264. Liubomirova IM, Barysheva Iu D. [A case of simultaneous cancer, tuberculosis and candidiasis of the oral mucosa.] *Stomatologiia (Mosk)* 1967; **46:** 103-104. Russian.
265. Merlini C, Fabri N. [Tuberculous infection and the oral cavity.] *Rass Trimest Odontoiatr* 1967; **48:** 455-482. Italian.
266. Nicolin LT. [On a case of double localization of tuberculosis in the oral cavity.] *Ann Stomatol (Roma)* 1967; **16:** 997-1006. Italian.
267. Obreja S, Tanase T. [Considerations on some diagnostic problems in laryngeal tuberculosis.] *Otorinolaringologie* 1967; **12:** 123-127. Romanian.
268. Struzak-Wysokinska M. [Diagnosis and treatment of primary tuberculosis of oral mucosa.] *Czas Stomatol* 1967; **20:** 47-50. Polish.
269. Wayoff M, Martin J. [Our experience with laryngeal tuberculosis from 1955 to 1965.] *J Fr Otorhinolaryngol Audiophonol Chir Maxillofac* 1967; **16:** 485-487. French.
270. De Mitri T. [Present anatomo-clinical evolution of pharyngo-laryngeal tuberculosis (considerations on 10 cases).] *Ann Laringol Otol Rinol Faringol* 1968; **67:** 17-35. Italian.
271. Delaire J, Billet J. [Primary tuberculous infection of the oral mucosa.] *Actual Odontostomatol (Paris)* 1968; **22:** 273-340. French.
272. Dosen D, Prvanov I. [Coincidence of pulmonary tuberculosis with laryngeal tuberculosis.] *Tuberkuloza* 1968; **20:** 106-109. Croatian.
273. Koseva S. [Laryngeal and pulmonary tuberculosis in the era of antitubercular agents.] *God Zb Med Fak Skopje* 1969; **15:** 341-346. Serbian.
274. Popovic J, Praso R. [Haracteristics of pulmonary tuberculosis in patients with laryngeal tuberculosis.] *Plucne Bolesti Tuberk* 1969; **21:** 358-363. Croatian.
275. Schmid F. [Management of the oral cavity in tuberculosis.] *Prax Pneumol* 1969; **23:** 844-847. German.
276. Sitbon J. [Pharyngeal cavum tuberculosis with bilateral cervical adenopathy simulating cancer.] *J Fr Otorhinolaryngol Audiophonol Chir Maxillofac* 1969; **18:** 805-806. French.
277. Brennan TF, Vrabec DP. Tuberculosis of the oral mucosa. Report of a case. *Ann Otol Rhinol Laryngol* 1970; **79:** 601-605.
278. Cajsfinger H, Gadot P, Gignoux B. [A clinical case of pharyngo-laryngeal tuberculosis without lung involvement.] *J Fr Otorhinolaryngol Audiophonol Chir Maxillofac* 1970; **19:** 664-665. French.
279. Mikulska W. [Laryngeal tuberculosis in the course of modern treatment of pulmonary tuberculosis.] *Otolaryngol Pol* 1970; **24:** 687-691. Polish.
280. Muroff FI. Tuberculous lesion of the oral cavity: report of case. *J Can Dent Assoc (Tor)* 1970; **36:** 380-381.
281. Mysakowska H, Wlodarski B, Sidor-Smaga M, Grodzki S. [Laryngeal and oral tuberculosis in patients with pulmonary tuberculosis.] *Pol Tyg Lek* 1970; **25:** 485-487. Polish.
282. Nedwicki EG. Laryngeal tuberculosis simulating carcinoma of the larynx. *Med Times* 1970; **98:** 181-187.
283. Pellant A. [Laryngeal tuberculosis following partial frontolateral laryngectomy.] *Cesk Otolaryngol* 1970; **19:** 141-142. Polish.
284. Stanek S. [Results of antituberculous treatment of laryngeal tuberculosis with reference to substitute drugs.] *Otolaryngol Pol* 1970; **24:** 681-686. Polish.
285. Barysheva Iu D, Popova Iu N. [The diagnosis of ulcerous tuberculosis of the oral mucous membrane.] *Stomatologiia (Mosk)* 1971; **50:** 74-76. Russian.
286. Buruiana M. [Current aspects of laryngeal tuberculosis. Correlation of laryngeal lesions with pulmonary and bronchial tuberculosis.] *Ftiziologia* 1971; **20:** 629-634. Romanian.
287. Buruiana M, Stinge V. [Bronchial chondroma in a case of active pulmonary and laryngeal tuberculosis and endopleural fibrothorax following therapeutic pneumothorax. Pathogenic elements.] *Ftiziologia* 1971; **20:** 533-537. Romanian.
288. Cleaton-Jones P. Oral tuberculosis--its similarity to oral carcinoma. *J Can Dent Assoc (Tor)* 1971; **37:** 388-389.
289. Das AK. Tuberculosis of the oral mucosa. *J Indian Dent Assoc* 1971; **43:** 79-84.
290. Maran AG, Stewart IA. Laryngeal tuberculosis. *Br Med J* 1971; **2:** 775.
291. Mangiulea V, Rudescu D. [On treatment with ethambutol administered in the form of aerosols or endobronchial instillations in laryngeal and bronchopulmonary tuberculosis.] *Ftiziologia* 1972; **21:** 603-608. Romanian.
292. Nanjo S, Suga A, Bono K, Osawa H, Yamaguchi Y. [3 cases suspected to be laryngeal tuberculosis.] *Jibiinkoka* 1972; **44:** 227-231. Japanese.
293. de Almeida ES, Barbieri T, Gomes M, Guedes E, Soares L. [Tuberculosis infection and the use of oral and intradermal BCG in school children of Laranjal Paulista, Sao Paulo, Brazil.] *Rev Saude Publica* 1973; **7:** 189-197. Portuguese.
294. Gaillard de Collogny L, Molina C, Lafaye M, Brun J. [Pseudotumoral advanced laryngeal tuberculosis revealing pulmonary tuberculosis.] *JFORL J Fr Otorhinolaryngol Audiophonol Chir Maxillofac* 1973; **22:** 45-50. French.
295. Gastpar H. [Treatment of laryngeal tuberculosis (author's transl).] *Z Laryngol Rhinol Otol* 1973; **52:** 604-608. German.
296. Harris BC, Taylor CG, Wade WW, Jr. Miliary tuberculosis with oral manifestations: report of case. *J Oral Surg* 1973; **31:** 305-307.
297. Laws IM. Oral tuberculosis. Case reports. *Br Dent J* 1973; **134:** 146-148.
298. Ramulu C, Prahlad D, Reddy CR. Tuberculous ulcer of the oral cavity--a case report. *J Indian Dent Assoc* 1973; **45:** 251-253.
299. Stefanovic P, Ilic C, Stanisavljevic B, Djordjevic M. [Current views on laryngeal tuberculosis.] *Srp Arh Celok Lek* 1973; **101:** 163-167. Serbian.
300. Buruiana M, Mindru R. [2 cases of extensive pharyngo-laryngeal tuberculosis.] *Rozhl Chir* 1974; **53:** 227-231. Romanian.
301. Morse JO. Letter: Laryngeal tuberculosis or tumor. *Ann Intern Med* 1974; **81:** 711-712.
302. Brodovsky DM. Laryngeal tuberculosis in an age of chemotherapy. *Can J Otolaryngol* 1975; **4:** 168-176.
303. Campos Rivera M, Gutierrez Aldana G. [Oral tuberculosis (report of 2 cases).] *Med Cutan Ibero Lat Am* 1975; **3:** 299-302. Spanish.
304. Sidhu SS, Subherwal GL, Kapoor U, Parkash H. Oral tuberculosis. *Newsl Int Coll Dent India Sect* 1975; **12:** 7, 9.
305. Yusuf H. Oral tuberculosis. Two case reports. *Br Dent J* 1975; **138:** 470-472.
306. Alavoine J, Debarge A, Mathieu F. [A case of laryngeal tuberculosis.] *Rev Laryngol Otol Rhinol (Bord)* 1976; **97:** 231-236. French.
307. Andreevski A, Petrovski R, Caparevski S, Filipce I. [Pseudotumorous form of laryngeal tuberculosis as non-diagnosed cases of evolutive pulmonary tuberculosis.] *God Zb Med Fak Skopje* 1976; **22:** 435-438. Serbian.
308. Horowitz G, Kaslow R, Friedland G. Infectiousness of laryngeal tuberculosis. *Am Rev Respir Dis* 1976; **114:** 241-244.
309. Laws I. Letter: Oral lesions in tuberculosis. *Br Med J* 1976; **2:** 45-46.
310. McAndrew PG, Adekeye EO, Ajdukiewicz AB. Miliary tuberculosis presenting with multifocal oral lesions. *Br Med J* 1976; **1:** 1320.
311. Naraqi S, Raiser MW, Richards NM, Andersen BR. Tuberculosis of the larynx masquerading as carcinoma. *Ann Otol Rhinol Laryngol* 1976; **85:** 547-548.
312. Sang G, Dusanee P. [Oral tuberculosis. Case report.] *J Dent Assoc Thai* 1976; **26:** 189-199. Thai.
313. Travis LW, Hybels RL, Newman MH. Tuberculosis of the larynx. *Laryngoscope* 1976; **86:** 549-558.
314. Belen'kii MS, Kitaevich AE. [Certain characteristics of present-day laryngeal tuberculosis.] *Zh Ushn Nos Gorl Bolezn* 1977**:** 98-100. Russian.
315. Calatrava L. [Oral tuberculosis. Disease of Isambert.] *Bol Inf Dent (Madr)* 1977; **37:** 33-39. Spanish.
316. Claux J, Ane P, Chamayou P. [A case report of laryngeal tuberculosis with pseudotumoral appearance.] *J Fr Otorhinolaryngol Audiophonol Chir Maxillofac* 1977; **26:** 387-390. French.
317. Giardino C, Lavorgna G. [Oral cavity tuberculosis. (Considerations on three cases).] *Arch Stomatol (Napoli)* 1977; **18:** 3-14. Italian.
318. Grudzinska J, Spychalski L, Saldziun D, Kustosz D. [Oral mucosa tuberculosis involving facial bones and submandibular and cervical lymph nodes.] *Czas Stomatol* 1977; **30:** 346. Polish.
319. Lindell MM, Jr., Jing BS, Wallace S. Laryngeal tuberculosis. *AJR Am J Roentgenol* 1977; **129:** 677-680.
320. Tada S, Kino M, Yamamoto W, Harada J, Kanehira C. Contrast nasopharyngography. *Clin Radiol* 1977; **28:** 659-662.
321. de Padua Dias MH, Hayashi A. [Tuberculin test, oral BCG and tuberculosis infection in children under 5 years old.] *Rev Saude Publica* 1978; **12:** 443-454. Portuguese.
322. Krut'ko VS, Stadnikova AV, Shevchenko VV, Kuzin LP. [Diagnosis of laryngeal tuberculosis in a polyclinic.] *Probl Tuberk* 1978**:** 50-52. Russian.
323. Miller RL, Krutchkoff DJ, Giammara BS. Human lingual tuberculosis. An ultrastructural study. *Arch Pathol Lab Med* 1978; **102:** 360-365.
324. Prabhu SR, Daftary DK, Dholakia HM. Tuberculous Ulcer of Tongue - Report of Case. *Journal of Oral Surgery* 1978; **36:** 384-386.
325. Rauch DM, Friedman E. Systemic tuberculosis initially seen as an oral ulceration: report of case. *J Oral Surg* 1978; **36:** 387-389.
326. Rebattu JP, Perrin LF, Boulud B, Lille R, Colin B. [Tumorous form of laryngeal tuberculosis.] *J Fr Otorhinolaryngol Audiophonol Chir Maxillofac* 1978; **27:** 652-656. French.
327. Thun-Szretter K, Piekarczyk J, Pykalo R. [Tuberculosis of the oral mucosa.] *Czas Stomatol* 1978; **31:** 285-289. Polish.
328. Tyldesley WH. Oral tuberculosis--an unusual presentation. *Br Med J* 1978; **2:** 928.
329. Agarwal MK, Gupta OP, Samant HC, Gupta S, Rastogi BL. Tuberculosis of the tongue. *Ann Acad Med Singapore* 1979; **8:** 217-219.
330. Fujibayashi T, Takahashi Y, Yoneda T, Tagami Y, Kusama M. Tuberculosis of the tongue. A case report with immunologic study. *Oral Surg Oral Med Oral Pathol* 1979; **47:** 427-435.
331. Jones CE, Jones BD. Laryngeal tuberculosis: a case report. *J Natl Med Assoc* 1979; **71:** 37-38.
332. Ogii NV. [Reasons for the late diagnosis of tuberculosis of the larynx, pharynx and oral cavity.] *Zh Ushn Nos Gorl Bolezn* 1979**:** 41-44. Russian.
333. Diktaban T, Lucente FE. Laryngeal tuberculosis: a hazard to the otolaryngologist. *Ear Nose Throat J* 1980; **59:** 488-494.
334. Emery P. Tuberculous abscess of the thyroid with recurrent laryngeal nerve palsy: case report and review of the literature. *J Laryngol Otol* 1980; **94:** 553-558.
335. Gehanno P, Veber F, Guedon C *et al.* [Five cases of pharyngeal tuberculosis seen over a period of one year (author's transl).] *Ann Otolaryngol Chir Cervicofac* 1980; **97:** 923-928. French.
336. Lecointre F, Marandas P, Micheau C, Lacombe H, Schwaab G, Cachin Y. [Tuberculosis of the mucosa of the naso-pharynx. A clinical study of 37 cases seen at the Gustave-Roussy Institute between 1961 and 1978 (author's transl)]. *Ann Otolaryngol Chir Cervicofac* 1980; **97:** 423-433.
337. Ogii NV. [Tuberculosis of the larynx, pharynx and oral cavity with modern chemotherapy.] *Probl Tuberk* 1980**:** 34-37. Russian.
338. Viva E, Parabita GF, Gazzotti A, Sesenna E. [Tuberculosis of the oral mucosa.] *Minerva Stomatol* 1980; **29:** 203-207. Russian.
339. Desiate A, Milano V, Laforgia PD. [Primary tuberculosis of the oral cavity.] *Riv Ital Stomatol* 1981; **50:** 475-486. Italian.
340. Espinoza CG, Montano P, Saba SR. Laryngeal tuberculosis. *Laryngoscope* 1981; **91:** 110-113.
341. Holst E, Pedersen SS. [A case report of tuberculosis with oral manifestations.] *Tandlaegebladet* 1981; **85:** 250-252. Danish.
342. Hunter AM, Millar JW, Wightman AJ, Horne NW. The changing pattern of laryngeal tuberculosis. *J Laryngol Otol* 1981; **95:** 393-398.
343. Niijima K, Sakurai T, Hareyama M *et al.* [Laryngeal tuberculosis which were misdiagnosed as laryngeal cancer: a report of three cases (author's transl).] *Nihon Igaku Hoshasen Gakkai Zasshi* 1981; **41:** 374-379. Japanese.
344. Walsh TJ, Mulholland JH. Laryngeal and perianal tuberculosis simulating laryngeal and perianal carcinoma. *Johns Hopkins Med J* 1981; **149:** 135-137.
345. Yarnal JR, Golish JA, van der Kuyp F. Laryngeal tuberculosis presenting as carcinoma. *Arch Otolaryngol* 1981; **107:** 503-505.
346. Frolova RP. [Treatment of pulmonary and laryngeal tuberculosis by ultrasonic inhalations.] *Probl Tuberk* 1982**:** 69-70. Russian.
347. Manni JJ. The prevalence of tuberculous laryngitis in pulmonary tuberculosis in Tanzanians. *Trop Geogr Med* 1982; **34:** 159-162.
348. Rubin AM, Brondbo K, Alberti PW *et al.* Head and neck manifestations of mycobacteria in the absence of pulmonary disease. *J Otolaryngol* 1982; **11:** 385-390.
349. Araki S, Kuratomi K. Tuberculosis of the larynx. A 10-year review of 14 patients. *Auris Nasus Larynx* 1983; **10 Suppl:** S91-95.
350. Ellis ME, Dunbar EM, Hussain M. Paediatric laryngeal tuberculosis. *Tubercle* 1983; **64:** 37-39.
351. Ibarra A, Arellano L. [Laryngeal tuberculosis: experience with 16 cases.] *Rev Med Chil* 1983; **111:** 39-42. Spanish.
352. Kilgore TL, Jenkins DW. Laryngeal tuberculosis. *Chest* 1983; **83:** 139-141.
353. Manni H. Laryngeal tuberculosis in Tanzania. *J Laryngol Otol* 1983; **97:** 565-570.
354. Tsuda M, Matsunaga T, Ito H, Kataoka R, Sugimoto K, Tanaka A. [Recent tendency of laryngeal tuberculosis--a report pf 12 cases.] *Nihon Jibiinkoka Gakkai Kaiho* 1983; **86:** 1370-1376. Japanese
355. Vyravanathan S. Hoarseness in tuberculosis. *J Laryngol Otol* 1983; **97:** 523-525.
356. Ibekwe AO. Laryngeal tuberculosis in Nigeria. *East Afr Med J* 1984; **61:** 382-384.
357. Levenson MJ, Ingerman M, Grimes C, Robbett WF. Laryngeal tuberculosis: review of twenty cases. *Laryngoscope* 1984; **94:** 1094-1097.
358. Lo Bello SR, Lo Bello LE, Lo Bello MD, Scoto S. [Primary oral tuberculosis after dental extractions. Clinical, histopathological and epidemiological aspects.] *Minerva Stomatol* 1984; **33:** 41-48. Italian.
359. Shah AC, Abelson TI, Katz RL. Laryngeal tuberculosis. *Ear Nose Throat J* 1984; **63:** 175-179.
360. Worsaae N, Reibel J, Rechnitzer C. Tuberculous osteomyelitis of the mandible. *Br J Oral Maxillofac Surg* 1984; **22:** 93-98.
361. Beg MH, Marfani S. The larynx in pulmonary tuberculosis. *J Laryngol Otol* 1985; **99:** 201-203.
362. Bernat y Gili A, Garcia Garcia B, Herrero Herrero J. [Naso-pharyngeal lupus tuberculosis.] *An Otorrinolaringol Ibero Am* 1985; **12:** 495-502. Spanish.
363. Gombos F, Guida L, D'Ambrosio C, Zino G. [Tuberculosis of the oral cavity.] *Arch Stomatol (Napoli)* 1985; **26:** 19-30. Italian.
364. Lesser TH, Jefferis AF, Shaw HJ. Pseudotumoral mycobacterial infection in the head and neck: a clinical study. *J Laryngol Otol* 1985; **99:** 277-283.
365. Mani NJ. Tuberculosis initially diagnosed by asymptomatic oral lesions. Report of three cases. *J Oral Med* 1985; **40:** 39-42.
366. Dimitrowa J, Obreschkova E, Kirjakowa N. [Acute generalized miliary tuberculosis of the skin.] *Z Hautkr* 1986; **61:** 1549-1553. German.
367. James J, Ferguson MM. Orofacial granulomatosis presenting clinically as tuberculosis of cervical lymph nodes. *Br Dent J* 1986; **161:** 17-19.
368. Jan A. Primary laryngeal tuberculosis (a case report). *J Laryngol Otol* 1986; **100:** 605-606.
369. Malik MK, Kumar A, Bhatia BP. Changing pattern in laryngeal tuberculosis. *Indian J Chest Dis Allied Sci* 1986; **28:** 60-62.
370. Nishimura Y, Sakai K, Kitamura T, Hinata H, Yamashita H. [Radiation-induced cancers following radiotherapy of benign diseases: the second mail survey in Japan.] *Gan To Kagaku Ryoho* 1986; **13:** 1492-1498. Japanese.
371. Sammartino G, Battagliese G, Amato M, Laino A. [Primary tuberculosis complex of the oral cavity. 1. Its epidemiology, etiopathogenesis and the role of atypical mycobacteria.] *Minerva Stomatol* 1986; **35:** 1021-1026. Italian.
372. Sammartino G, Battagliese G, Mignogna MD, Amato M. [Primary tuberculosis complex of the oral cavity. II. The diagnostic problem.] *Minerva Stomatol* 1986; **35:** 1143-1146. Italian.
373. Siar CH, Ng KH, Kuppusamy I, Cheong ML, Sasidhar N. Secondary oral tuberculous ulcerations. *Dent J Malays* 1986; **9:** 29-32.
374. Strasding G, Draf W, Schoop HD. [Tuberculosis of the head, neck and ear--a rare disease?] *Hno* 1986; **34:** 66-70. German.
375. Szmeja Z, Kulczynski B, Wojtowicz J, Soboczynski R, Bolach Z. [Current features of laryngeal tuberculosis.] *Otolaryngol Pol* 1986; **40:** 267-271. Polish.
376. Carnegie Squires L, Paz Cordoves A, Nordet Cardona D, Tudela Coloma H. [Laryngeal tuberculosis. Report of 2 cases.] *Rev Cubana Med Trop* 1987; **39:** 133-139. Spanish.
377. du Plessis A, Hussey G. Laryngeal tuberculosis in childhood. *Pediatr Infect Dis J* 1987; **6:** 678-681.
378. Fearon B. Laryngeal problems in children. *Ann Otol Rhinol Laryngol* 1987; **96:** 124-126.
379. Fernandez Vozmediano JM, Romero Cabrera MA, Lasanta Villar J. [Tuberculous ulcer of the palate. Apropos of a case.] *Med Cutan Ibero Lat Am* 1987; **15:** 393-395. Spanish.
380. Haddad NM, Zaytoun GM, Hadi U. Tuberculosis of the soft palate: an unusual presentation of oral tuberculosis. *Otolaryngol Head Neck Surg* 1987; **97:** 91-92.
381. Hansen MA, Overgaard K, Grontved A. [Laryngeal tuberculosis]. *Ugeskr Laeger* 1987; **149:** 3336. Danish.
382. Kierzek A. [The achievements of Teodor Heryng in the treatment of laryngeal tuberculosis.] *Przegl Lek* 1987; **44:** 361-365. Polish.
383. Muthusamy E. Oral tuberculosis: two case reports. *Singapore Med J* 1987; **28:** 468-472.
384. Smallman LA, Clark DR, Raine CH, Proops DW, Shenoi PM. The presentation of laryngeal tuberculosis. *Clin Otolaryngol Allied Sci* 1987; **12:** 221-225.
385. Swart JG, de Flamingh DQ, Hamersma T. Histologically detected extrapulmonary tuberculosis in the head and neck region. A review of 222 cases. *S Afr Med J* 1987; **71:** 700-702.
386. Thaller SR, Gross JR, Pilch BZ, Goodman ML. Laryngeal tuberculosis as manifested in the decades 1963-1983. *Laryngoscope* 1987; **97:** 848-850.
387. Yaniv E. Tuberculous otitis: an underdiagnosed disease. *Am J Otolaryngol* 1987; **8:** 356-360.
388. Arnold M, Chan CY, Cheung SW, Van Hasselt CA, French GL. Diagnosis of nasopharyngeal tuberculosis by detection of tuberculostearic acid in formalin fixed, paraffin wax embedded tissue biopsy specimens. *J Clin Pathol* 1988; **41:** 1334-1336.
389. Ijaduola TG, Ademiluyi SA. Parotid fistula in children in the tropics. *Ann Trop Paediatr* 1988; **8:** 234-237.
390. Jan A. Tuberculosis of head and neck. Two case reports. *J Pak Med Assoc* 1988; **38:** 25-26.
391. Santoso FX, Witono ES, Makmuri MS, Santosa G. Laryngeal tuberculosis in a child (a case report). *Paediatr Indones* 1988; **28:** 60-66.
392. Abdel-Hadi M, Roquette M, Bartual J. [Tuberculosis in current clinical otorhinolaryngology.] *Acta Otorrinolaringol Esp* 1989; **40:** 189-194. Spanish.
393. Bryan PA, Lancken JH. Tuberculous mastoiditis and laryngitis: a case report. *Med J Aust* 1989; **150:** 41-43.
394. Chiang CY, Liu JJ, Chau WY, Hwang TZ. [Laryngeal tuberculosis simulating epiglottitis--a case report.] *Gaoxiong Yi Xue Ke Xue Za Zhi* 1989; **5:** 122-125. Chinese.
395. Chumakov FI, Luk'ianova MA. [Pathomorphosis of laryngeal tuberculosis.] *Vestn Otorinolaringol* 1989**:** 88-89. Russian.
396. Dvorski I. [Rare localizations of tuberculosis in the head and neck region.] *Vojnosanit Pregl* 1989; **46:** 366-369. Serbian.
397. el-Hakim IE, Langdon JD. Unusual presentation of tuberculosis of the head and neck region. Report of three cases. *Int J Oral Maxillofac Surg* 1989; **18:** 194-196.
398. Eleftheriadis I, Hatzifotiadis D. [Radiologic evaluation of cervical metastatic adenopathy.] *Hell Period Stomat Gnathopathoprosopike Cheir* 1989; **4:** 119-127. Greek.
399. Galietti F, Giorgis GE, Gandolfi G *et al.* Examination of 41 cases of laryngeal tuberculosis observed between 1975-1985. *Eur Respir J* 1989; **2:** 731-732.
400. Galietti F, Giorgis GE, Oliaro A *et al.* Tuberculosis of the larynx. Today. *Panminerva Med* 1989; **31:** 134-136.
401. Kapidzic A, Serbedzija D, Pejic M. [A specific retropharyngeal abscess in an older patient.] *Med Arh* 1989; **43:** 183-185. Croatian.
402. Ladron de Guevara R. [Tuberculous ulcer of the tongue: clinical case.] *Odontol Chil* 1989; **37:** 277-279. Spanish.
403. Palenque E, Contreras JD, Esteban J. [Laryngeal tuberculosis and isolation of M. bovis.] *Enferm Infecc Microbiol Clin* 1989; **7:** 455-456. Spanish.
404. Pedrol E, Estruch R, Barcelo J, Urbano-Marquez A. Tonsillar and pharyngeal tuberculosis in a patient without HIV antibodies. *J Infect Dis* 1989; **159:** 598.
405. Rupa V, Bhanu TS. Laryngeal tuberculosis in the eighties--an Indian experience. *J Laryngol Otol* 1989; **103:** 864-868.
406. Rupa V, Mathew J, Bhanu TS, Date A. Paediatric laryngeal tuberculosis presenting with stridor. *J Laryngol Otol* 1989; **103:** 787-788.
407. Soda A, Rubio H, Salazar M, Ganem J, Berlanga D, Sanchez A. Tuberculosis of the larynx: clinical aspects in 19 patients. *Laryngoscope* 1989; **99:** 1147-1150.
408. Stock CR, Goldman JL. Subglottic tuberculosis: a case report. *J Ky Med Assoc* 1989; **87:** 21-22.
409. Vandevelde L, Prive D, Vaes P, D'Olne D. [Tuberculous laryngitis: not so rare?.] *Acta Otorhinolaryngol Belg* 1989; **43:** 363-371. French.
410. Bundgaard N, Hansen IM. Tuberculosis in the Parotid Gland. *Ugeskrift for Laeger* 1990; **152:** 747-748.
411. Favia G, Chiaravalle G, Lacaita MG, Laforgia A, Fina A. [Orofacial tuberculosis. A general and anatomico-clinical analysis of 35 cases.] *Minerva Stomatol* 1990; **39:** 261-268. Italian.
412. Goh KL, Chang CM. Pharyngeal tuberculosis. *Trop Geogr Med* 1990; **42:** 75-77.
413. Kierzek A. [Trials of laryngeal tuberculosis treatment with cantharidin in the past century.] *Przegl Lek* 1990; **47:** 308-310. Polish.
414. Lau SK, Wei WI, Hsu C, Engzell UC. Efficacy of fine needle aspiration cytology in the diagnosis of tuberculous cervical lymphadenopathy. *J Laryngol Otol* 1990; **104:** 24-27.
415. Lee ST. Primary laryngeal tuberculosis mimicking carcinoma. *Southeast Asian J Trop Med Public Health* 1990; **21:** 630-631.
416. Moreno Sanchez D, Arevalo Serrano J, Dominguez Franjo MP, Castellano Tortajada G, Colina Ruiz-Delgado F, Belda Serna A. [Esophageal tuberculosis. Presentation of a case and review of the literature.] *Rev Esp Enferm Dig* 1990; **78:** 225-228. Spanish.
417. Anim JT, Dawlatly EE. Tuberculosis of the tonsil revisited. *West Afr J Med* 1991; **10:** 194-197.
418. Contreras Sanchez JD, Valdes Pons R, Palenque Mataix E, Almodovar Alvarez CL, Cervan Rubiales F, Alvarez Vicent JJ. [Primary laryngeal tuberculosis caused by Mycobacterium bovis.] *Acta Otorrinolaringol Esp* 1991; **42:** 75-77. Spanish.
419. Dimitrakopoulos I, Zouloumis L, Lazaridis N, Karakasis D, Trigonidis G, Sichletidis L. Primary tuberculosis of the oral cavity. *Oral Surg Oral Med Oral Pathol* 1991; **72:** 712-715.
420. Gay Escoda C, Buenechea Imaz R. [Oral tuberculosis: presentation of 3 cases.] *Rev Actual Odontoestomatol Esp* 1991; **51:** 41-44, 47-48. Spanish.
421. Held T, Rossler W, Reichelt A, Mielke M, Alexander M. [Tuberculosis of the larynx, oral cavity and pharynx.] *Dtsch Med Wochenschr* 1991; **116:** 1186-1190. German.
422. Kempf HG. [Laryngeal tuberculosis today. A case report.] *Hno* 1991; **39:** 70-72. German.
423. Kempf HG. [Tuberculosis of the larynx, oral cavity and pharynx.] *Dtsch Med Wochenschr* 1991; **116:** 1533. Geman.
424. Sasaki Y, Yamagishi F, Suzuki K *et al.* [Twelve cases of laryngeal tuberculosis.] *Kekkaku* 1991; **66:** 733-738. Japanese.
425. Ataman M, Sozeri B, Ozcelik T, Gedikoglu G. Tuberculosis of the parotid salivary gland. *Auris Nasus Larynx* 1992; **19:** 271-273.
426. Getson WR, Park YW. Pathologic quiz case 2. Laryngeal tuberculosis. *Arch Otolaryngol Head Neck Surg* 1992; **118:** 878-879, 881.
427. Riley EC, Amundson DE. Laryngeal tuberculosis revisited. *Am Fam Physician* 1992; **46:** 759-762.
428. Rowejones JM, Vowles R, Leighton SEJ, Freedman AR. Diffuse Tuberculous Parotitis. *Journal of Laryngology and Otology* 1992; **106:** 1094-1095.
429. Saleh EM, Mancuso AA, Stringer SP. CT of submucosal and occult laryngeal masses. *J Comput Assist Tomogr* 1992; **16:** 87-93.
430. Bhandarkar PD, Kasbekar VG, Shah RP, Hakim PP. Primary Tuberculous Ulcer of the Tongue. *Tropical Doctor* 1993; **23:** 41-42.
431. Burns JL. Laryngeal tuberculosis. *J Otolaryngol* 1993; **22:** 398.
432. Ferguson KA, McCormack DG. Tuberculosis involving the oral cavity. *Can J Infect Dis* 1993; **4:** 12-14.
433. Flanagan PM, McIlwain JC. Tuberculosis of the larynx in a lepromatous patient. *J Laryngol Otol* 1993; **107:** 845-847.
434. Hu HC, Zhang ZM, Jiang PF. [Tuberculosis ulcer of the oral mucosa: Report of 3 cases.] *Shanghai Kou Qiang Yi Xue* 1993; **2:** 179. Chinese.
435. Kashiwagi H, Ibe T, Takahashi Y, Teramura S, Hamaguchi Y, Taguchi O. [A case of oral tuberculosis suspected malignancy.] *Kekkaku* 1993; **68:** 495-499. Japanese.
436. Manolidis S, Frenkiel S, Yoskovitch A, Black M. Mycobacterial infections of the head and neck. *Otolaryngol Head Neck Surg* 1993; **109:** 427-433.
437. Meuthen I, Hummerich W, Kunstmann G, Brusis T, Spruth A. [Pharyngeal tuberculosis as a differential diagnosis to carcinoma.] *Hno* 1993; **41:** 37-40. German.
438. Oconnell JE, George MK, Speculand B, Pahor AL. Mycobacterial Infection of the Parotid-Gland - an Unusual Cause of Parotid Swelling. *Journal of Laryngology and Otology* 1993; **107:** 561-564.
439. Pestana E, Telo L, Gomes MJ, Amaral-Marques R. [Extrapulmonary tuberculosis.] *Acta Med Port* 1993; **6:** 175-180. Portuguese.
440. Ramadan HH, Tarazi AE, Baroudy FM. Laryngeal tuberculosis: presentation of 16 cases and review of the literature. *J Otolaryngol* 1993; **22:** 39-41.
441. Schneider W, Wolf SR, Solbach W. [Tuberculosis in the otorhinolaryngologic area. A still current differential diagnosis.] *Hno* 1993; **41:** 591-594. German.
442. Wang XL, Zhu SR, Chen XM, Hu CZ. [The clinical observation of 247 tuberculosis cases in Oral Maxillofacial region.] *Shanghai Kou Qiang Yi Xue* 1993; **2:** 134-135. Chinese.
443. Zivkovic D, Velojic D, Dordevic D. [Tuberculosis of the tongue in chronic hematogenous lung tuberculosis.] *Pneumologie (Stuttgart, Germany)* 1993; **47:** 36-37. German.
444. Daghfous MH, Nagi S, Benhajel H *et al.* Sialographic and Ultrasonographic Approach of Primary Salivary-Gland Tuberculosis - Report of 3 Cases. *Journal De Radiologie* 1994; **75:** 229-232.
445. de Pablo MA, Lamelas JA. [Lingual tuberculosis in an HIV-positive patient.] *Enfermedades infecciosas y microbiologia clinica* 1994; **12:** 361-362. Spanish.
446. Gallas D, Coste A, Bedbeder P, Peynegre R. [Current aspects of laryngeal tuberculosis. Apropos of 4 cases and review of the literature.] *Ann Otolaryngol Chir Cervicofac* 1994; **111:** 201-207. French.
447. Issing PR, Kempf HG, Ruck P, Lenarz T. [Primary clinical manifestation of tuberculosis as an incidental finding in the head and neck area.] *Laryngorhinootologie* 1994; **73:** 222-226. German.
448. Lozano I, Carbonell R, Perez F, Gimenez F. [Tuberculosis of the pharynx.] *An Otorrinolaringol Ibero Am* 1994; **21:** 535-542. Spanish.
449. Prada JL, Kindelan JM, Villanueva JL, Jurado R, Sanchezguijo P, Torrecisneros J. Tuberculosis of the Tongue in 2 Immunocompetent Patients. *Clinical Infectious Diseases* 1994; **19:** 200-202.
450. Ramadan HH, Wax MK. Laryngeal tuberculosis. *Otolaryngol Head Neck Surg* 1994; **111:** 155.
451. Remiszewski P, Wasowska H, Burakowska B. [Tuberculosis of the tongue--case report.] *Pneumonologia i alergologia polska* 1994; **62:** 295-298. Polish.
452. Soto Varela A, Lozano Ramirez A, del Rio Valeiras M, Labella Caballero T. [Laryngeal tuberculosis: report of 10 cases.] *Acta Otorrinolaringol Esp* 1994; **45:** 357-359. Spanish.
453. Tong MC, Van Hasselt CA. Laryngeal tuberculosis. *Otolaryngol Head Neck Surg* 1994; **111:** 687-688.
454. Vairaktaris E, Patsouris E, Papagiannopoulos N, Ragos B, Davaris P. Mycobacterial cervical lymphadenitis. A clinicopathological study of 3 cases. *J Craniomaxillofac Surg* 1994; **22:** 177-181.
455. Antico A. Oral tuberculosis: primary localisation in an elderly non-immunodepressed patient. *Tuber Lung Dis* 1995; **76:** 176-177.
456. Auregan G, Razafindrazaka N, Rakotomanana F, Rabarijaona L, Rakotoniaina N. [Laryngeal tuberculosis in Antananarivo.] *Arch Inst Pasteur Madagascar* 1995; **62:** 90-94. French.
457. Braden CR. Infectiousness of a university student with laryngeal and cavitary tuberculosis. Investigative team. *Clin Infect Dis* 1995; **21:** 565-570.
458. Bruzgielewicz A, Wysocki J, Osuch-Wojcikiewicz E. [Head and neck tuberculosis: a still urgent problem.] *Otolaryngol Pol* 1995; **49:** 566-573. Polish.
459. Cleary KR, Batsakis JG. Mycobacterial disease of the head and neck: current perspective. *Ann Otol Rhinol Laryngol* 1995; **104:** 830-833.
460. Demeter S, Fanning A, MacDonald F, Singh A. Contact tracing and follow-up of a case of laryngeal tuberculosis--Alberta. *Can Commun Dis Rep* 1995; **21:** 12-13.
461. Frieden TR, Simone PM, Castro KG. Case 34-1994: laryngeal tuberculosis. *N Engl J Med* 1995; **332:** 610; author reply 611.
462. Harlow RF, Rutkauskas JS. Tuberculosis risk in the hospital dental practice. *Spec Care Dentist* 1995; **15:** 50-55.
463. Jimenez-Saenz JM, Arazo P, Sanjuan F, Aguirre J. [Pharyngeal tuberculosis: unusual extrapulmonary involvement.] *Enferm Infecc Microbiol Clin* 1995; **13:** 378. Spanish.
464. Kikuchi K, Isii Y, Sugama Y, Kitamura S. [A case of laryngeal and tracheobronchial tuberculosis.] *Kekkaku* 1995; **70:** 591-594. Japanese.
465. Lee KC, Schecter G. Tuberculous infections of the head and neck. *Ear Nose Throat J* 1995; **74:** 395-399.
466. Lemaitre MP, Portet L, Londero A, Lettre MJ, Vincent D, Pradalier A. [Dysphonia disclosing laryngeal tuberculosis associated with latent pulmonary tuberculosis.] *Rev Med Interne* 1995; **16:** 371-372. French.
467. Matsumoto K, Ueda S, Horie T. [Pulmonary tuberculosis complicated with tuberculosis of oral mucosa, mandible and cervical lymph nodes.] *Kekkaku* 1995; **70:** 301-305. Japanese.
468. Prieto de Paula JM, Villamandos Nicas V, Rodriguez Rodriguez E, Borrego Pintado H. [Laryngeal tuberculosis. A report of 3 cases.] *Rev Clin Esp* 1995; **195:** 279-280. Spanish.
469. Ramadan HH, Wax MK. Laryngeal tuberculosis. A cause of stridor in children. *Arch Otolaryngol Head Neck Surg* 1995; **121:** 109-112.
470. Ristinen E, Winters T, Hattis PA. Case 34-1994: laryngeal tuberculosis. *N Engl J Med* 1995; **332:** 610-611; author reply 611.
471. Schafer DF. Case 34-1994: laryngeal tuberculosis. *N Engl J Med* 1995; **332:** 611.
472. Turchi R, Negri M, Vignali P. [Laryngeal tuberculosis: a case report]. *Acta Biomed Ateneo Parmense* 1995; **66:** 255-260. Italian.
473. Williams RG, Douglas-Jones T. Mycobacterium marches back. *J Laryngol Otol* 1995; **109:** 5-13.
474. Bhargava S, Watmough DJ, Chisti FA, Sathar SA. Tuberculosis of the parotid gland - Diagnosis by CT. *British Journal of Radiology* 1996; **69:** 1181-1183.
475. Buonomo S, Legrand W, Magremanne M. [Oral tuberculosis: apropos of a case.] *Acta Stomatol Belg* 1996; **93:** 33-36. French.
476. CeballosSalobrena A, AguirreUrizar JM, BaganSebastian JV. Oral manifestations associated with human immunodeficiency virus infection in a Spanish population. *Journal of Oral Pathology & Medicine* 1996; **25:** 523-526.
477. Eng HL, Lu SY, Yang CH, Chen WJ. Oral tuberculosis. *Oral Surg Oral Med Oral Pathol Oral Radiol Endod* 1996; **81:** 415-420.
478. Fortun J, Sierra C, Raboso E *et al.* [Tuberculosis of the otorhinolaryngologic region: laryngeal and extra-laryngeal forms.] *Enferm Infecc Microbiol Clin* 1996; **14:** 352-356. Spanish.
479. Fujimoto T, Morishima T, Ueda M. Probable BCG osteomyelitis of the hard palate: A case report. *International Journal of Oral and Maxillofacial Surgery* 1996; **25:** 145-146.
480. Garlicki A, Kowalski P, Kluba-Wojewoda U, Kleinrok K, Caban J. [Tuberculosis of the lymph nodes with esophageal-cutaneous fistula in a patient with AIDS.] *Pol Tyg Lek* 1996; **51:** 344-346. Polish.
481. Gil Tutor E. [Tuberculosis of the nose.] *An Otorrinolaringol Ibero Am* 1996; **23:** 641-650. Spanish.
482. Iqbal K, Udaipurwala IH, Khan SA, Jan AA, Jalisi M. Laryngeal involvement in pulmonary tuberculosis. *J Pak Med Assoc* 1996; **46:** 274-276.
483. Jawad J, ElZuebi F. Primary lingual tuberculosis: A case report. *Journal of Laryngology and Otology* 1996; **110:** 177-178.
484. Junquera Gutierrez LM, Alonso Vaquero D, Albertos Castro JM, Palacios Gutierrez JJ, Vicente Rodriguez JC. [Primary tuberculosis of the oral cavity.] *Rev Stomatol Chir Maxillofac* 1996; **97:** 3-6. French.
485. Kolokotronis A, Antoniadis D, Trigonidis G, Papanagiotou. Oral tuberculosis. *Oral Dis* 1996; **2:** 242-243.
486. Moon WK, Han MH, Chang KH *et al.* Laryngeal tuberculosis: CT findings. *AJR Am J Roentgenol* 1996; **166:** 445-449.
487. Moore M, Fleming KS, Sands L. A passenger with pulmonary/laryngeal tuberculosis: no evidence of transmission on two short flights. *Aviat Space Environ Med* 1996; **67:** 1097-1100.
488. Nachbar F, Classen V, Nachbar T, Meurer M, Schirren CG, Degitz K. Orificial tuberculosis: Detection by polymerase chain reaction. *British Journal of Dermatology* 1996; **135:** 106-109.
489. Panzarelli A, Acosta M, Garrido L. Tuberculosis cutis orificialis. *International Journal of Dermatology* 1996; **35:** 443-444.
490. Penfold CN, Revington PJ. A review of 23 patients with tuberculosis of the head and neck. *Br J Oral Maxillofac Surg* 1996; **34:** 508-510.
491. Phelan JA, Jimenez V, Tompkins DC. Tuberculosis. *Dent Clin North Am* 1996; **40:** 327-341.
492. Sharma K, Mehdiratta NK, Gupta AK. Tuberculosis of the parotid gland. *Canadian Journal of Surgery* 1996; **39:** 253-253.
493. Singh B, Balwally AN, Nash M, Har-El G, Lucente FE. Laryngeal tuberculosis in HIV-infected patients: a difficult diagnosis. *Laryngoscope* 1996; **106:** 1238-1240.
494. Toren A, Ackerstein A, Gazit D *et al.* Oral tuberculosis following autologous bone marrow transplantation for Hodgkin's disease with interleukin-2 and alpha-interferon immunotherapy. *Bone Marrow Transplant* 1996; **18:** 209-210.
495. Vidal R, Mayordomo C, Miravitlles M, Marti S, Torrella M, Lorente J. [Pulmonary and laryngeal tuberculosis. Study of 26 patients.] *Rev Clin Esp* 1996; **196:** 378-380. Spanish.
496. Weiner GM, Pahor AL. Tuberculous parotitis: Limiting the role of surgery. *Journal of Laryngology and Otology* 1996; **110:** 96-97.
497. Acero-Sanz J, Fernandez-Alba J, Concejo-Cutoli C, Berenguer J, Moreno-Celda V, Somacarrera ML. Gingival tuberculosis associated with oral candidosis in a HIV- positive patient. *Med Oral* 1997; **2:** 164-167.
498. Almeyda J, Tolley NS, Ghufoor K, Mochoulis G. Subglottic stenosis secondary to tuberculosis. *Int J Clin Pract* 1997; **51:** 402-403.
499. Aouadi A, Devars F, Duffas O, Traissac L. [Laryngeal tuberculosis: a diagnosis not to be forgotten.] *Rev Laryngol Otol Rhinol (Bord)* 1997; **118:** 181-182. French.
500. de Aguiar MC, Arrais MJ, Mato MJ, de Araujo VC. Tuberculosis of the oral cavity: a case report. *Quintessence Int* 1997; **28:** 745-747.
501. Delap TG, Lavy JA, Alusi G, Quiney RE. Tuberculosis presenting as a laryngeal tumour. *J Infect* 1997; **34:** 139-141.
502. Florio S, Ellis E, Frost DE. Persistent submandibular swelling after teeth extraction. *Journal of Oral and Maxillofacial Surgery* 1997; **55:** 390-397.
503. Franzen A, Franzen CK, Koegel K. Tuberculosis of the parotid gland: An unusual cause of a parotid neoplasm. *Laryngo-Rhino-Otologie* 1997; **76:** 308-311.
504. Gal G, Kaplan I, Calderon S, Carlson ER. Large perimandibular swelling. *Journal of Oral and Maxillofacial Surgery* 1997; **55:** 1134-1143.
505. Grobholz R, Bittinger A, Gerdes B, Rothmund M. [Development of a thyroid carcinoma after irradiation of the head/neck region.] *Dtsch Med Wochenschr* 1997; **122:** 362-365. German.
506. Hathiram BT, Grewal DS, Irani DK, Tankwal PM, Patankar M. Tuberculoma of the cheek: a case report. *Journal of Laryngology and Otology* 1997; **111:** 872-873.
507. Houghton DJ, Bennett JD, Rapado F, Small M. Laryngeal tuberculosis: an unsuspected danger. *Br J Clin Pract* 1997; **51:** 61-62.
508. Kandiloros DC, Nikolopoulos TP, Ferekidis EA *et al.* Laryngeal tuberculosis at the end of the 20th century. *J Laryngol Otol* 1997; **111:** 619-621.
509. Kim MD, Kim DI, Yune HY *et al.* CT findings of laryngeal tuberculosis: comparison to laryngeal carcinoma. *J Comput Assist Tomogr* 1997; **21:** 29-34.
510. Kobayashi T, Sato M, Onoi Y *et al.* [A case of pulmonary tuberculosis complicated with gingival lesions.] *Kekkaku* 1997; **72:** 411-414. Japanese.
511. Lightfoot SA. Laryngeal tuberculosis masquerading as carcinoma. *J Am Board Fam Pract* 1997; **10:** 374-376.
512. Moon WK, Han MH, Chang KH *et al.* CT and MR imaging of head and neck tuberculosis. *Radiographics* 1997; **17:** 391-402.
513. Pease BC, Hoasjoe DK, Stucker FJ. Videostroboscopic findings in laryngeal tuberculosis. *Otolaryngol Head Neck Surg* 1997; **117:** S230-234.
514. Ramesh V. Tuberculoma of the tongue presenting as macroglossia. *Cutis* 1997; **60:** 201-202.
515. Ruiz Franco MF, Gil Velez M, Hellin Meseguer D, Paya Perez L, Vera Quiles F. [A case of tuberculous epiglottitis: an unusual form of presentation.] *Acta Otorrinolaringol Esp* 1997; **48:** 501-503. Spanish.
516. Talib SH, Singh J. A study on interrelationship of 60 HIV positive cases with coexistent oral candidosis and tuberculosis. *Indian J Pathol Microbiol* 1997; **40:** 377-382.
517. Tu HY, Li HY, Huang TS. Laryngeal tuberculosis: a series of 46 patients. *Changgeng Yi Xue Za Zhi* 1997; **20:** 94-99.
518. Agarwal P, Bais AS. A clinical and videostroboscopic evaluation of laryngeal tuberculosis. *J Laryngol Otol* 1998; **112:** 45-48.
519. Bishara J, Calderon S, Okon E, Shevach I, Maimon S, Pitlik S. Coexisting extrapulmonary tuberculosis and malignancy. *American Journal of Medicine* 1998; **105:** 443-446.
520. de Bree R, Chung RP, van Aken J, van den Brekel MW. [Tuberculosis of the larynx.] *Ned Tijdschr Geneeskd* 1998; **142:** 1676-1680. Dutch.
521. Diaz Alcover C, Sanchez Alcon MD, Seijas Rosales T, Perez Garrigues T, Gisbert Jaudenes V, Tortosa Navarro V. [Primary tuberculosis of the lingual tonsil. A case report]. *Acta Otorrinolaringol Esp* 1998; **49:** 591-593.
522. Erasmus JH, Thompson IOC, van der Westhuijzen AJ. Tuberculous osteomyelitis of the mandible: Report of a case. *Journal of Oral and Maxillofacial Surgery* 1998; **56:** 1355-1358.
523. Gallardo A, Ramirez R, Martinez R, Castilla JM, Gallardo J, Banon R. [Pharyngeal tuberculosis: a clinical case.] *Acta Otorrinolaringol Esp* 1998; **49:** 587-590. Spanish.
524. Gupta A, Shinde KJ, Bhardwaj I. Primary lingual tuberculosis: a case report. *J Laryngol Otol* 1998; **112:** 86-87.
525. Kharoubi S. [Pharyngeal tuberculosis: an analytical study and report of 10 cases.] *Rev Laryngol Otol Rhinol (Bord)* 1998; **119:** 203-207. French.
526. Konishi K, Yamane H, Iguchi H *et al.* Study of tuberculosis in the field of otorhinolaryngology in the past 10 years. *Acta Otolaryngol Suppl* 1998; **538:** 244-249.
527. Kothari P, Bartella L, Carter J, Chan O, Piper K. Tuberculosis of the mandible in a child. *Journal of Laryngology and Otology* 1998; **112:** 585-587.
528. Kumoi K. [Head and neck tuberculosis.] *Nihon Rinsho* 1998; **56:** 3148-3152. Japanese.
529. Mehta J, Chaudhary N, Mittal A, Motwani G, Gandotra SC. Tuberculosis of tongue. *Indian J Otolaryngol Head Neck Surg* 1998; **50:** 284-286.
530. Miyahara H, Sato T, Yoshino K. Radiation-induced cancers of the head and neck region. *Acta Otolaryngol Suppl* 1998; **533:** 60-64.
531. Perez C, Torroba L, Gonzalez M, Vives R, Guarch R. Unusual presentation of tuberculous rheumatism (Poncet's disease) with oral ulcers and tuberculid. *Clin Infect Dis* 1998; **26:** 1003-1004.
532. Plaza Mayor G, Perez Martinez C, Sierra Granon C *et al.* [Laryngeal tuberculosis and laryngeal cancer.] *An Otorrinolaringol Ibero Am* 1998; **25:** 387-397. Spanish.
533. Sharma HS, Kurl DN, Kamal MZ. Tuberculoid granulomatous lesion of the pharynx--review of the literature. *Auris Nasus Larynx* 1998; **25:** 187-191.
534. Singh B, Balwally AN, Har-El G, Lucente FE. Isolated cervical tuberculosis in patients with HIV infection. *Otolaryngol Head Neck Surg* 1998; **118:** 766-770.
535. Suoglu Y, Erdamar B, Colhan I, Katircioglu OS, Cevikbas U. Tuberculosis of the parotid gland. *Journal of Laryngology and Otology* 1998; **112:** 588-591.
536. Tato AM, Pascual J, Orofino L *et al.* Laryngeal tuberculosis in renal allograft patients. *Am J Kidney Dis* 1998; **31:** 701-705.
537. Ulloa R, Avila ML, Soto M *et al.* Laryngeal tuberculosis. *Pediatr Infect Dis J* 1998; **17:** 758-760.
538. Ying M, Ahuja AT, Evans R, King W, Metreweli C. Cervical lymphadenopathy: sonographic differentiation between tuberculous nodes and nodal metastases from non-head and neck carcinomas. *J Clin Ultrasound* 1998; **26:** 383-389.
539. Chumakov FI, Luk'ianova MA. [On aspects of laryngeal tuberculosis.] *Vestn Otorinolaringol* 1999**:** 40-42. Russian.
540. Chumakov FI, Luk'ianova MA. [Pathomorphology of laryngeal tuberculosis. (from 50 years of own observations).] *Probl Tuberk* 1999**:** 39-40. Russian.
541. Drolet MJ, Boisvert R, Dery S, Laliberte D. [Epidemiological study of a tuberculosis case in a large manufacturing enterprise in Quebec.] *Can J Public Health* 1999; **90:** 156-159. French.
542. Eisenkraft BL, Som PM. The spectrum of benign and malignant etiologies of cervical node calcification. *AJR Am J Roentgenol* 1999; **172:** 1433-1437.
543. Gamble EA, Davison AG. Chronic lip ulceration in association with an abnormal chest radiograph. *Respiration* 1999; **66:** 477-478.
544. Hajioff D, Snow MH, Thaker H, Wilson JA. Primary tuberculosis of the posterior oropharyngeal wall. *J Laryngol Otol* 1999; **113:** 1029-1030.
545. Jha V, Kohli HS, Sud K *et al.* Laryngeal tuberculosis in renal transplant recipients. *Transplantation* 1999; **68:** 153-155.
546. Marcos Ordonez M, Benito Orejas JI, Blasco Gutierrez MJ, Morais Perez D, Ramirez Cano B. [Oropharyngeal tuberculosis. Report of a case in a lingual tonsil.] *Acta Otorrinolaringol Esp* 1999; **50:** 575-578. Spanish.
547. Panek B, Chyczewska E, Mroz RM. [Tuberculosis of the tongue.] *Pneumonologia i alergologia polska* 1999; **67:** 477-480. Polish.
548. Sah SP, Raj GA, Bahadur T. Chronic ulceration of the tongue and laryngitis: First clinical sign of asymptomatic pulmonary tuberculosis. *Journal of Infection* 1999; **39:** 163-164.
549. Aguirre Garcia F, Fuertes Martin A, Guillen Guerrero VS *et al.* [Tongue tuberculosis as the first expression of the lung process]. *An Otorrinolaringol Ibero Am* 2000; **27:** 111-118. Spanish.
550. Aktogu S, Eris FN, Dinc ZA, Tibet G. Tuberculosis of the tongue secondary to pulmonary tuberculosis. *Monaldi Arch Chest Dis* 2000; **55:** 287-288.
551. Anil S, Ellepola AN, Samaranayake LP, Beena VT. Tuberculous ulcer of the tongue as presenting feature of pulmonary tuberculosis and HIV infection. *Gen Dent* 2000; **48:** 458-461.
552. Chumakov FI, Gerasimenko NV. [Isolated tuberculosis of pharyngeal and palatine tonsils in child.] *Vestn Otorinolaringol* 2000**:** 58. Russian.
553. Galletti F, Freni F, Bucolo S *et al.* [Laryngeal tuberculosis: considerations on the most recent clinical and epidemiological data and presentation of a case report.] *Acta Otorhinolaryngol Ital* 2000; **20:** 196-201. Italian.
554. Harney M, Hone S, Timon C, Donnelly M. Laryngeal tuberculosis: an important diagnosis. *J Laryngol Otol* 2000; **114:** 878-880.
555. Hata T, Hosoda M. First symptom of multifocal skeletal tuberculosis in children. *British Journal of Oral & Maxillofacial Surgery* 2000; **38:** 572-573.
556. Jawahar MS. Scrofula revisited: an update on the diagnosis and management of tuberculosis of superficial lymph nodes. *Indian J Pediatr* 2000; **67:** S28-33.
557. Koksal D, Acican T, Kanat F, Durmaz G, Ataoglu O, Cobanli B. Tuberculous ulcer of the tongue secondary to pulmonary tuberculosis. *Australian and New Zealand Journal of Medicine* 2000; **30:** 518-519.
558. Lang S, Nerlich A, Issing WJ. [The interesting case No. 39. Differential diagnosis of acute antibiotic-resistant pharyngitis.] *Laryngorhinootologie* 2000; **79:** 616-618. German.
559. Mert A, Ozaras R, Bilir M *et al.* Primary tuberculosis of the parotid gland. *International journal of infectious diseases : IJID : official publication of the International Society for Infectious Diseases* 2000; **4:** 229-230.
560. Mignogna MD, Muzio LL, Favia G *et al.* Oral tuberculosis: a clinical evaluation of 42 cases. *Oral Dis* 2000; **6:** 25-30.
561. Sherrell JC, Powers P, Norwood JM. A case of tuberculosis in Memphis. *Am J Med Sci* 2000; **320:** 403-405.
562. Shin JE, Nam SY, Yoo SJ, Kim SY. Changing trends in clinical manifestations of laryngeal tuberculosis. *Laryngoscope* 2000; **110:** 1950-1953.
563. Sierra C, Fortun J, Barros C *et al.* Extra-laryngeal head and neck tuberculosis. *Clin Microbiol Infect* 2000; **6:** 644-648.
564. Sutbeyaz Y, Ucuncu H, Murat Karasen R, Gundogdu C. The association of secondary tonsillar and laryngeal tuberculosis: a case report and literature review. *Auris Nasus Larynx* 2000; **27:** 371-374.
565. Unzaga MJ, Zubero Z, Pardo C, Calvo F, Cisterna R. [Laryngeal tumor of laryngeal tuberculosis?] *Enferm Infecc Microbiol Clin* 2000; **18:** 46. Spanish.
566. Weiler Z, Nelly P, Baruchin AM, Oren S. Diagnosis and treatment of cervical tuberculous lymphadenitis. *J Oral Maxillofac Surg* 2000; **58:** 477-481.
567. Yencha MW, Linfesty R, Blackmon A. Laryngeal tuberculosis. *Am J Otolaryngol* 2000; **21:** 122-126.
568. Al-Serhani AM. Mycobacterial infection of the head and neck: presentation and diagnosis. *Laryngoscope* 2001; **111:** 2012-2016.
569. Al-Serhani AM, Al-Mazrou K. Pharyngeal tuberculosis. *Am J Otolaryngol* 2001; **22:** 236-240.
570. Bhatt AP, Jayakrishnan A. Tuberculous osteomyelitis of the mandible: a case report. *International journal of paediatric dentistry* 2001; **11:** 304-308.
571. Cakan A, Mutlu Z, Ozsoz A, Erbaycu AE, Unal T, Koyuncu BO. Tuberculosis of oral mucosa. *Monaldi Arch Chest Dis* 2001; **56:** 315-317.
572. Chumakov FI. [Laryngeal tuberculosis]. *Vestn Otorinolaringol* 2001**:** 63-65. Russian.
573. El Mustafa F, Abdou N, Benyounes R *et al.* Laryngeal tuberculosis with tongue involvement in a renal transplant recipient. *Nephrol Dial Transplant* 2001; **16:** 1958-1959.
574. Essaadi M, Raji A, Detsouli M *et al.* [Laryngeal tuberculosis: apropos of 15 cases]. *Rev Laryngol Otol Rhinol (Bord)* 2001; **122:** 125-128. French.
575. Gupta N, Nuwal P, Gupta ML, Gupta RC, Dixit RK. Primary tuberculosis of soft palate. *Indian J Chest Dis Allied Sci* 2001; **43:** 119-121.
576. Iype EM, Ramdas K, Pandey M *et al.* Primary tuberculosis of the tongue: report of three cases. *British Journal of Oral & Maxillofacial Surgery* 2001; **39:** 402-403.
577. Lacosta Nicolas JL, Calzada Uriondo G. [Laryngeal tuberculosis. Report of 4 cases.] *An Otorrinolaringol Ibero Am* 2001; **28:** 459-465. Spanish.
578. Molodtsov VG, Biriukova LA, Shipkov AV, Dobzhanskii AV. [A case of extrapulmonary tuberculosis with laryngeal and intestinal involvement.] *Vestn Otorinolaringol* 2001**:** 57. Russian.
579. Montejo M, Alonso M, Aguirrebengoa K *et al.* [Laryngeal tuberculosis: study of 11 cases.] *Acta Otorrinolaringol Esp* 2001; **52:** 53-56. Spanish.
580. Mukherjee S, Sengupta A, Chakraborty J. Laryngeal Tuberculosis in MDR-TB presenting as Laryngeal Carcinoma. *Indian J Otolaryngol Head Neck Surg* 2001; **53:** 321-322.
581. Muranjan SN, Kirtane MV. Tubercular laryngeal abscess. *J Laryngol Otol* 2001; **115:** 660-662.
582. Nielsen VE, Skott MN. [Laryngeal tuberculosis. A rare, but important differential diagnosis to chronic laryngitis.] *Ugeskr Laeger* 2001; **163:** 2365-2367. Danish.
583. Oestreicher Y, Feinmeser R. [Laryngeal tuberculosis is not such a rare disease.] *Harefuah* 2001; **140:** 998-1001, 1120. Hebrew.
584. Richter B, Fradis M, Kohler G, Ridder GJ. Epiglottic tuberculosis: differential diagnosis and treatment. Case report and review of the literature. *Ann Otol Rhinol Laryngol* 2001; **110:** 197-201.
585. Savenkova MS, Golubtsova EE, Legkova TP. [Lethal outcome in a child with laryngeal tuberculosis.] *Vestn Otorinolaringol* 2001**:** 52-53. Russian.
586. Von Arx DP, Husain A. Oral tuberculosis. *Br Dent J* 2001; **190:** 420-422.
587. Watanabe M, Nakayama T, Koduka Y *et al.* Mycobacterium tuberculosis infection within Warthin's tumor: Report of two cases. *Pathology International* 2001; **51:** 797-801.
588. Bailleux S, Poissonnet G, Poudenx M, Ettore F, Dassonville O, Demard F. [Laryngeal tuberculosis: report of a case]. *Rev Laryngol Otol Rhinol (Bord)* 2002; **123:** 171-173. French.
589. Carnelio S, Rodrigues G. Primary lingual tuberculosis: a case report with review of literature. *Journal of oral science* 2002; **44:** 55-57.
590. Caylan R, Aydin K, Caylan R. Oropharyngeal tuberculosis causing severe odynophagia and dysphagia. *Eur Arch Otorhinolaryngol* 2002; **259:** 229-230.
591. Chumakov FI, Khmeleva RI. [Head and neck lymph node lesions]. *Vestn Otorinolaringol* 2002**:** 27-29. Russian.
592. Feldmann H. [Diagnosis and therapy of diseases of the larynx in the history of medicine. Part III. After the invention of laryngoscopy.] *Laryngorhinootologie* 2002; **81:** 596-604. German.
593. Galli J, Nardi C, Contucci AM, Cadoni G, Lauriola L, Fantoni M. Atypical isolated epiglottic tuberculosis: a case report and a review of the literature. *Am J Otolaryngol* 2002; **23:** 237-240.
594. Ilyas SE, Chen FF, Hodgson TA, Speight PM, Lacey CJ, Porter SR. Labial tuberculosis: a unique cause of lip swelling complicating HIV infection. *HIV Med* 2002; **3:** 283-286.
595. Kasztelan A, Lots J, Polberg K, Smialek M. [Rare case of laryngeal tuberculosis.] *Otolaryngol Pol* 2002; **56:** 733-735. Polish.
596. Kossowski M, Conessa C, Clement P, Roguet E, Verdalle P, Poncet JL. [Current aspects of laryngeal tuberculosis: a report of four cases.] *Ann Otolaryngol Chir Cervicofac* 2002; **119:** 281-286. French.
597. Landa LE, Kathju S, Nepomuceno-Perez MC, Gordon C, Sotereanos GC. Tuberculous granuloma and adenoid cystic carcinoma presenting as a single buccal space mass. *Journal of Craniofacial Surgery* 2002; **13:** 533-537.
598. Lin CJ, Kang BH, Wang HW. Laryngeal tuberculosis masquerading as carcinoma. *Eur Arch Otorhinolaryngol* 2002; **259:** 521-523.
599. Madhuri, Mohan C, Sharma ML. Posterior oro-pharyngeal wall tuberculosis. *Indian J Otolaryngol Head Neck Surg* 2002; **54:** 152-153.
600. Nishiike S, Irifune M, Doi K, Sawada T, Kubo T. Laryngeal tuberculosis: a report of 15 cases. *Ann Otol Rhinol Laryngol* 2002; **111:** 916-918.
601. Porras Alonso E, Martin Mateos A, Perez-Requena J, Avalos Serrano E. Laryngeal tuberculosis. *Rev Laryngol Otol Rhinol (Bord)* 2002; **123:** 47-48.
602. Ragesh KP, Chana RS, Varshney PK, Naim M. Head and neck masses in children: A clinicopathological study. *Indian J Otolaryngol Head Neck Surg* 2002; **54:** 268-271.
603. To EW, Williams MD, Tsang WM, Pang PC, Lai E. Tuberculosis and wound healing in head and neck cancer. *Asian J Surg* 2002; **25:** 66-67.
604. Yamamoto K, Iwata F, Nakamura A *et al.* Tonsillar tuberculosis associated with pulmonary and laryngeal foci. *Intern Med* 2002; **41:** 664-666.
605. Anupama, Hemanth KS, Mondal SK, Rai G. Sinonasal tuberculosis in diabetics : An unusual presentation and diagnosis. *Indian J Otolaryngol Head Neck Surg* 2003; **55:** 121-123.
606. Brown RS, Farquharson AA, Nasseri S. Bilateral bone loss of the maxilla. *Oral Surgery Oral Medicine Oral Pathology Oral Radiology and Endodontics* 2003; **96:** 6-11.
607. Eguchi J, Ishihara K, Watanabe A, Fukumoto Y, Okuda K. PCR method is essential for detecting Mycobacterium tuberculosis in oral cavity samples. *Oral Microbiol Immunol* 2003; **18:** 156-159.
608. el Hag IA, Chiedozi LC, al Reyees FA, Kollur SM. Fine needle aspiration cytology of head and neck masses. Seven years' experience in a secondary care hospital. *Acta Cytol* 2003; **47:** 387-392.
609. Hofman W, Selva E, Musso S, Odin JC, Dellamonica P, Hofman P. Tuberculosis: a rare and misleading etiology of tongue's ulcer. *Annales De Pathologie* 2003; **23:** 261-265.
610. Kenmochi M, Ohashi T, Nishino H *et al.* A case report of difficult diagnosis in the patient with advanced laryngeal tuberculosis. *Auris Nasus Larynx* 2003; **30 Suppl:** S131-134.
611. Kiuchi N, Irifune M, Koizuka I. [Report of 2 cases of tuberculous retoropharygeal abscess in adults.] *Nihon Jibiinkoka Gakkai Kaiho* 2003; **106:** 510-513. Japanese.
612. Magina S, Lisboa C, Resende C *et al.* Tuberculosis in a child presenting as asymptomatic oropharyngeal and laryngeal lesions. *Pediatr Dermatol* 2003; **20:** 429-431.
613. Memon GA, Khushk IA. Primary tuberculosis of tongue. *Journal of the College of Physicians and Surgeons--Pakistan : JCPSP* 2003; **13:** 604-605.
614. Miura K, Kum Y, Han G, Tsutsui Y. Radiation-induced laryngeal angiosarcoma after cervical tuberculosis and squamous cell carcinoma: case report and review of the literature. *Pathol Int* 2003; **53:** 710-715.
615. Muecke C, Brassard P, Isler M, Tannenbaum TN, Menzies D, Carsley J. Contact investigation of a case of pulmonary and laryngeal tuberculosis. *Can Commun Dis Rep* 2003; **29:** 91-92.
616. Munck K, Mandpe AH. Mycobacterial infections of the head and neck. *Otolaryngol Clin North Am* 2003; **36:** 569-576.
617. Nagabhushana D, Balaji Rao B, Rajeshwari A, Mamatha GP. Oral tuberculosis : a case report. *J Indian Soc Pedod Prev Dent* 2003; **21:** 16-18.
618. Rivera H, Correa MF, Castillo-Castillo S, Nikitakis NG. Primary oral tuberculosis: a report of a case diagnosed by polymerase chain reaction. *Oral Dis* 2003; **9:** 46-48.
619. Rizzo PB, Da Mosto MC, Clari M, Scotton PG, Vaglia A, Marchiori C. Laryngeal tuberculosis: an often forgotten diagnosis. *Int J Infect Dis* 2003; **7:** 129-131.
620. Singh K, Kaur G, Parmar TL. Pseudo tumoral laryngeal tuberculosis. *Indian Pediatr* 2003; **40:** 49-52.
621. Soman D, Davies SJ. A suspected case of tuberculosis of the temporomandibular joint. *Br Dent J* 2003; **194:** 23-24.
622. Srirompotong S, Yimtae K, Srirompotong S. Tuberculosis in the upper aerodigestive tract and human immunodeficiency virus coinfections. *J Otolaryngol* 2003; **32:** 230-233.
623. Tsikoudas A. Management pathways and the surgical diagnosis of tuberculous lymphadenitis: can they be improved? The Bradford experience. *ORL J Otorhinolaryngol Relat Spec* 2003; **65:** 261-265.
624. Bartnik W, Bartnik-Krystalska A. [Tuberculosis of the larynx and pharynx in hospitalized patients in ENT Department of Voivodeship Hospital in Kalisz.] *Otolaryngol Pol* 2004; **58:** 517-520. Polish.
625. Bayazit YA, Bayazit N, Namiduru M. Mycobacterial cervical lymphadenitis. *ORL J Otorhinolaryngol Relat Spec* 2004; **66:** 275-280.
626. Chaudhary S, Kalra N, Gomber S. Tuberculous osteomyelitis of the mandible: A case report in a 4-year-old child. *Oral Surgery Oral Medicine Oral Pathology Oral Radiology and Endodontics* 2004; **97:** 603-606.
627. Chou YH, Tiu CM, Liu CY *et al.* Tuberculosis of the parotid gland - Sonographic manifestations and sonographically guided aspiration. *Journal of Ultrasound in Medicine* 2004; **23:** 1275-1281.
628. Imamura M, Kakihara T, Yamamoto K, Imat C, Tanaka A, Uchiyama M. Primary tuberculous osteomyelitis of the mandible. *Pediatrics International* 2004; **46:** 736-739.
629. Kavala M, Sudogan S, Can B, Sarigul S. Granulomatous cheilitis resulting from a tuberculide. *International Journal of Dermatology* 2004; **43:** 524-527.
630. Kontopoulou T, Fanourgiakis P, Samarkos M *et al.* Tuberculosis of the parotid gland: case report and literature review. *Medecine Et Maladies Infectieuses* 2004; **34:** 488-490.
631. Krecicki T, Zalesska-Krecicka M, Zatonski T, Jankowska R, Skrzydlewska-Kaczmarek B. Laryngeal tuberculosis. *Lancet Infect Dis* 2004; **4:** 57.
632. Kundu S, Das S, Dey A, Sengupta A. Tuberculosis of parotid gland - a rare clinical entity. *Indian journal of otolaryngology and head and neck surgery : official publication of the Association of Otolaryngologists of India* 2004; **56:** 57-58.
633. Michalak A, Wojtas G, Kidawa I, Tylzanowska-Nitek K. [Tuberculosis of the tongue in a patient with disseminated pulmonary tuberculosis]. *Pneumonologia i alergologia polska* 2004; **72:** 28-31.
634. Nawaz G, Khan MR. Primary sinonasal tuberculosis in north-west Pakistan. *J Coll Physicians Surg Pak* 2004; **14:** 221-224.
635. Nayar RC, Al Kaabi J, Ghorpade K. Primary nasal tuberculosis: a case report. *Ear Nose Throat J* 2004; **83:** 188-191.
636. Nwaorgu OG, Onakoya PA, Ibekwe TS, Bakari A. Hoarseness in adult Nigerians: a University College Hospital Ibadan experience. *Niger J Med* 2004; **13:** 152-155.
637. Qidwai W, Rehman S. A young man with hoarseness of voice. *J Ayub Med Coll Abbottabad* 2004; **16:** 73-74.
638. Sezer B, Zeytinoglu M, Tuncay U, Unal T. Oral mucosal ulceration: a manifestation of previously undiagnosed pulmonary tuberculosis. *J Am Dent Assoc* 2004; **135:** 336-340.
639. Tornero Salto J, Nogues Orpi JJ, Gonzalez Compta X, Cisa Lluis E, Domenech Juan I, Dicenta Sousa M. [Current aspects of laryngeal tuberculosis.] *An Otorrinolaringol Ibero Am* 2004; **31:** 159-165. Spanish.
640. Yigit O, Cinar U, Uslu Coskun B, Basak T. Tuberculous ulcer of the tongue: a case report. *Kulak Burun Bogaz Ihtis Derg* 2004; **13:** 98-101.
641. Baskota DK, Prasad R, Sinha BK, Amatya RC. Frequency and effective treatment of ulcers and sinuses in cases of tuberculous cervical lymphadenitis. *J Coll Physicians Surg Pak* 2005; **15:** 157-159.
642. Chaturvedi P, Pai PS, Pathak KA, D'Cruz A K. Radiology quiz case 3: laryngeal tuberculosis. *Arch Otolaryngol Head Neck Surg* 2005; **131:** 740, 743-744.
643. Choudhury N, Bruch G, Kothari P, Rao G, Simo R. 4 years' experience of head and neck tuberculosis in a south London hospital. *J R Soc Med* 2005; **98:** 267-269.
644. Coscaron Blanco E, Santa Cruz Ruiz S, Serradilla Lopez JM. [Tuberculous epiglotittis, an atypical form of laryngeal tuberculosis. Presentation of a case and revision of litterature.] *An Otorrinolaringol Ibero Am* 2005; **32:** 55-63. Spanish.
645. Domanska-Strycharska M, Czak W. [Tuberculosis of larynx--still current problem.] *Otolaryngol Pol* 2005; **59:** 689-692. Polish.
646. Feller L, Anagnostopoulos C, Bouckaert M, Raubenheimer EJ. HIV/TB co-infection: literature review and report of multiple tuberculosis oral ulcers. *Sadj* 2005; **60:** 330-332, 343.
647. Flaitz CM, Nichols CM. Oral and maxillofacial pathology case of the month. Tuberculous lymphadenitis. *Tex Dent J* 2005; **122:** 280-281, 286-287.
648. Girszyn N, Belmekki A, Duterque M *et al.* Lingual tuberculosis associated with disseminated tuberculosis. *Annales De Dermatologie Et De Venereologie* 2005; **132:** 368-369.
649. Heigis G, Krimmel M, Hoffmann J, Kaiserling E, Reinert S. [Oral manifestation of miliary tuberculosis.] *Mund-, Kiefer- und Gesichtschirurgie : MKG* 2005; **9:** 180-183. German.
650. Iseri M, Aydiner O, Celik L, Peker O. Tuberculosis of the parotid gland. *Journal of Laryngology and Otology* 2005; **119:** 311-313.
651. Ito FA, de Andrade CR, Vargas PA, Jorge J, Lopes MA. Primary tuberculosis of the oral cavity. *Oral Dis* 2005; **11:** 50-53.
652. Jurkiewicz-Lobodzinska M, Pajuro R. [Multiorgan tuberculosis with unusual clinical picture and a fulminant course.] *Otolaryngologia polska = The Polish otolaryngology* 2005; **59:** 351-355. Polish.
653. Kim YH, Jeong WJ, Jung KY, Sung MW, Kim KH, Kim CS. Diagnosis of major salivary gland tuberculosis: Experience of eight cases and review of the literature. *Acta Oto-Laryngologica* 2005; **125:** 1318-1322.
654. Malhotra P, Arora VK, Singh N, Bhatia A. Algorithm for cytological diagnosis of nonneoplastic lesions of the salivary glands. *Diagnostic Cytopathology* 2005; **33:** 90-94.
655. Miziara ID. Tuberculosis affecting the oral cavity in Brazilian HIV-infected patients. *Oral Surg Oral Med Oral Pathol Oral Radiol Endod* 2005; **100:** 179-182.
656. Ozudogru E, Cakli H, Altuntas EE, Gurbuz MK. Effects of laryngeal tuberculosis on vocal fold functions: case report. *Acta Otorhinolaryngol Ital* 2005; **25:** 374-377.
657. Perrotti V, Petrone G, Rubini C, Fioroni M, Piattelli A. Tuberculosis of buccal mucosa. *Journal of Otolaryngology* 2005; **34:** 274-276.
658. Pino Rivero V, Marcos Garcia M, Gonzalez Palomino A *et al.* [Laryngeal tuberculosis masquerading as carcinoma. Report of one case and literature review]. *An Otorrinolaringol Ibero Am* 2005; **32:** 47-53. Spanish.
659. Plaza Mayor G, Pinedo Moraleda F, Ferrando Alvarez-Cortina J, Espinosa Gimeno A, de los Santos Granados G. [Otalgia as presentation of primary nasopharynx tuberculosis.] *An Otorrinolaringol Ibero Am* 2005; **32:** 585-591. Spanish.
660. Polok A, Namyslowski G, Scierski W, Czecior E, Mrowka-Kata K, Gac B. [Tuberculosis within the laryngologic organs.] *Pol Merkur Lekarski* 2005; **19:** 473-474. Polish.
661. Ramirez-Amador V, Anaya-Saavedra G, Gonzalez-Ramirez I *et al.* Lingual ulcer as the only sign of recurrent mycobacterial infection in an HIV/AIDS-infected patient. *Medicina oral, patologia oral y cirugia bucal* 2005; **10:** 109-114.
662. Scott P, Middlefell LS, Fabbroni G, Mitchell DA. Interesting case: oral presentation of tuberculosis. *Br J Oral Maxillofac Surg* 2005; **43:** 492.
663. Sheahan P, Hafidh M, Toner M, Timon C. Unexpected findings in neck dissection for squamous cell carcinoma: incidence and implications. *Head Neck* 2005; **27:** 28-35.
664. Tas A, Yagiz R, Karasalihoglu AR. Thyroid gland tuberculosis with endolaryngeal extension: a case with laryngotracheal dyspnoea. *J Laryngol Otol* 2005; **119:** 54-56.
665. Tuli BS, Gupta V, Singh H, Chary G, Chand AK. Primary tuberculosis of parotid gland. *Indian journal of otolaryngology and head and neck surgery : official publication of the Association of Otolaryngologists of India* 2005; **57:** 82-83.
666. Ajay GN, Laxmikanth C, Prashanth SK. Tuberculous ulcer of tongue with oral complications of oral antituberculosis therapy. *Indian J Dent Res* 2006; **17:** 87-90.
667. Arciniegas W, Orjuela DL. [Extrapulmonary tuberculosis: a review of 102 cases in Pereira, Colombia.] *Biomedica* 2006; **26:** 71-80; discussion 81. Spanish.
668. Ebenezer J, Samuel R, Mathew GC, Koshy S, Chacko RK, Jesudason MV. Primary oral tuberculosis: report of two cases. *Indian journal of dental research : official publication of Indian Society for Dental Research* 2006; **17:** 41-44.
669. Hermani B, Sawitra D. Laryngeal tuberculosis: an important issue. *Acta Med Indones* 2006; **38:** 29-32.
670. Inoue T. [Difference in transmissibility between bronchial and laryngeal tuberculosis--a retrospective epidemiological study of TB patients newly registered in recent 19 years in Aichi Prefecture, Japan.] *Kekkaku* 2006; **81:** 419-424. Japanese.
671. Karthikeyan BV, Pradeep AR, Sharma CG. Primary tuberculous gingival enlargement: a rare entity. *J Can Dent Assoc* 2006; **72:** 645-648.
672. Kolokotronis A, Avramidou E, Zaraboukas T, Mandraveli K, Alexiou S, Antoniades D. Oral tuberculosis associated with a treatment with anti-rheumatic drugs. *J Oral Pathol Med* 2006; **35:** 123-125.
673. Lim JY, Kim KM, Choi EC, Kim YH, Kim HS, Choi HS. Current clinical propensity of laryngeal tuberculosis: review of 60 cases. *Eur Arch Otorhinolaryngol* 2006; **263:** 838-842.
674. Liu DB, Luo RZ, Zhong JW, Huang ZY, Chen Q, Zhou LF. [Diagnosis and treatment of laryngeal web in infants.] *Zhonghua Er Bi Yan Hou Tou Jing Wai Ke Za Zhi* 2006; **41:** 120-122. Chinese.
675. Lobato MN, Wang YC, Becerra JE, Simone PM, Castro KG. Improved program activities are associated with decreasing tuberculosis incidence in the United States. *Public Health Rep* 2006; **121:** 108-115.
676. Markou K, Vlachtsis K, Agathaggelidis A, Petridis D, Nikolaou A. Laryngeal tuberculosis presenting as supraglottic carcinoma: case report and literature review. *B-ent* 2006; **2:** 91-94.
677. Morales Puebla JM, Padilla Parrado M, Diaz Sastre MA *et al.* [Laryngeal tuberculosis. Incidence between 1994 and 2004.] *An Otorrinolaringol Ibero Am* 2006; **33:** 591-598. Spanish.
678. Muecke C, Isler M, Menzies D, Allard R, Tannenbaum TN, Brassard P. The use of environmental factors as adjuncts to traditional tuberculosis contact investigation. *Int J Tuberc Lung Dis* 2006; **10:** 530-535.
679. Nalini B, Vinayak S. Tuberculosis in ear, nose, and throat practice: its presentation and diagnosis. *Am J Otolaryngol* 2006; **27:** 39-45.
680. Namyslowski G, Scierski W, Polok A, Czecior E, Lange D. [Rare case of primary sinonasal tuberculosis.] *Otolaryngol Pol* 2006; **60:** 199-202. Polish.
681. Nishiike S, Nagai M, Nakagawa A *et al.* Laryngeal tuberculosis following laryngeal carcinoma. *J Laryngol Otol* 2006; **120:** 151-153.
682. Papadogeorgakis N, Mylonas AI, Kolomvos N, Angelopoulos AP. Tuberculosis in or near the major salivary glands: Report of 3 cases. *Journal of Oral and Maxillofacial Surgery* 2006; **64:** 696-700.
683. Sareen D, Sethi A, Agarwal AK. Primary tuberculosis of the tongue: A rare nodular presentation. *British Dental Journal* 2006; **200:** 321-322.
684. Sethi A, Sareen D, Sabherwal A, Malhotra V. Primary parotid tuberculosis: varied clinical presentations. *Oral Diseases* 2006; **12:** 213-215.
685. Sharma CG, Pradeep AR, Karthikeyan BV. Primary tuberculosis clinically presenting as gingival enlargement: a case report. *J Contemp Dent Pract* 2006; **7:** 108-114.
686. Sriram G. Tuberculous ulcer of tongue with oral complications of oral antituberculous therapy. *Indian J Dent Res* 2006; **17:** 202.
687. Street I, Gillett D, Sawyer A, Weighill J. Laryngeal tuberculosis: not the usual suspect. *Br J Hosp Med (Lond)* 2006; **67:** 212-213.
688. Unal M, Vayisoglu Y, Guner N, Karabacak T. Tuberculosis of the aryepiglottic fold and sinus pyriformis: a rare entity. *Mt Sinai J Med* 2006; **73:** 806-809.
689. Vishwakarma SK, Jain S, Gupta M. Primary lingual tuberculosis presenting as cold -Abscess tongue: A case report. *Indian J Otolaryngol Head Neck Surg* 2006; **58:** 87-88.
690. Zhou T, Qu JN, Xu Y, Lei PX. [Clinical manifestation and laryngoscopic characteristics of laryngeal tuberculosis.] *Zhonghua Er Bi Yan Hou Tou Jing Wai Ke Za Zhi* 2006; **41:** 247-250. Chinese.
691. Badzek S, Misir-Krpan A, Krajina Z, Radman I, Stern-Padovan R, Dotlic S. Erdheim-Chester disease and concomitant tuberculosis successfully treated with chemotherapy and long-term steroids. *Coll Antropol* 2007; **31:** 621-623.
692. Benhammou A, El Ayoubi A, Benbouzid MA, Boulaich M, Essakall L, Kzadri M. Primary tuberculosis of the parotid gland. *Archives De Pediatrie* 2007; **14:** 1206-1209.
693. Bottini DJ, Garelli A, Felici M, Galante V, Cervelli V. Primary tuberculosis of submandibular gland. *Journal of Craniofacial Surgery* 2007; **18:** 218-219.
694. Caldart AU, Adriano CF, Caldart AU, Mocellin M. Primary tuberculosis of the parotid gland. *Brazilian journal of otorhinolaryngology* 2007; **73:** 720-720.
695. Cantarella G, Pagani D, Fasano V, Scaramellini G. Glottic tuberculosis masquerading as early multifocal carcinoma. *Tumori* 2007; **93:** 302-304.
696. Dadwal M, Mohan C, Sharma DR. Secondary pharyngeal tuberculosis. *Indian J Otolaryngol Head Neck Surg* 2007; **59:** 261-263.
697. Diaz Manzano JA, Castillo Romero JL, Padilla Romero MJ, Sanchez Lainez JJ, Castillo Aguilar C, Cegarra Navarro MF. [Simultaneous pulmonar, laryngeal and lingual affectation by Mycobacterium tuberculosis.] *An Otorrinolaringol Ibero Am* 2007; **34:** 237-241. Spianish.
698. Erbaycu AE, Taymaz Z, Tuksavul F, Afrashi A, Guclu SZ. What happens when oral tuberculosis is not treated? *Monaldi archives for chest disease = Archivio Monaldi per le malattie del torace* 2007; **67:** 116-118.
699. Fernandez P, Guyot M, Lazaro E, Viallard JF, Allard M, Ducassou D. Systemic tuberculosis presenting as an epiglottic mass detected on F-18 FDG PET/CT. *Clin Nucl Med* 2007; **32:** 719-724.
700. Garg RK, Singhal P. Primary tuberculosis of the tongue: a case report. *J Contemp Dent Pract* 2007; **8:** 74-80.
701. Gupta MK, Singh M. Primary tuberculosis of mandible. *Indian Pediatrics* 2007; **44:** 53-54.
702. Gupta PP, Fotedar S, Agarwal D, Sansanwal P. Primary tuberculous glossitis in an immunocompetent patient. *Hong Kong medical journal = Xianggang yi xue za zhi* 2007; **13:** 330-331.
703. Hemmaoui B, Bouayti B, Errami N *et al.* [Laryngeal tuberculosis: a case report.] *Rev Laryngol Otol Rhinol (Bord)* 2007; **128:** 93-96. French.
704. Ito M, Yasuo M, Nakamura M, Tsushima K, Yamazaki Y, Kubo K. [A case of tuberculous bronchial stenosis, diagnosed after 50 years of pulmonary and laryngeal tuberculosis.] *Nihon Kokyuki Gakkai Zasshi* 2007; **45:** 87-90. Japanese.
705. Karbach J, Thal SC, Weber A *et al.* Swelling of the buccal cheek: An unusual presentation of primary tuberculosis. *Journal of Oral and Maxillofacial Surgery* 2007; **65:** 2108-2111.
706. Keberle M, Robinson S. Physiologic and pathologic calcifications and ossifications in the face and neck. *Eur Radiol* 2007; **17:** 2103-2111.
707. Koc O, Paksoy Y, Erayman I, Kivrak AS, Arbag H. Role of diffusion weighted MR in the discrimination diagnosis of the cystic and/or necrotic head and neck lesions. *Eur J Radiol* 2007; **62:** 205-213.
708. Mahajan S, Srikant N, George T. Atypical presentation of oral tuberculosis ulcer. *N Y State Dent J* 2007; **73:** 48-50.
709. Menon K, Bem C, Gouldesbrough D, Strachan DR. A clinical review of 128 cases of head and neck tuberculosis presenting over a 10-year period in Bradford, UK. *J Laryngol Otol* 2007; **121:** 362-368.
710. Nohrstrom E, Kentala E, Kuusela P, Mattila PS. Tuberculosis of the head and neck in Finland. *Acta Otolaryngol* 2007; **127:** 770-774.
711. Ozbey SB, Kasapoglu F, Helvaci S, Aydin O. Case report: Tuberculosis of parotid gland. *Mikrobiyoloji Bulteni* 2007; **41:** 139-143.
712. Rodrigues G, Carnelio S, Valliathan M. Primary isolated gingival tuberculosis. *Braz J Infect Dis* 2007; **11:** 172-173.
713. Sa LC, Meirelles RC, Atherino CC, Fernandes JR, Ferraz FR. Laryngo-pharyngeal Tuberculosis. *Braz J Otorhinolaryngol* 2007; **73:** 862-866.
714. Saarinen RT, Kolho K-L, Pitkaranta A. Cases presenting as parotid abscesses in children. *International Journal of Pediatric Otorhinolaryngology* 2007; **71:** 897-901.
715. Satoh S, Inoue A, Kidera K, Kuratomi Y, Inokuchi A. [A case of follicular carcinoma of thyroid gland with concurrent tuberculous lymphadenitises.] *Nihon Jibiinkoka Gakkai Kaiho* 2007; **110:** 20-23. Japanese.
716. Seeley M, Waterhouse D, Shetty S, Gathercole J, Seeley C. Two cases of parotid tuberculosis. *The New Zealand medical journal* 2007; **120:** U2869-U2869.
717. Trivedi A, Patel J, Kalola J. Ulcer of the tongue as a presenting feature of pulmonary tuberculosis. *Indian J Otolaryngol Head Neck Surg* 2007; **59:** 166-167.
718. Vidal M, Delevaux I, Andre M *et al.* [Lingual tuberculosis revealing disseminated tuberculosis.] *Rev Med Interne* 2007; **28:** 124-126. French.
719. Wang CC, Lin CC, Wang CP, Liu SA, Jiang RS. Laryngeal tuberculosis: a review of 26 cases. *Otolaryngol Head Neck Surg* 2007; **137:** 582-588.
720. Wang CY, Ling L, Zhou SH, Wang SQ. [Analysis of misdiagnosis of laryngeal tuberculosis: a report of 16 cases.] *Zhonghua Jie He He Hu Xi Za Zhi* 2007; **30:** 424-426. Chinese.
721. Wang PH, Wang HC, Cheng PW, Cheng SL, Tsai CC. Hoarseness in asthmatic patients: the side effect of inhaled steroid or not? *J Asthma* 2007; **44:** 823-826.
722. Zirlik S, Fuchs FF, Hahn EG, Zenk J, Wiest GH. [Pseudotumoral laryngeal tuberculosis.] *Med Klin (Munich)* 2007; **102:** 393-395. German.
723. Acharya VK, Sahoo R, Sreedharan S, Anand R, Pathak R. Rare causes of voice hoarseness: a case report. *Nepal Med Coll J* 2008; **10:** 141-143.
724. Ahmad T, Naeem M, Ahmad S, Samad A, Nasir A. Fine needle aspiration cytology (FNAC) and neck swellings in the surgical outpatient. *J Ayub Med Coll Abbottabad* 2008; **20:** 30-32.
725. Archontaki M, Stamou AK, Hajiioannou JK, Kalomenopoulou M, Kyrmizakis DE. Intraparotid lymph node tuberculosis. *B-ent* 2008; **4:** 35-37.
726. Birkent H, Karahatay S, Akcam T, Durmaz A, Ongoru O. Primary parotid tuberculosis mimicking parotid neoplasm: a case report. *Journal of medical case reports* 2008; **2:** 62-62.
727. Chakravarti A, Pal S, Sahni JK. Primary tuberculosis of tonsil and posterior oropharyngeal wall. *Indian J Tuberc* 2008; **55:** 48-50.
728. Dinkar AD, Prabhudessai V. Primary tuberculous osteomyelitis of the mandible: a case report. *Dentomaxillofacial Radiology* 2008; **37:** 415-420.
729. Dixit R, Sharma S, Nuwal P. Tuberculosis of oral cavity. *Indian J Tuberc* 2008; **55:** 51-53.
730. Hale RG, Tucker DI. Head and neck manifestations of tuberculosis. *Oral Maxillofac Surg Clin North Am* 2008; **20:** 635-642.
731. Kant S, Verma SK, Sanjay. Isolated tonsil tuberculosis. *Lung India* 2008; **25:** 163-164.
732. Kharoubi S. [Pharyngeal tuberculosis.] *Ann Otolaryngol Chir Cervicofac* 2008; **125:** 218-223. French.
733. Koffi SK, Kouassi AB, Faye-Kette H, Kouassi-M'bengue A, Ahui JM, Aka-Danguy E. [Tuberculosis of the oral mucosa in an HIV/AIDS patient.] *Med Mal Infect* 2008; **38:** 167-168. French.
734. Mizutari K, Tsunoda K, Matsunaga T, Masuda K, Fujii R, Fujii M. Oropharyngeal tuberculosis. *Internal medicine journal* 2008; **38:** 449-450.
735. Niizuma K, Saito M. [Case report of tuberculous retropharyngeal abscess.] *Kekkaku* 2008; **83:** 393-397. Japanese.
736. Roberts DS, Dowdall JR, Winter L, Sulis CA, Grillone GA, Grundfast KM. Cervical tuberculosis: a decision tree for protecting healthcare workers. *Laryngoscope* 2008; **118:** 1345-1349.
737. Santosh UP, Vinay B. Tuberculosis of tonsil associated with pulmonary foci. *Indian J Otolaryngol Head Neck Surg* 2008; **60:** 263-265.
738. Saroul N, Vellin JF, Baud O, Nohra O, Kemeny JL, Gilain L. [Epilaryngeal tuberculosis: epidemiologic, clinical and healthcare considerations.] *Ann Otolaryngol Chir Cervicofac* 2008; **125:** 155-159. French.
739. Sathish C, Nyamannawar BM, Mohanty S, Correa MM, Das K. Atypical thyroglossal duct anomalies. *Int J Pediatr Otorhinolaryngol* 2008; **72:** 1353-1357.
740. Schmid D, Fretz R, Kuo HW *et al.* An outbreak of multidrug-resistant tuberculosis among refugees in Austria, 2005-2006. *Int J Tuberc Lung Dis* 2008; **12:** 1190-1195.
741. Sharma AB, Laishram DK, Sarma B. Primary tuberculosis of tongue. *Indian Journal of Pathology and Microbiology* 2008; **51:** 65-66.
742. Smolka W, Burger H, Iizuka T, Smolka K. Primary tuberculosis of the oral cavity in an elderly nonimmunosuppressed patient: case report and review of the literature. *Arch Otolaryngol Head Neck Surg* 2008; **134:** 1107-1109.
743. Topak M, Oysu C, Yelken K, Sahin-Yilmaz A, Kulekci M. Laryngeal involvement in patients with active pulmonary tuberculosis. *Eur Arch Otorhinolaryngol* 2008; **265:** 327-330.
744. Tovaru S, Costache M, Sardella A. Primary oral tuberculosis: a case series from Bucharest, Romania. *Oral Surg Oral Med Oral Pathol Oral Radiol Endod* 2008; **105:** e41-45.
745. Uslu C, Oysu C, Uklumen B. Tuberculosis of the epiglottis: a case report. *Eur Arch Otorhinolaryngol* 2008; **265:** 599-601.
746. Willenborg KM, Goetz F, Klein R, Lenarz T, Stoever T. Tuberculosis in the parotid gland in HIV-infection. *Laryngo-Rhino-Otologie* 2008; **87:** 420-422.
747. Yelken K, Guven M, Topak M, Gultekin E, Turan F. Effects of antituberculosis treatment on self assessment, perceptual analysis and acoustic analysis of voice quality in laryngeal tuberculosis patients. *J Laryngol Otol* 2008; **122:** 378-382.
748. Aerts S, Gypen BJ, Van Hee R, Bomans P. Tuberculosis of the thyroid gland. A case report. *Acta Chir Belg* 2009; **109:** 805-807.
749. Andratschke M, Nerlich AG, Hagedorn H. [Florid tuberculosis of the paranasal sinuses.] *Hno* 2009; **57:** 1209-1212. German.
750. Bal MS, Bharti V, Singh A. Gingival tuberculosis. *Indian Journal of Pathology and Microbiology* 2009; **52:** 442-443.
751. Benmansour N, El Hord S, Oudidi A, Benjelloun MC, El Alami MN. Primary tuberculosis of the tongue. *Journal of Otolaryngology-Head & Neck Surgery* 2009; **38:** E73-E75.
752. Bhat VK, Latha P, Upadhya D, Hegde J. Clinicopathological review of tubercular laryngitis in 32 cases of pulmonary Kochs. *Am J Otolaryngol* 2009; **30:** 327-330.
753. Caruso G, Passali FM, Salerni L, Molinaro G, Messina M. Head and neck mycobacterial infections in pediatric patients. *Int J Pediatr Otorhinolaryngol* 2009; **73 Suppl 1:** S38-41.
754. Dokuzlar U, Erisen L, Kasapoglu F *et al.* Unexpected findings in the neck dissection specimens for primary head and neck squamous cell carcinoma. *Kulak Burun Bogaz Ihtis Derg* 2009; **19:** 9-15.
755. El Ayoubi A, Benhammou A, El Ayoubi F *et al.* [Primary extranodal ENT tuberculosis]. *Annales d'oto-laryngologie et de chirurgie cervico faciale : bulletin de la Societe d'oto-laryngologie des hopitaux de Paris* 2009; **126:** 208-215. French.
756. Furugen M, Nakamura H, Tamaki Y *et al.* [Tuberculosis of the tongue initially suspected of tongue cancer: a case report--including the search for recent 16 cases in Japan.] *Kekkaku : [Tuberculosis]* 2009; **84:** 605-610. Japanese.
757. Gregg KK, Detjen AK, Goussard P, Gie R. Laryngeal involvement in two severe cases of childhood tuberculosis. *Pediatr Infect Dis J* 2009; **28:** 1136-1138.
758. Jain S, Vipin B, Khurana P. Gingival tuberculosis. *J Indian Soc Periodontol* 2009; **13:** 106-108.
759. Khan NU, Wallis S, Siddiqui N. Laryngeal tuberculosis: a diagnosis not to be missed. *BMJ Case Rep* 2009; **2009**.
760. Kilic A, Guel U, Goenuel M, Soylu S, Cakmak SK, Demiriz M. Orificial tuberculosis of the lip: a case report and review of the literature. *International Journal of Dermatology* 2009; **48:** 178-180.
761. Maharjan M, Hirachan S, Kafle PK *et al.* Incidence of tuberculosis in enlarged neck nodes, our experience. *Kathmandu Univ Med J (KUMJ)* 2009; **7:** 54-58.
762. Mascarenhas S, Tuffin JR, Hassan I. Tuberculous submasseteric abscess: case report. *Br J Oral Maxillofac Surg* 2009; **47:** 566-568.
763. Nadour K, Hemmaoui B, Errami N *et al.* [Tonsillitis tuberculous simulating tonsil cancer: case report.] *Rev Laryngol Otol Rhinol (Bord)* 2009; **130:** 301-303. French.
764. Pio A. The infectiousness of laryngeal tuberculosis. *Int J Tuberc Lung Dis* 2009; **13:** 670.
765. Ramesh V. Orofacial Granulomatosis due to Tuberculosis. *Pediatr Dermatol* 2009; **26:** 108-109.
766. Rieder HL. The infectiousness of laryngeal tuberculosis: appropriate public health action based on false premises. *Int J Tuberc Lung Dis* 2009; **13:** 4-5.
767. Vilar FC, de Souza A, Moya MJ *et al.* Atypical oral lesion in a patient with pulmonary tuberculosis. *International Journal of Dermatology* 2009; **48:** 910-912.
768. Wang WC, Chen JY, Chen YK, Lin LM. Tuberculosis of the head and neck: a review of 20 cases. *Oral Surg Oral Med Oral Pathol Oral Radiol Endod* 2009; **107:** 381-386.
769. Yoruk O, Fidan V, Sutbeyaz Y. Hearing loss unusually caused by tubercular retropharyngeal abscess. *J Craniofac Surg* 2009; **20:** 955-957.
770. Zhao N, Sun Y, Sun Z. [Clinical analysis of the diagnosis of laryngeal tuberculosis.] *Lin Chung Er Bi Yan Hou Tou Jing Wai Ke Za Zhi* 2009; **23:** 261-263. Chinese.
771. Al Bisher H. A rare case of primary tuberculosis infection with concurrent pleomorphic adenoma of the parotid gland. *Infectious disease reports* 2010; **2:** e6-e6.
772. Al-Zahid S, Singh V. Tuberculous cervical lymphadenitis in a patient with laryngeal carcinoma. *J Laryngol Otol* 2010; **124:** 90-92.
773. Bagga P, Pandey P, Shahi M, Mittal A, Mehta V, Ganju A. Parotid gland tuberculosis diagnosed on FNAC: a case report. *Cytopathology* 2010; **21:** 127-129.
774. Barouta G, Karapetsa M, Kostopoulou E, Alexiou I, Koukoulis G, Sakkas LI. Oral tuberculosis in a patient with rheumatoid arthritis after long treatment with methotrexate and adalimumab. *J Clin Rheumatol* 2010; **16:** 330-331.
775. Baxi S, Jha S. Primary laryngeal tuberculosis--a rare entity. *J Indian Med Assoc* 2010; **108:** 178-179.
776. Bharatha A, Bartlett ES, Eugene Y. Case 154: pharyngeal and retropharyngeal tuberculosis with nodal disease. *Radiology* 2010; **254:** 629-632.
777. Cherkaoui A, Oudidi A, El Alami M. [Laryngeal tuberculosis.] *J Otolaryngol Head Neck Surg* 2010; **39:** 35-38. French.
778. Edizer DT, Karaman E, Mercan H, Alimoglu Y, Esen T, Cansiz H. Primary tuberculosis involving epiglottis: a rare case report. *Dysphagia* 2010; **25:** 258-260.
779. El Kettani NE, El Hassani M, Chakir N, Jiddane M. Primary laryngeal tuberculosis mimicking laryngeal carcinoma: CT scan features. *Indian J Radiol Imaging* 2010; **20:** 11-12.
780. Garg R, Verma SK, Mehra S, Srivastawa AN. Parotid tuberculosis. *Lung India : official organ of Indian Chest Society* 2010; **27:** 253-255.
781. Gill JS, Sandhu S, Gill S. Primary tuberculosis masquerading as gingival enlargement. *Br Dent J* 2010; **208:** 343-345.
782. Ito K, Morooka M, Kubota K. 18F-FDG PET/CT findings of pharyngeal tuberculosis. *Ann Nucl Med* 2010; **24:** 493-496.
783. Jadia S, Chauhan AN, Hazari RS, Maurya AK, Biswas R. An unusual cause of recurrent tonsillitis. *BMJ Case Rep* 2010; **2010:** 2561.
784. Kakisi OK, Kechagia AS, Kakisis IK, Rafailidis PI, Falagas ME. Tuberculosis of the oral cavity: a systematic review. *Eur J Oral Sci* 2010; **118:** 103-109.
785. Kumar S, Sen R, Rawal A, Dahiya RS, Dalal N, Kaushik S. Primary lingual tuberculosis in immunocompetent patient: a case report. *Head Neck Pathol* 2010; **4:** 178-180.
786. Ling L, Zhou SH, Wang SQ. Changing trends in the clinical features of laryngeal tuberculosis: a report of 19 cases. *Int J Infect Dis* 2010; **14:** e230-235.
787. Liu SC, Wang CH. Multiple head and neck tuberculosis granulomas in a patient with thymoma and immunodeficiency (Good's syndrome). *Otolaryngol Head Neck Surg* 2010; **142:** 454-455.
788. Maragou C, Theologie-Lygidakis N, Ioannidis P *et al.* Primary tooth abscess caused by Mycobacterium bovis in an immunocompetent child. *Eur J Pediatr* 2010; **169:** 1143-1145.
789. Nagalakshmi V, Nagabhushana D, Aara A. Primary tuberculous lymphadenitis: A case report. *Clin Cosmet Investig Dent* 2010; **2:** 21-25.
790. Parab SR, Khan MM, Ghaisas VS. Simultaneous involvement of larynx and middle ear in pulmonary tuberculosis. *Laryngoscope* 2010; **120:** 1892-1894.
791. Ruas AC, Rolla VC, de Araujo-Melo MH, Moreira JS, Valete-Rosalino CM. Vocal quality of patients treated for laryngeal tuberculosis, before and after speech therapy. *J Laryngol Otol* 2010; **124:** 1153-1157.
792. Vaid S, Lee YY, Rawat S, Luthra A, Shah D, Ahuja AT. Tuberculosis in the head and neck--a forgotten differential diagnosis. *Clin Radiol* 2010; **65:** 73-81.
793. Vayisoglu Y, Unal M, Ozcan C, Gorur K, Horasan ES, Sevuk L. [Lesions of tuberculosis in the head and neck region: a retrospective analysis of 48 cases.] *Kulak Burun Bogaz Ihtis Derg* 2010; **20:** 57-63. Turkish.
794. Venkatanarasimha N. Re: tuberculosis in the head and neck--a forgotten differential diagnosis. *Clin Radiol* 2010; **65:** 769; author reply 769-770.
795. Zub K, Zatonski T, Krecicki T. [Laryngeal tuberculosis in the patients of otholaryngology department--case reports.] *Otolaryngol Pol* 2010; **64:** 177-179. Polish.
796. Adhikari P, Sinha B, Baskota D. Comparison of fine needle aspiration cytology and histopathology in diagnosing cervical lymphadenopathies. *Australas Med J* 2011; **4:** 97-99.
797. Al-Hazmi WA. Tuberculosis of the Malar and Zygomatic bone: A case report". *Int J Health Sci (Qassim)* 2011; **5:** 197-200.
798. Al-Rikabi AC, Arafah MAR. Tuberculosis of the tongue clinically masquerading as a neoplasm: a case report and literature review. *Oman medical journal* 2011; **26:** 267-268.
799. Bairagya TD, Das SK, Barman DC, Bhattacharya S. Primary oral tuberculosis. *N Z Med J* 2011; **124:** 93-95.
800. Dadwal M. Primary Submandibular Tuberculosis: An Unusual Cause of Submandibular Salivary Gland Enlargement. *Indian Journal of Otolaryngology and Head & Neck Surgery* 2011; **63:** 298-299.
801. Diom ES, Ndiaye C, Djafarou AB *et al.* A case of cervical Pott's disease revealed by parapharyngeal abscess. *Eur Ann Otorhinolaryngol Head Neck Dis* 2011; **128:** 151-153.
802. El Beltagi AH, Khera PS, Alrabiah L, Al Shammari NF. Case Report: Acute tuberculous laryngitis presenting as acute epiglottitis. *Indian J Radiol Imaging* 2011; **21:** 284-286.
803. Gharebaghi N, Monsouri SA, Darazam IA, Mansouri D, Sajadi MM, Mansouri N. A 40-Year-old Man With Tongue Lesions. *Clinical Infectious Diseases* 2011; **52:** 1276-1277.
804. Gonzalez N, Charlone G, Sanguinetti A. [Laryngeal and lung tuberculosis: a case presentation.] *Acta Gastroenterol Latinoam* 2011; **41:** 52-54. Spanish.
805. Gupta G, Khattak BP, Agrawal V. Primary gingival tuberculosis: A rare clinical entity. *Contemp Clin Dent* 2011; **2:** 31-33.
806. Huon LK, Fang TY. Primary laryngeal tuberculosis. *J Formos Med Assoc* 2011; **110:** 792-793.
807. Jaiswal R, Singh A, Badni M, Singh P. Oral tuberculosis involving maxillary gingiva. *Natl J Maxillofac Surg* 2011; **2:** 175-176.
808. Kamala R, Sinha A, Srivastava A, Srivastava S. Primary tuberculosis of the oral cavity. *Indian J Dent Res* 2011; **22:** 835-838.
809. Kannan S, Thakkar P, Dcruz AK. Tuberculosis masquerading as oral malignancy. *Indian J Med Paediatr Oncol* 2011; **32:** 180-182.
810. Kumar V, Singh AP, Meher R, Raj A. Primary tuberculosis of oral cavity: a rare entity revisited. *Indian J Pediatr* 2011; **78:** 354-356.
811. Markowski J, Witkowska M, Gierek T *et al.* [Head and neck tuberculosis - still current problem in ENT practice.] *Otolaryngol Pol* 2011; **65:** 272-275. Polish.
812. Masterson L, Srouji I, Kent R, Bath AP. Nasal tuberculosis--an update of current clinical and laboratory investigation. *J Laryngol Otol* 2011; **125:** 210-213.
813. McAllister KA, MacGregor FB. Diagnosis of tuberculosis in the head and neck. *J Laryngol Otol* 2011; **125:** 603-607.
814. Nanda KD, Mehta A, Marwaha M, Kalra M, Nanda J. A disguised tuberculosis in oral buccal mucosa. *Dent Res J (Isfahan)* 2011; **8:** 154-159.
815. Naseem K, Zafar MJ, Rabbani MZ. Lingual Tuberculosis. *Jcpsp-Journal of the College of Physicians and Surgeons Pakistan* 2011; **21:** 704-705.
816. Sansare K, Gupta A, Khanna V, Karjodkar F. Oral tuberculosis: unusual radiographic findings. *Dentomaxillofac Radiol* 2011; **40:** 251-256.
817. Schrock A, Goke F, Jakob M *et al.* [Initial diagnosis of head and neck tuberculosis.] *Laryngorhinootologie* 2011; **90:** 604-608. German.
818. Singhaniya SB, Barpande SR, Bhavthankar JD. Oral tuberculosis in an asymptomatic pulmonary tuberculosis. *Oral Surg Oral Med Oral Pathol Oral Radiol Endod* 2011; **111:** e8-10.
819. Sun WL, Xu KL, Chen LL, Yu ZS. Tuberculosis cutis orificialis with both gingival involvement and underlying pulmonary tuberculosis. *Aust Dent J* 2011; **56:** 216-220.
820. Tauro LF, George C, Kamath A, Swethadri G, Gatty R. Primary tuberculosis of submandibular salivary gland. *Journal of global infectious diseases* 2011; **3:** 82-85.
821. Thakur J, Thakur A, Mohindroo N, Mohindroo S, Sharma D. Bilateral parotid tuberculosis. *Journal of global infectious diseases* 2011; **3:** 296-299.
822. Upadhyay S, Sharma A, Tuljapurkar V, Dabholkar JP. Primary tuberculous osteomyelitis of the mandible mimicking a parotid fistula. *Brazilian Journal of Otorhinolaryngology* 2011; **77:** 403-403.
823. Wang H, Bai Z, Wang K *et al.* [Secondary laryngeal tuberculosis at high altitudes of Tibet.] *Lin Chung Er Bi Yan Hou Tou Jing Wai Ke Za Zhi* 2011; **25:** 977-981. Chinese.
824. Aggarwal P, Saxena S, Reddy V, Sharma P, Aggarwal V. Tuberculosis, the culprit behind nonhealing oral lesions: report of two cases. *Indian J Med Sci* 2012; **66:** 280-285.
825. Ariel BM, Nasyrov RA, Baiburina NA, Plechkov RE. [Tuberculosis of the pharyngeal tonsil in a child.] *Arkh Patol* 2012; **74:** 35-38. Russian.
826. Bagga P, Dewan A, Agarwal P, Garg C, Datta NR. Oral tuberculosis following successful treatment of oral malignancy. *J Cancer Res Ther* 2012; **8:** 650-651.
827. Bakir S, Tanriverdi MH, Gun R *et al.* Deep neck space infections: a retrospective review of 173 cases. *Am J Otolaryngol* 2012; **33:** 56-63.
828. Burger MS, Abraham-Inpijn L, Vissink A. [Tuberculosis in the dental office. Epidemiology, clinical view and prevention.] *Ned Tijdschr Tandheelkd* 2012; **119:** 175-182. Dutch.
829. Chauhan V, Mahesh DM, Panda P, Mahajan S, Thakur S. Tuberculosis cutis orificialis (TBCO): a rare manifestation of tuberculosis. *The Journal of the Association of Physicians of India* 2012; **60:** 126-127.
830. Chen H, Thornley P. Laryngeal tuberculosis: A case of a non-healing laryngeal lesion. *Australas Med J* 2012; **5:** 175-177.
831. Christoforidou A, Metallidis S, Kollaras P *et al.* Tuberculous retropharyngeal abscess as a cause of oropharyngeal dysphagia. *Am J Otolaryngol* 2012; **33:** 272-274.
832. Gandhi S, Kulkarni S, Mishra P, Thekedar P. Tuberculosis of larynx revisited: a report on clinical characteristics in 10 cases. *Indian J Otolaryngol Head Neck Surg* 2012; **64:** 244-247.
833. Guinchard AC, Pasche P. [Peripheral tuberculous lymphadenitis: diagnosis and management.] *Rev Med Suisse* 2012; **8:** 1860-1862, 1864-1865. French.
834. Hussaini J, Mutusamy S, Omar R, Rajagopalan R, Narayanan P. Base of tongue tuberculosis: a case report. *Acta Med Iran* 2012; **50:** 151-152.
835. Javali MA, Patil V, Ayesha H. Periodontal disease as the initial oral manifestation of abdominal tuberculosis. *Dent Res J (Isfahan)* 2012; **9:** 634-637.
836. Karagozoglu KH, Eerenstein SE, van der Waal I. [A man from Africa with a swelling in the head and neck region.] *Ned Tijdschr Tandheelkd* 2012; **119:** 18-20. Dutch.
837. Kiakojuri K, Hasanjani Roushan MR. Laryngeal tuberculosis without pulmonary involvement. *Caspian J Intern Med* 2012; **3:** 397-399.
838. Kitahara K, Yano Y, Mori M, Yokota S. Laryngeal tuberculosis: a diagnosis that should be remembered. *Intern Med* 2012; **51:** 2841-2842.
839. Moffett KS. Pediatric infectious disease: unusual head and neck infections. *Oral Maxillofac Surg Clin North Am* 2012; **24:** 469-486.
840. Mohanapriya T, Singh KB, Arulappan T, Dhanasekar T. Lingual tuberculosis. *Indian J Tuberc* 2012; **59:** 39-41.
841. Obourn C, Aynehchi B, Bentsianov B. Atypical presentation of laryngeal tuberculosis in a pediatric patient. *Int J Pediatr Otorhinolaryngol* 2012; **76:** 752-753.
842. Ozcan C, Vaysoglu Y, Gucluturk T, Apa DD, Gorur K. Nasopharyngeal tuberculosis presenting as massive cervical lymphadenopathy and hearing loss. *J Craniofac Surg* 2012; **23:** e341-343.
843. Pasticci MB, Floridi P, Schiaroli E *et al.* Lingual tuberculosis: a rare disease in Western countries. *New Microbiologica* 2012; **35:** 233-237.
844. Patankar SS, Chandorkar SS, Garg A. Parotid Gland Tuberculosis: A Case Report. *Indian Journal of Surgery* 2012; **74:** 179-180.
845. Peck MT, Stephen LX, Marnewick J, Majeed A. Palatal ulceration as the first sign of pulmonary tuberculosis: a case report. *Trop Doct* 2012; **42:** 52-53.
846. Prasad P, Bhardwaj M. Primary tuberculosis of tonsils: a case report. *Case Rep Med* 2012; **2012:** 120382.
847. Rathod GB, Parmar P. Fine needle aspiration cytology of swellings of head and neck region. *Indian J Med Sci* 2012; **66:** 49-54.
848. Rout MR, Moharana PR. Tuberculosis of larynx: a case report. *Indian J Tuberc* 2012; **59:** 231-234.
849. Suhail A, Ahmed MS, Sobani ZU, Ghaffar S. Laryngeal tuberculosis presenting as laryngeal carcinoma. *J Pak Med Assoc* 2012; **62:** 167-168.
850. Tanwar R, Iyengar AR, Nagesh KS, Jhamb P. Primary tuberculosis: an unusual finding in the oral cavity. *Oral Health Dent Manag* 2012; **11:** 23-28.
851. Wu K-C, Chen B-N. Mycobacterial tuberculosis superimposed on a Warthin tumor. *Ear, nose, & throat journal* 2012; **91:** E4-6.
852. Yadav SP, Agrawal A, Gulia JS, Singh S, Gupta A, Panchal V. Tuberculoma of the tongue presenting as hemimacroglossia. *Case Rep Med* 2012; **2012:** 548350.
853. Zaki SA, Bhongade S, Vartak SS. Perforation of the hard palate due to tuberculosis. *Dent Res J (Isfahan)* 2012; **9:** 804-806.
854. Balikci HH, Gurdal MM, Ozkul MH *et al.* Neck masses: diagnostic analysis of 630 cases in Turkish population. *Eur Arch Otorhinolaryngol* 2013; **270:** 2953-2958.
855. Battista G, Lo Russo L, Padovano Di Leva A *et al.* Oral tuberculosis: a tongue case report. *Minerva Stomatol* 2013.
856. Beogo R, Birba NE, Coulibaly TA, Traore I, Ouoba K. [Presentations of tuberculous adenitis of the head and neck at the University Hospital of Bobo-Dioulasso, Burkina Faso.] *Pan Afr Med J* 2013; **15:** 131. French.
857. Dogra SS, Chander B, Krishna M. Tuberculosis of oral cavity: a series of one primary and three secondary cases. *Indian J Otolaryngol Head Neck Surg* 2013; **65:** 275-279.
858. Esposito S, Giannini A, Biondetti P *et al.* Subcutaneous emphysema as the first relevant clinical sign of complicated tubercular lymph node disease in a child. *BMC Infect Dis* 2013; **13:** 461.
859. Fazal Iw, Habib Ur R, Ahmad I. Extrapulmonary tuberculosis in patients with cervical lymphadenopathy. *J Pak Med Assoc* 2013; **63:** 1094-1097.
860. Gelli V, Mutalik SS, Mutalik VS, Manyam R. Primary gingival tuberculosis diagnosis: a difficult endeavor. *J Contemp Dent Pract* 2013; **14:** 137-139.
861. Godoy P, Torres J, Otal J, Gort A, Bach P, Falguera M. [Contact investigation according to concentric circles in a case of laryngeal tuberculosis.] *Gac Sanit* 2013; **27:** 279-281. Spanish.
862. Halim MS, Ahmed SQ, Junaid M, Bashir MR. Tuberculosis verrucosa cutis in a patient with keloid over ear lobule. *BMJ Case Rep* 2013; **2013**.
863. Hasibi M, Yazdani N, Asadollahi M, Sharafi M, Dehghan Manshadi SA. Clinical features of laryngeal tuberculosis in Iran. *Acta Med Iran* 2013; **51:** 638-641.
864. Ishinaga H, Hamaguchi N, Suzuki H *et al.* [Case of papillary carcinoma of the thyroid gland with concurrent tuberculous lymphadenitis.] *Nihon Jibiinkoka Gakkai Kaiho* 2013; **116:** 1315-1319. Japanese.
865. Kannaperuman J, Natarajarathinam G, Rao AV, Palanimuthu S. Primary tuberculous osteomyelitis of the mandible: A rare case report. *Dental research journal* 2013; **10:** 283-286.
866. Kumar BN, Manish N, Mallikarjuna R, Sundaresh KJ. Primary oral tuberculosis in a sexagenarian female. *BMJ Case Rep* 2013; **2013**.
867. Liu W, Li X, Yin J, Li X, Wang X. [Diagnostic value of (18)F-FDG PET/CT in extrapulmonary tuberculosis.] *Nan Fang Yi Ke Da Xue Xue Bao* 2013; **33:** 1083-1086. Chinese.
868. Luksic B, Kljajic Z, Roje Z *et al.* Direct molecular detection of Mycobacterium tuberculosis suspected to be the specific infection in a case of recurrent tonsillitis. *J Infect Chemother* 2013; **19:** 1185-1187.
869. Maclean KA, Becker AK, Chang SD, Harris AC. Extrapulmonary tuberculosis: imaging features beyond the chest. *Can Assoc Radiol J* 2013; **64:** 319-324.
870. Nagaraj V, Sashykumar S, Viswanathan S, Kumar S. Multiple oral ulcers leading to diagnosis of pulmonary tuberculosis. *Eur J Dent* 2013; **7:** 243-245.
871. Patel AB, Hinni ML. Tuberculous retropharyngeal abscess presenting with symptoms of obstructive sleep apnea. *Eur Arch Otorhinolaryngol* 2013; **270:** 371-374.
872. Santiago RA, Gueiros LA, Porter SR, Gomes VB, Ferrer I, Leao JC. Prevalence of oral lesions in Brazilian patients with tuberculosis. *Indian J Dent Res* 2013; **24:** 245-248.
873. Sawada N, Inokuchi G, Komatsu H, Kurakawa S, Tada K, Kumoi K. Nasopharyngeal tuberculosis. *J Infect Chemother* 2013; **19:** 1158-1160.
874. Sharma P, Saxena S, Aggarwal P, Reddy V. Tuberculosis of odontogenic cyst. *Indian J Tuberc* 2013; **60:** 50-54.
875. Takagi A, Nagayasu F, Sugama Y, Shiraishi S. [Primary nasopharyngeal tuberculosis.] *Kekkaku* 2013; **88:** 485-489. Japanese.
876. Ulusan M, Abul Y, Bakir S. Mycobacterium Tuberculosis Infection within a Warthin Tumor: A Case Report and Literature Review. *North American journal of medical sciences* 2013; **5:** 617-619.
877. Verma S, Mohan RP, Singh U, Agarwal N. Primary oral tuberculosis. *BMJ Case Rep* 2013; **2013**.
878. Wang SY, Zhu JX. [Primary mucosal tuberculosis of head and neck region: a clinicopathologic analysis of 47 cases.] *Zhonghua Bing Li Xue Za Zhi* 2013; **42:** 683-686. Chinese.
879. Zhao YM, Shi BY. [Analysis of the misdiagnosed patients with laryngeal tuberculosis: report of three cases.] *Zhonghua Er Bi Yan Hou Tou Jing Wai Ke Za Zhi* 2013; **48:** 773-774. Chinese.
880. Ziad T, Nouri H, Adny A, Rochdi Y, Aderdour L, Raji A. [Acute miliary tuberculosis or Isambert disease: a case report.] *Arch Pediatr* 2013; **20:** 41-43. French.
881. Benwill JL, Sarria JC. Laryngeal tuberculosis in the United States of America: a forgotten disease. *Scand J Infect Dis* 2014; **46:** 241-249.
882. Chang JH, Kim JH, Kang JW, Kim JH. Oropharyngeal and miliary pulmonary tuberculosis without respiratory symptom. *Southeast Asian J Trop Med Public Health* 2014; **45:** 869-873.
883. Chaudhary N, D KG, Choudhary SR, Dawson L. Primary tuberculosis of the cheek: a common disease with a rare presentation. *Malays J Med Sci* 2014; **21:** 66-68.
884. Cruz S, Ribeiro A, Trigueiros N, Rodrigues e Rodrigues M. Laryngeal tuberculosis: a diagnosis not to be overlooked. *Eur Ann Otorhinolaryngol Head Neck Dis* 2014; **131:** 325-326.
885. Dhuvad J, Patel B, Madan S, Dhuvad M. Orofacial tubercular lesions. *Indian J Tuberc* 2014; **61:** 325-330.
886. El Ayoubi F, Chariba I, El Ayoubi A, Chariba S, Essakalli L. Primary tuberculosis of the larynx. *Eur Ann Otorhinolaryngol Head Neck Dis* 2014; **131:** 361-364.
887. Fei B, Wu Z, Min K, Zhang J, Ding C, Wu H. Interferon-gamma release assay in the diagnosis of laryngeal tuberculosis. *Acta Otolaryngol* 2014; **134:** 314-317.
888. Fsadni P, Fsadni C, Caruana Montaldo B. Primary laryngeal tuberculosis: An unusual cause of hoarseness. *Ear Nose Throat J* 2014; **93:** E15-17.
889. Fukui S, Takizawa Y, Kubota N, Okamoto T, Hishima T. Tuberculous lymphadenitis and the appearance of Behcet's disease-like symptoms. *Intern Med* 2014; **53:** 805-808.
890. Gemaque K, Giacomelli Nascimento G, Cintra Junqueira JL, Cavalcanti de Araujo V, Furuse C. Prevalence of oral lesions in hospitalized patients with infectious diseases in northern Brazil. *ScientificWorldJournal* 2014; **2014:** 586075.
891. Gupta A, Narwal A, Singh H. Primary labial tuberculosis: a rare presentation. *Ann Med Health Sci Res* 2014; **4:** 129-131.
892. Gupta R, Garg M, Gupta AK, Anand C. Tuberculous osteomyelitis of the maxilla: A rarest of rare case report. *Natl J Maxillofac Surg* 2014; **5:** 188-191.
893. Hegde S, Rithesh KB, Baroudi K, Umar D. Tuberculous lymphadenitis: early diagnosis and intervention. *J Int Oral Health* 2014; **6:** 96-98.
894. Jain P, Jain I. Oral Manifestations of Tuberculosis: Step towards Early Diagnosis. *J Clin Diagn Res* 2014; **8:** Ze18-21.
895. Jan SM, Khan FY, Bhat MA, Behal R. Primary tuberculous gingival enlargement - A rare clinical entity: Case report and brief review of the literature. *J Indian Soc Periodontol* 2014; **18:** 632-636.
896. Jiang L, Liu Y, Zhou Y *et al.* [Comparative analysis between CT, MRI and laryngoscopy manifestation for laryngeal tuberculosis.] *Zhonghua Er Bi Yan Hou Tou Jing Wai Ke Za Zhi* 2014; **49:** 771-773. Chinese.
897. Jurado LF, Palacios DM, Alvarez J, Baldion M, Campos G. [Pathological and molecular diagnosis in a case of primary laryngeal tuberculosis in a physician.] *Biomedica* 2014; **34:** 15-20. Spanish.
898. Khuzwayo ZB, Naidu TK. Head and neck tuberculosis in KwaZulu-Natal, South Africa. *J Laryngol Otol* 2014; **128:** 86-90.
899. Kishore DN, Geetha NT, Umashankara KV, Rai KK. Submasseteric tuberculous lesion of mandible: report of a case and review of the literature. *Case Rep Dent* 2014; **2014:** 791630.
900. Lee JW, Ryu KA, Kwon KR, Koo BS. Primary pharyngeal tuberculosis presenting as a submucosal tumour. *Int J Oral Maxillofac Surg* 2014; **43:** 1005-1007.
901. Levian M, Chapman A, Gupta R. Laryngeal tuberculosis: use of videostroboscopy in diagnosis. *Ear Nose Throat J* 2014; **93:** 58.
902. Li L, Lin P, Wei X, Wang W, Zhang S. [Clinical application of endoscopy-assisted resection of tumors in parapharyngeal space.] *Zhonghua Er Bi Yan Hou Tou Jing Wai Ke Za Zhi* 2014; **49:** 986-989. Chinese.
903. Lohler J, Gerstner AO, Bootz F, Walther LE. Incidence and localization of abnormal mucosa findings in patients consulting ENT outpatient clinics and data analysis of a cancer registry. *Eur Arch Otorhinolaryngol* 2014; **271:** 1289-1297.
904. Popescu MR, Calin G, Strambu I *et al.* Lymph node tuberculosis - an attempt of clinico-morphological study and review of the literature. *Romanian journal of morphology and embryology = Revue roumaine de morphologie et embryologie* 2014; **55:** 553-567.
905. Rosado P, Fuente E, Gallego L, Calvo N. Primary tuberculosis of the palate. *BMJ Case Rep* 2014; **2014**.
906. Verma SK, Upadhyay R, Chand P, Tayal N. Tuberculosis: oral alveolus and pulmonary alveoli coexisting. *BMJ Case Rep* 2014; **2014**.
907. Wang M, Zou J, Zheng Y. [Clinical analysis of 20 laryngeal tuberculosis cases.] *Lin Chung Er Bi Yan Hou Tou Jing Wai Ke Za Zhi* 2014; **28:** 1992-1994. Chinese.
908. Ablanedo-Terrazas Y, Alvarado-de la Barrera C, Ruiz-Cruz M, Reyes-Teran G. Mycobacterial cervicofacial lymphadenitis in human immunodeficiency virus-infected individuals after antiretroviral therapy initiation. *Laryngoscope* 2015; **125:** 2498-2502.
909. Aoun N, El-Hajj G, El Toum S. Oral ulcer: an uncommon site in primary tuberculosis. *Aust Dent J* 2015; **60:** 119-122.
910. Bakutra G, Manohar B, Mathur L. Tuberculous osteomyelitis affecting periodontium: A rare case report. *J Indian Soc Periodontol* 2015; **19:** 578-581.
911. Bansal R, Jain A, Mittal S. Orofacial tuberculosis: Clinical manifestations, diagnosis and management. *J Family Med Prim Care* 2015; **4:** 335-341.
912. Basal Y, Ermisler B, Eryilmaz A, Ertugrul B. Two rare cases of head and neck tuberculosis. *BMJ Case Rep* 2015; **2015**.
913. Bayraktar K, Gurer G. Pulmonary tuberculosis presenting with oral aphthae. *Eur J Rheumatol* 2015; **2:** 117-119.
914. Carter E, Chandarana P, Duggineni S, Nasser N, Bridle C. Case series of extra pulmonary tuberculosis presenting as facial swelling. *Br Dent J* 2015; **218:** 519-522.
915. Chen I, Jamal S, Pua KC. Primary tuberculosis of palate. *Malays Fam Physician* 2015; **10:** 32-34.
916. Curto A. Oral tuberculosis lesions. *Br Dent J* 2015; **218:** 662.
917. Darouassi Y, Chihani M, Elktaibi A *et al.* Association of laryngeal and nasopharyngeal tuberculosis: a case report. *J Med Case Rep* 2015; **9:** 2.
918. Gokavarapu S, Panta P. Oral lesions in Tuberculosis. *Pan Afr Med J* 2015; **22:** 336.
919. Hajji F, Beraud G, Genay A *et al.* Extensive Inflammatory Gingival Tumor in a Young Nonsmoking Woman: Back to Basics. *J Craniofac Surg* 2015; **26:** e388-390.
920. Kamath PM, Shenoy VS, M N, Prasad V, Majeed NA. Tuberculosis of Waldeyer's Ring with an Atypical Presentation as Chronic Adeno-Tonsilitis. *J Clin Diagn Res* 2015; **9:** Md01-02.
921. Krawiecka E, Szponar E. Tuberculosis of the oral cavity: an uncommon but still a live issue. *Postepy Dermatol Alergol* 2015; **32:** 302-306.
922. Kurokawa M, Nibu K, Ichimura K, Nishino H. Laryngeal tuberculosis: A report of 17 cases. *Auris Nasus Larynx* 2015; **42:** 305-310.
923. Llorente Romano M, Pasamontes Pingarron JA, Molina Molina MC, Temboury Molina MC. [Laryngeal tuberculosis and coadjuvant corticosteroid treatment.] *An Pediatr (Barc)* 2015; **82:** e209-210. Spanish.
924. Lodha JV, Sharma A, Virmani N, Bihani A, Dabholkar JP. Secondary laryngeal tuberculosis revisited. *Lung India* 2015; **32:** 462-464.
925. Lucena MM, da Silva Fdos S, da Costa AD *et al.* Evaluation of voice disorders in patients with active laryngeal tuberculosis. *PLoS One* 2015; **10:** e0126876.
926. Mahfoudhi M, Khamassi K, Turki S, Kheder A. [Diagnostic difficulty of isolated laryngeal tuberculosis in a diabetic woman.] *Pan Afr Med J* 2015; **21:** 106. French.
927. Mehta KD, Haran A, Harsha A, Gulikari GK, Kaul R. An aggressive, solitary non-healing ulcer: Not always cancerous. *Respir Med Case Rep* 2015; **15:** 133-134.
928. Namdev R, Jain M, Jindal A, Bodh M. Tuberculosis of the Cheek: A Rare Presentation. *J Clin Pediatr Dent* 2015; **39:** 475-480.
929. Nemes RM, Ianosi ES, Pop CS *et al.* Tuberculosis of the oral cavity. *Rom J Morphol Embryol* 2015; **56:** 521-525.
930. Ohki M, Komiyama S, Tayama N. Pharyngolaryngeal paralysis in a patient with pharyngeal tuberculosis. *Auris Nasus Larynx* 2015; **42:** 63-67.
931. Okano Y, Yoshida S, Shinohara T *et al.* Primary Gingival Tuberculosis Diagnosed Based on Genetic Identification. *Intern Med* 2015; **54:** 2765-2768.
932. Silas OA, Ige OO, Adoga AA, Nimkur LT, Ajetunmobi OI. Role of Fine Needle Aspiration Cytology (FNAC) as a Diagnostic Tool in Paediatric Head and Neck Lymphodenopathy. *J Otol Rhinol* 2015; **4**.
933. Smaoui S, Mezghanni MA, Hammami B *et al.* Tuberculosis lymphadenitis in a southeastern region in Tunisia: Epidemiology, clinical features, diagnosis and treatment. *Int J Mycobacteriol* 2015; **4:** 196-201.
934. Spini RG, Bordino L, Cohen D, Martins A, Ramirez Z, Gonzalez NE. [Pharyngeal tuberculosis: Case report.] *Arch Argent Pediatr* 2015; **113:** e230-233. Spanish.
935. Taute RB, Wylie J, Carter L. An Unusual Extranodal Presentation of Mycobacterium Tuberculosis in the Upper Lip. *Dent Update* 2015; **42:** 473-475.
936. van der Sar-van der Brugge S, Akkerman OW, van der Laan BF, de Lange WC, van der Werf TS. [A sore throat: tumour, tuberculosis or both?] *Ned Tijdschr Geneeskd* 2015; **159:** A8942. Dutch.
937. Wang PH, Wang HC, Liao CH. Disseminated Penicillium marneffei mimicking paradoxical response and relapse in a non-HIV patient with pulmonary tuberculosis. *J Chin Med Assoc* 2015; **78:** 258-260.
938. Yin N, Delord M, Giovanni A *et al.* Laryngeal tuberculosis diagnosed by stool sample cultures: a case report. *J Med Case Rep* 2015; **9:** 74.
939. Zavod MB. Laryngeal tuberculosis: A public health concern. *Ear Nose Throat J* 2015; **94:** E51-52.
940. Amaya-Tapia G, Rodriguez-Toledo A, Aguilar-Benavides S, Aguirre-Avalos G. Large Retropharyngeal Abscesses in an Immunocompetent Adult Patient with Disseminated Tuberculosis. *Am J Case Rep* 2016; **17:** 690-693.
941. Bricha M, Slimani H, Hammi S, Bourkadi JE. [Tuberculous cheilitis revealing pulmonary tuberculosis.] *Pan Afr Med J* 2016; **24:** 176. French.
942. Faisal M, Harun H, Hassan TM, Ban AY, Chotirmall SH, Abdul Rahaman JA. Treatment of multiple-level tracheobronchial stenosis secondary to endobronchial tuberculosis using bronchoscopic balloon dilatation with topical mitomycin-C. *BMC Pulm Med* 2016; **16:** 53.
943. Helman SN, Karle W, Pitman MJ. Management of Posterior Glottal Insufficiency With Use of a Buccal Graft. *Ann Otol Rhinol Laryngol* 2016.
944. Ho UC, Chen CN, Lin CY *et al.* Application of ultrasound-guided core biopsy to minimize the non-diagnostic results and the requirement of diagnostic surgery in extrapulmonary tuberculosis of the head and neck. *Eur Radiol* 2016; **26:** 2999-3005.
945. Jain P, Puwar P. Primary Tuberculosis of Tongue. *J Assoc Physicians India* 2016; **64:** 78-79.
946. Matsumoto K, Komukai J, Tsuda Y *et al.* [Use of QuantiFeron(R) TB-Gold in-tube in a contact investgation to determine the onset of tuberculosis with or without lantent tuberculosis infection treatment.] *Kekkaku* 2016; **91:** 45-48. Japanese.
947. Mouhsine A, Temsamani H, Belkouch A, Atmane E, Elfikri A, Benariba F. Pharyngeal tuberculosis: Report of 5 cases. *Acta Otorrinolaringol Esp* 2016; **67:** 162-166.
948. Reis JG, Reis CS, da Costa DC *et al.* Factors Associated with Clinical and Topographical Features of Laryngeal Tuberculosis. *PLoS One* 2016; **11:** e0153450.
949. Silva DS, Dawson A, Upshur RE. Reciprocity and Ethical Tuberculosis Treatment and Control. *J Bioeth Inq* 2016; **13:** 75-86.
950. Wu YH, Chang JY, Sun A, Chiang CP. Oral tuberculosis. *J Formos Med Assoc* 2016.
951. Xu JJ, Peer S, Papsin BC, Kitai I, Propst EJ. Tuberculous lymphadenitis of the head and neck in Canadian children: Experience from a low-burden region. *Int J Pediatr Otorhinolaryngol* 2016; **91:** 11-14.
952. Zang J, Liu Q, Jiang XJ. [The clinical and pathological features of laryngeal tuberculosis.] *Zhonghua Jie He He Hu Xi Za Zhi* 2016; **39:** 612-615. Chinese.
953. Zhang X, Wang J, Wu Y *et al.* Tuberculosis with atypical manifestations involving multiple sites of the oral cavity: A case study. *Indian J Dermatol Venereol Leprol* 2017; **83:** 116-118.
